# Nothing to Sneeze At: A Dynamic and Integrative Computational Model of an Influenza A Virion

**DOI:** 10.1016/j.str.2014.12.019

**Published:** 2015-03-03

**Authors:** Tyler Reddy, David Shorthouse, Daniel L. Parton, Elizabeth Jefferys, Philip W. Fowler, Matthieu Chavent, Marc Baaden, Mark S.P. Sansom

**Affiliations:** 1 Department of Biochemistry, University of Oxford, South Parks Road, Oxford OX1 3QU, UK; 2 Institut de Biologie Physico-Chimique, Centre National de la Recherche Scientifique, UPR9080, Université Paris Diderot, Sorbonne Paris Cité, Paris, France

## Abstract

The influenza virus is surrounded by an envelope composed of a lipid bilayer and integral membrane proteins. Understanding the structural dynamics of the membrane envelope provides biophysical insights into aspects of viral function, such as the wide-ranging survival times of the virion in different environments. We have combined experimental data from X-ray crystallography, nuclear magnetic resonance spectroscopy, cryo-electron microscopy, and lipidomics to build a model of the intact influenza A virion. This is the basis of microsecond-scale coarse-grained molecular dynamics simulations of the virion, providing simulations at different temperatures and with varying lipid compositions. The presence of the Forssman glycolipid alters a number of biophysical properties of the virion, resulting in reduced mobility of bilayer lipid and protein species. Reduced mobility in the virion membrane may confer physical robustness to changes in environmental conditions. Our simulations indicate that viral spike proteins do not aggregate and thus are competent for multivalent immunoglobulin G interactions.

## Highlights

• Microsecond timescale molecular dynamics simulations of enveloped influenza virion
• Forssman glycolipid alters the biophysical properties of the influenza A virion
• Spacing of the spike glycoproteins is compatible with bivalent antibody association
• Mobility of viral proteins influences distribution of lipids in the viral envelope

## Graphical Abstract

**Figure:**
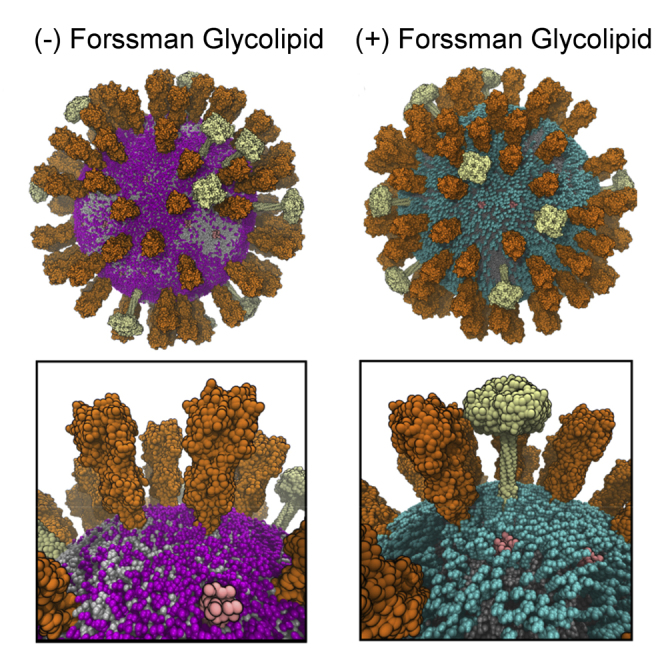

## Teaser

Reddy et al. performed the first microsecond timescale coarse-grained molecular dynamics simulations of enveloped virions in explicit solvent. The simulated properties of the influenza A virion were consistent with experimental measurements, and revealed that the Forssman glycolipid affects several biophysical characteristics of the virion.

## Introduction

There have been a number of structural studies on the influenza A virus (e.g. Calder et al., 2010; Harris et al., 2006; Wasilewski et al., 2012), which is surrounded by a pleomorphic lipid bilayer envelope that imposes challenges for high-resolution structural characterization. These have provided important details about the morphology of the virions and the distribution of their surface glycoproteins, but structural studies that include detailed analysis of the lipids are lacking. Indeed, the lipid composition of the influenza A envelope has only recently been established (Gerl et al., 2012). The importance of lipids in the stability of the influenza A virion is clear from a number of studies. Both H5N1 and H1N1 viruses were more stable in water when grown in mammalian cells versus counterparts propagated in avian cells, even for viruses with the same genetic background (Shigematsu et al., 2014). Only the lipid composition and the glycosylation states of the viruses differed. A progressive ordering with decreasing temperature for influenza A lipids studied by nuclear magnetic resonance (NMR) spectroscopy implicated the lipids in seasonal behavior (Polozov et al., 2008). Lipids form much of the outer protective shell of the influenza A virion, and they are a logical target for additional biophysical analysis.

Molecular dynamics simulations provide an opportunity to integrate structural data from a variety of experimental sources. For example, an impressive set of 0.1 μs, 64 million atom, molecular dynamics simulations were used to model the HIV-1 capsid (Zhao et al., 2013). However, these simulations omitted the lipid envelope of the virus, enabling the method for model construction to be strongly guided by the experimental electron densities from cryo-electron microscopy (cryo-EM). A multiscale approach was used for examining the full-scale immature HIV-1 virion (Ayton and Voth, 2010). The system was highly coarse-grained (CG) with a protein model corresponding to approximately 7–9 amino acid residues per particle, and used a relatively simple (DOPS/DOPC) and symmetric lipid bilayer membrane. An all-atom simulation of a complete virus, including its RNA core, has also been performed (Freddolino et al., 2006), based on the crystal structure of satellite tobacco mosaic virus. This virus contains no lipid, and the viral envelope consists of 60 copies of a single protein arranged in an icosahedron. Recent modeling of nonenveloped icosahedral virions revealed their mechanical properties and possible mechanisms for capsid dissolution via calcium ion depletion (Larsson et al., 2012; Zink and Grubmüller, 2009, 2010). Likewise, recent modeling of the rabbit hemorrhagic disease virus (Wang et al., 2013), which is also icosahedral and contains no lipids, was based on fitting the model to available X-ray diffraction and cryo-EM data. Previous influenza virus membrane protein simulations have largely been focused on isolated components of the virion, e.g. modeling of fusion peptide activity (Risselada et al., 2012) or of hemagglutinin (HA) clustering in model membranes (Parton et al., 2013).

In this study, we use CG molecular dynamics simulations (Stansfeld and Sansom, 2011) building on structural information from X-ray crystallography (Ha et al., 2003; Varghese and Colman, 1991), NMR spectroscopy (Schnell and Chou, 2008), cryo-EM (Harris et al., 2006), and lipidomics data (Gerl et al., 2012) to produce a detailed (near atomic resolution) computational model of the influenza A virion. This integration of structural information from a number of sources has allowed us to perform microsecond-scale CG molecular dynamics simulations of the outer envelope of an enveloped virion in explicit solvent. These simulations reveal the structural and dynamic properties of the viral envelope which contribute to its stability, and will allow us to initiate models of virion/target cell recognition. The complex lipid dynamics revealed in our simulations extend and complement static structural data from cryo-EM and related experimental approaches. We provide the virion coordinates and simulation parameters openly to the community.

## Results and Discussion

### Influenza Virion Models and Progress of the Simulations

The construction of a computational model of the membrane envelope of a virion is in itself challenging and warrants some discussion. More generally, generating computational models of pleomorphic enveloped viruses, for which high-resolution structural detail of the entire virion is more difficult to obtain, has not been described in detail. The essence of the problem is to model a “moving target” at the same time as retaining available structural data, rather than fitting to reasonably well defined experimental density.

**Figure 1:**
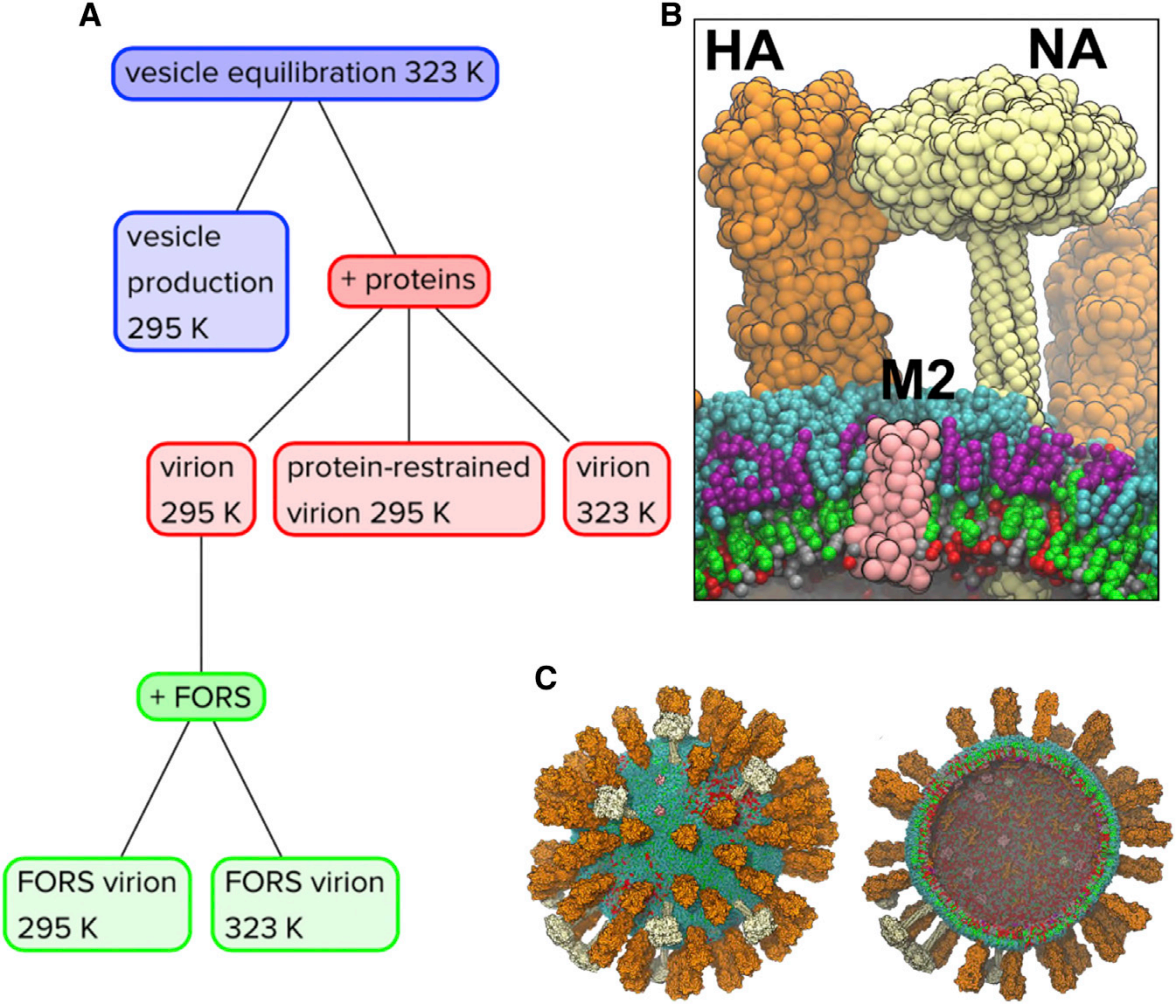
Influenza Virion Models and Simulations (A) Flowchart summary of the influenza A virion simulations. The initial vesicle equilibration simulation was for 0.3 μs; all other simulations were for ∼5 μs. The lipid species used include palmitoyl-oleoyl-phosphatidylserine (POPS), dioleoylphosphatidylethanolamine (DOPE), ether-linked DOPE (DOPX), cholesterol (CHOL), hydroxylated sphingomyelin (PPCH), and the Forssman glycolipid (FORS). All virion simulations including proteins contained 80 HA trimers, 12 NA tetramers, and 15 M2 tetramers. A restrained inner core of 31,767 particles was assembled into a hollow sphere to represent the approximate volume occupied by the nucleoprotein. The systems also contained ∼5% antifreeze water. (B) Zoom-in view of a representative region from the FORS-inclusive virion simulation at 323 K showing the HA (orange), NA (white), and M2 (pink) viral proteins in the context of the multicomponent asymmetric lipid envelope (CHOL: green; DOPE/X: red; FORS: cyan; POPS: silver; PPCH: purple). (C) The starting configuration of the 295 K virion simulation. An outside view (left) and a cross-sectional view (right) are shown, the central core having been omitted from the latter diagram for clarity. See also Figures S1–S3 and S18. See also Supplemental Information Files S20–S24 for coordinate files corresponding to the virus models at the end of each of the five simulations.

We started by generating an initial lipid bilayer vesicle model (Figure 1A), with a diameter of ∼74 nm and with a lipid bilayer composition approximating the known viral lipidome (Gerl et al., 2012). This model includes the following lipid species: 15% palmitoyl-oleoyl-phosphatidylserine (POPS), 5% dioleoylphosphatidylethanolamine (DOPE), 9% ether-linked DOPE (DOPX), 53% cholesterol, and 18% hydroxylated sphingomyelin (PPCH), with a total of ∼43,000 lipid molecules present. Using the MARTINI CG model (which has been widely used to model and simulate a wide range of membrane proteins and systems [Marrink and Tieleman, 2013]) this yields a simulation system of >5M particles (full coordinates available in Supplemental Information). We used a spherical hollow core of positionally restrained “RPO” particles to substitute for the ribonucleoprotein (RNP) core of the virion in all simulations. The shape of the virion would be anticipated to remain spherical in the absence of the RPO core. Previous CG simulations of vesicles of up to 20 nm outer diameter demonstrated near perfect sphericity on a microsecond timescale (Marrink and Mark, 2003), and short CG simulations of 190 nm outer diameter liposomes were stable in the absence of an internal core (Ayton and Voth, 2009a).

**Figure 2:**
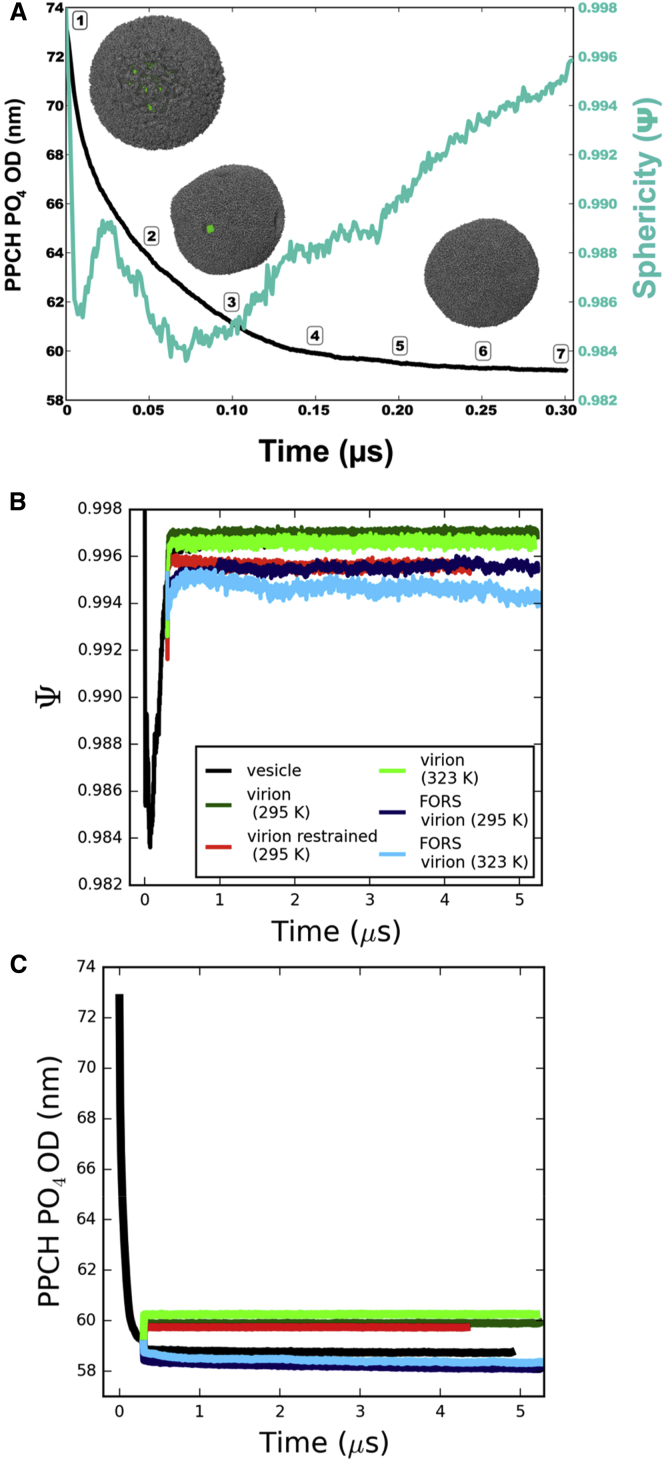
Progress of the Simulations (A) Progress of the 0.3 μs vesicle equilibration simulation, tracking the outer diameter (black line, left-hand axis) and sphericity (cyan, right-hand axis) as functions of time. The outer diameter is calculated as twice the average radius of all hydroxylated sphingomyelin headgroup phosphate (PPCH PO_4_) particles from the lipid centroid of the system. Seven time points are marked in 50 ns intervals. The inset images of the equilibrating vesicle (at time points 1, 3, and 6) have lipids shown in gray and the central core in green. (B and C) Progress of the virion model simulations was also monitored by tracking sphericity (B) and outer diameter (C). The vesicle (black, temperature adjusted from 323 to 295 K at 0.3 μs), the virion (295 K, dark green; 323 K, light green), the protein-restrained virion (red), and the Forssman glycolipid inclusive virion (295 K, dark blue; 323 K, light blue) are shown. See also Figures S4 and S5.

The initial vesicle model was simulated for 0.3 μs to equilibrate the packing of the lipids. There was a rapid (∼50 ns) relaxation that resulted in shrinking of the vesicle (the lipids were initially loosely packed) and “repairing” of holes in the lipid bilayer. Thus, the outer diameter (Figure 2A) dropped from ∼74 nm to ∼59 nm, attaining a final size consistent with the lower bounds estimated from experiments discussed below. Monitoring the sphericity (*Ψ*) (Wadell, 1935) of the equilibrating vesicle revealed that there was a degree of deformation during the initial shrinking followed by a return to a more spherical shape (*Ψ* = 1) by the end of the equilibration period. As the shrinking of the vesicle is a nonequilibrium process, we do anticipate some pressure difference between the inside and outside of the structure, and the resulting membrane tensions may influence lipid dynamics.

**Figure 3:**
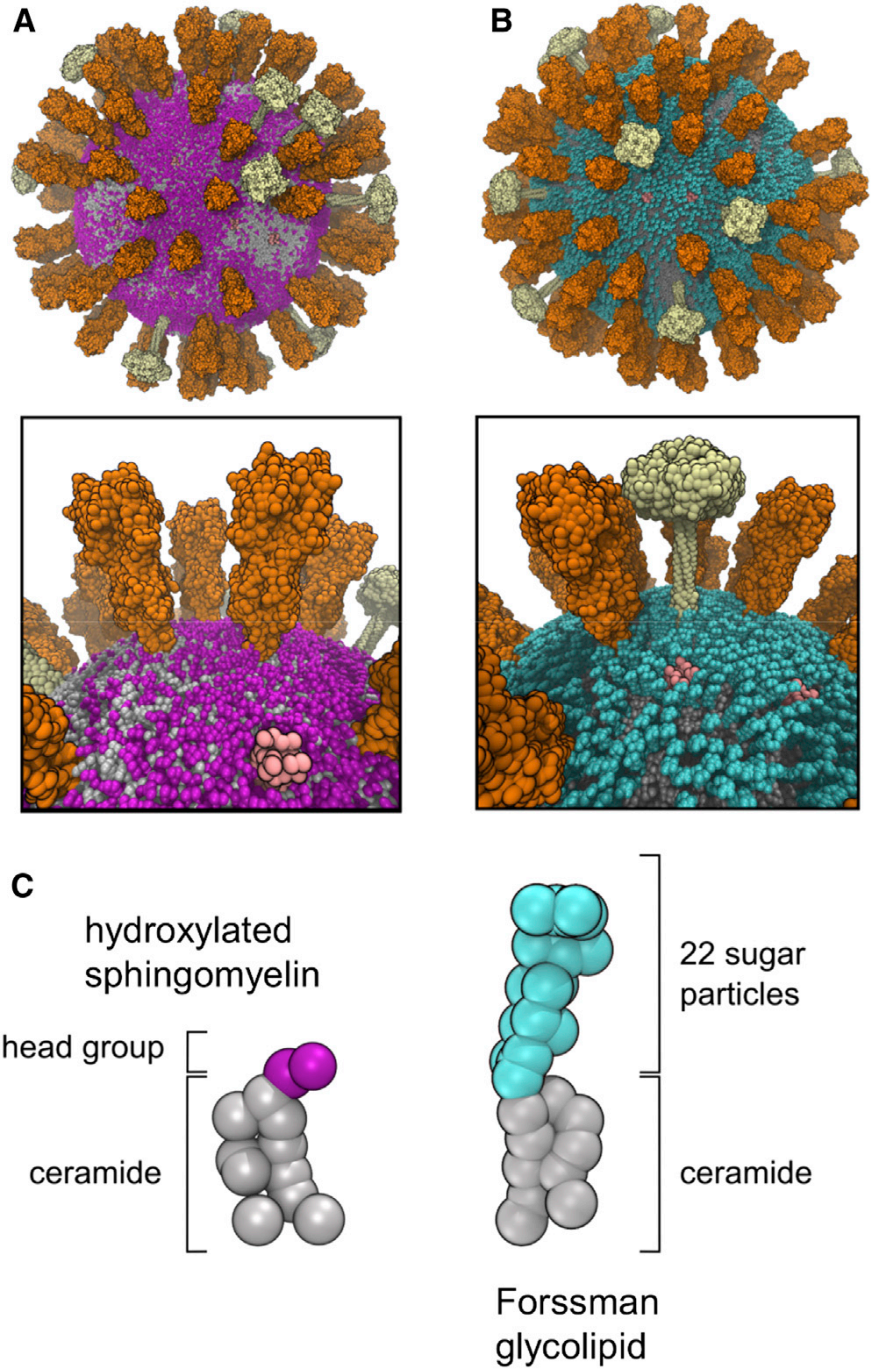
Virion Models and Glycolipids (A and B) Visual comparison of virion starting models without (A) and containing (B) the Forssman glycolipid (*cyan*). Overall and zoom-in views of the respective virions are shown. (C) Comparison of the CG representations of a hydroxylated sphingomyelin and a Forssman glycolipid. See also Figure S3.

The three species of influenza A integral membrane proteins (Figure 1B) were then inserted into the vesicle (Figure S1) to yield an initial virion model containing 80 hemagglutinin (HA) trimers, 12 neuraminidase (NA) tetramers, and 15 M2 tetramers. The copy numbers of HA and NA proteins were derived from their surface density in 3D tomograms of the virion (Harris et al., 2006). The number of M2 proteins derived from the lower bound of an experimental estimate of 15–25 channels per virion (Takeda et al., 2002). The HA models were palmitoylated at their C termini (Parton, 2011) (see also Supplemental Information). In two of the virion simulations, Forssman glycolipid (globopentosylceramide) was included in the outer leaflet of the lipid bilayer (Figures 1A and 3; Figure S2), resulting in an overall composition of 6% hydroxylated sphingomyelin (PPCH) and 12% Forssman glycolipid. The Forssman glycolipid is the most abundant sphingolipid in the influenza A virion (Gerl et al., 2012), with a ∼2-fold greater abundance than sphingomyelin. The Forssman glycolipid has a terminal *N*-acetylgalactosamine in α1-3 linkage to the terminal *N*-acetylglucosamine of globoside (Stanley and Cummings, 2009).

**Figure 4:**
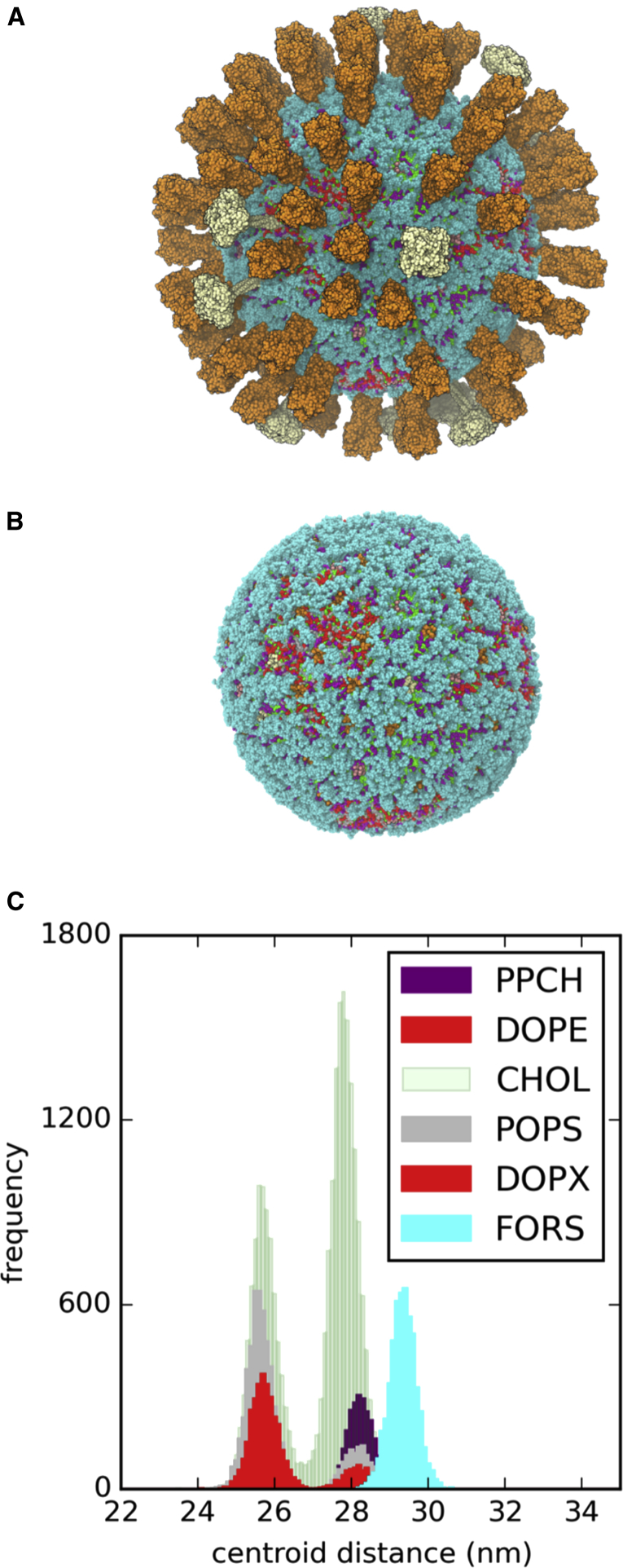
Snapshot at the End of the 295 K + Forssman Glycolipid Virion Simulation (A and B) Protein ectodomains are shown in (A) and omitted for clarity in (B) (HA orange, NA white, M2 pink). The glycolipid is shown in cyan. (C) The composition of lipid species and their distributions between the inner and outer leaflets of the bilayer are shown as a histogram of centroid distances from the overall virion centroid, with the colors approximately matched to those used for lipids in the structural snapshots.

The resultant membrane is relatively crowded, although a little sparser than for some cell membranes, e.g. ca. 25% of the area of red blood cell membranes is estimated to be protein (Dupuy and Engelman, 2008). Calculating the “fractional volume” of a spherical shell corresponding to the ectodomains of the spike proteins (see below for details) indicates they occupy ca. 15% of the viral membrane area (Wasilewski et al., 2012); thus the proteins do not completely cover the surface of the virion. By visual inspection, the Forssman glycolipid largely “covers” the bilayer surface (Figure 4B). In particular, the glycolipid largely masks the M2 channel proteins from the external surface of the virion. This may have implications in terms of the access of drug molecules (adamantanes), which act by blocking the M2 channel protein (Gu et al., 2013).

Based on the vesicle and virion models, a number of simulations were performed (Figure 1A; Figure S3). These explored both room (295 K) and elevated (323 K) temperatures, and also the presence or absence of the Forssman glycolipid (Figure 3), which is found in the influenza lipidome instead of 68% of the outer leaflet hydroxylated sphingomyelin molecules. We also performed a simulation in which the centroids of the membrane proteins were restrained, to mimic possible interactions with the inner matrix formed by the M1 matrix proteins of the virus (Veit and Thaa, 2011). However, it is useful to note that successful budding of approximately 10% of influenza A virions occurs with an apparent lack of the M1 layer, indicating that the simulations without protein restraints are of biological relevance, especially to the low pH, M1 detached, fusion-compatible state of the virus (Fontana and Steven, 2013). Thus we have what is perhaps the most realistic current computational model of an influenza A virion tractable for microsecond timescale simulation (Figure 4).

The progress of the simulations was monitored, including the sphericity and diameters of the lipid bilayer components of the models. In all cases, these values were consistent with an overall spherical geometry being retained, with some degree of influence on the shape of the virion caused by the presence or absence of the Forssman glycolipid (Figure 2B). Restriction of protein mobility via restraints caused a similar reduction in sphericity to the presence of glycolipid. This suggests a possible role for protein-lipid interactions in the shaping of the virion.

The diameters of the virion models were also all stable on a microsecond timescale (Figure 2C). Interestingly, virions were substantially smaller when glycolipid was present in place of sphingomyelin. This suggests that it is important to include glycolipids when constructing computational models of enveloped virions. We measured virion outer diameters using lipids, as they have a higher sampling density than the proteins. The range of simulation diameters (58–61 nm) for the lipid components of all the virion simulations was consistent with the experimentally determined minimum virion diameter of 84 nm (Harris et al., 2006), after taking into account the 10 nm to 14 nm lengths of the two spike proteins (Schmitt and Lamb, 2005).

We also assessed the models on the basis of the extent of solvent penetration into the lipid envelope (Figure S4). In all cases, the degree of solvent particle penetration into the hydrophobic core of the lipid bilayer is low. This is clearest for the lipid (no protein) vesicle, while slightly more water penetrates in the case of the virion models. Interestingly it has been shown that enveloped vesicular stomatitis virions are more sensitive to osmotic stress before protease treatment (Bittman et al., 1976), suggesting that viral membrane proteins may play a role in maintaining envelope permeability.

**Figure 5:**
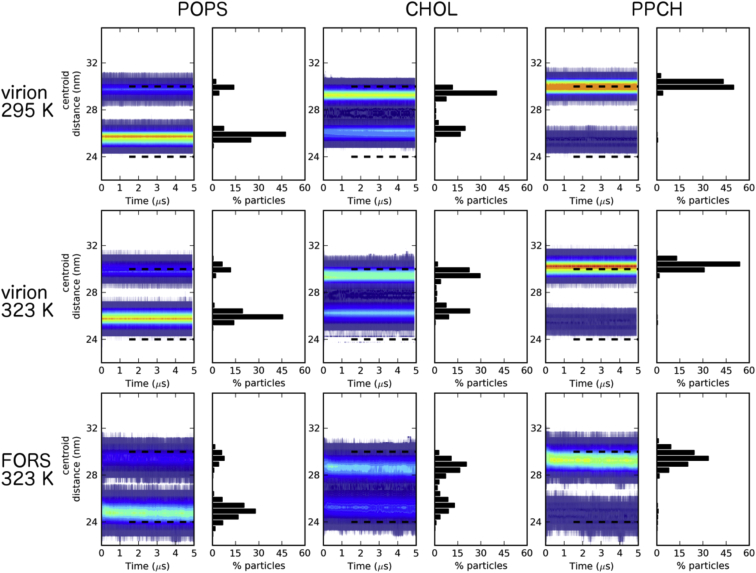
Distribution of Selected Lipids between the Outer and Inner Leaflets of the Bilayer Heatmaps are shown for the time-dependent distributions of the distances between lipid species and the virion centroid. These are shown for representative inner leaflet (POPS, left), central (CHOL, middle), and outer leaflet species (PPCH, right). Phosphate particles were used for POPS and PPCH, while the ROH group was used for calculations with CHOL. The histogram data from the last analyzed frame of each replicate is shown to the right of each contour plot. See also Figures S6 and S17.

We measured the distribution of the lipids between the two bilayer leaflets in each simulation, and observed stable leaflet populations for, e.g., PS as a representative inner leaflet lipid species, hydroxylated sphingomyelin as a representative outer leaflet lipid species, and cholesterol (Figure 5; Figures S5 and S6). In the presence of glycolipid or positional restraints on the membrane proteins a noticeably broader distribution of lipids was seen, indicating possible lipid mixing between leaflets. It is interesting that whether the membrane protein dynamics are reduced by restraining their motion directly or by inclusion of glycolipids (see discussion of diffusion coefficients below) the same effect is observed, suggesting that relatively immobile proteins may allow some degree of local perturbation of the structure of the lipid bilayer.

### Diffusion of Lipids and Proteins

The influenza virion membrane provides an example of a complex biological membrane, differing from simpler model vesicle systems (Gambin et al., 2006) in that it contains multiple protein molecules and a complex mixture of lipid species, including a high (>50%) fraction of cholesterol. It is therefore of particular interest to use simulations to probe the diffusion dynamics of the proteins and lipids. Protein and lipid translational diffusion parameters were calculated from mean square displacement (MSD) versus time data, interpreted either with a “normal” diffusion model (MSD = 4*Dt*) or via an anomalous diffusion model in which the fractional diffusion constant (*D*_*α*_) and scaling exponent (*α*) were calculated from two-parameter nonlinear fits to the MSD versus time data (MSD = 4*D*_*α*_*t*^*α*^), as described previously (Kneller et al., 2011). In the latter approach, anomalous diffusion is defined as *α* ≠ 1. Subdiffusion (*α* < 1) has been documented in experimental and computational studies of lipid bilayers (Almeida and Vaz, 1995; Banks and Fradin, 2005; Feder et al., 1996; Jacobson et al., 1987).

**Figure 6:**
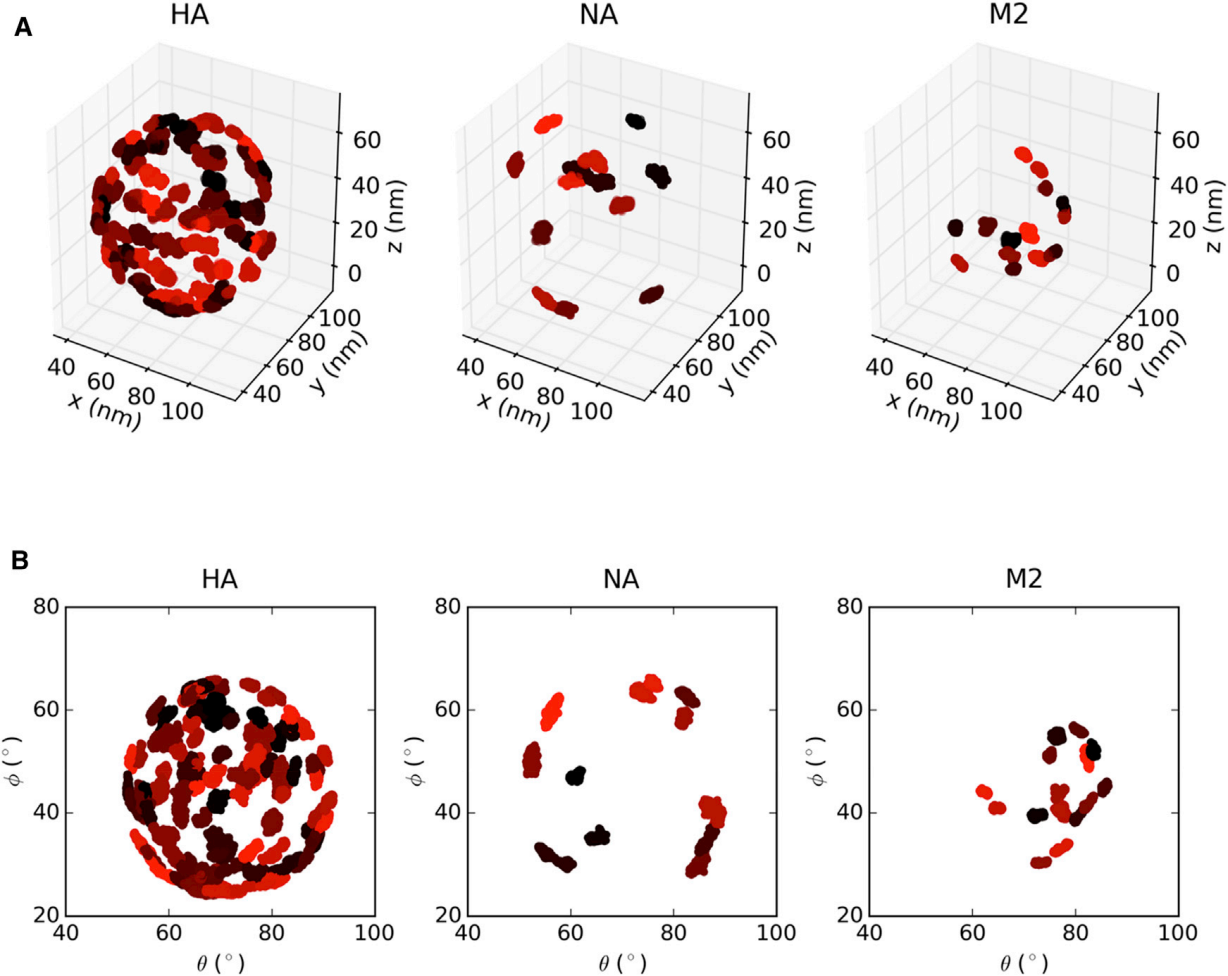
Protein Mobility during the Simulation (A and B) The centroids of all 107 influenza membrane proteins were tracked over the duration of the virion simulation at 295 K excluding the Forssman glycolipid. The results are shown in Cartesian coordinate space (A) or in 2D spherical polar projection space (B). Each individual protein centroid is colored in a different shade for each of the three protein types. See also Figure S8.

**Figure 7:**
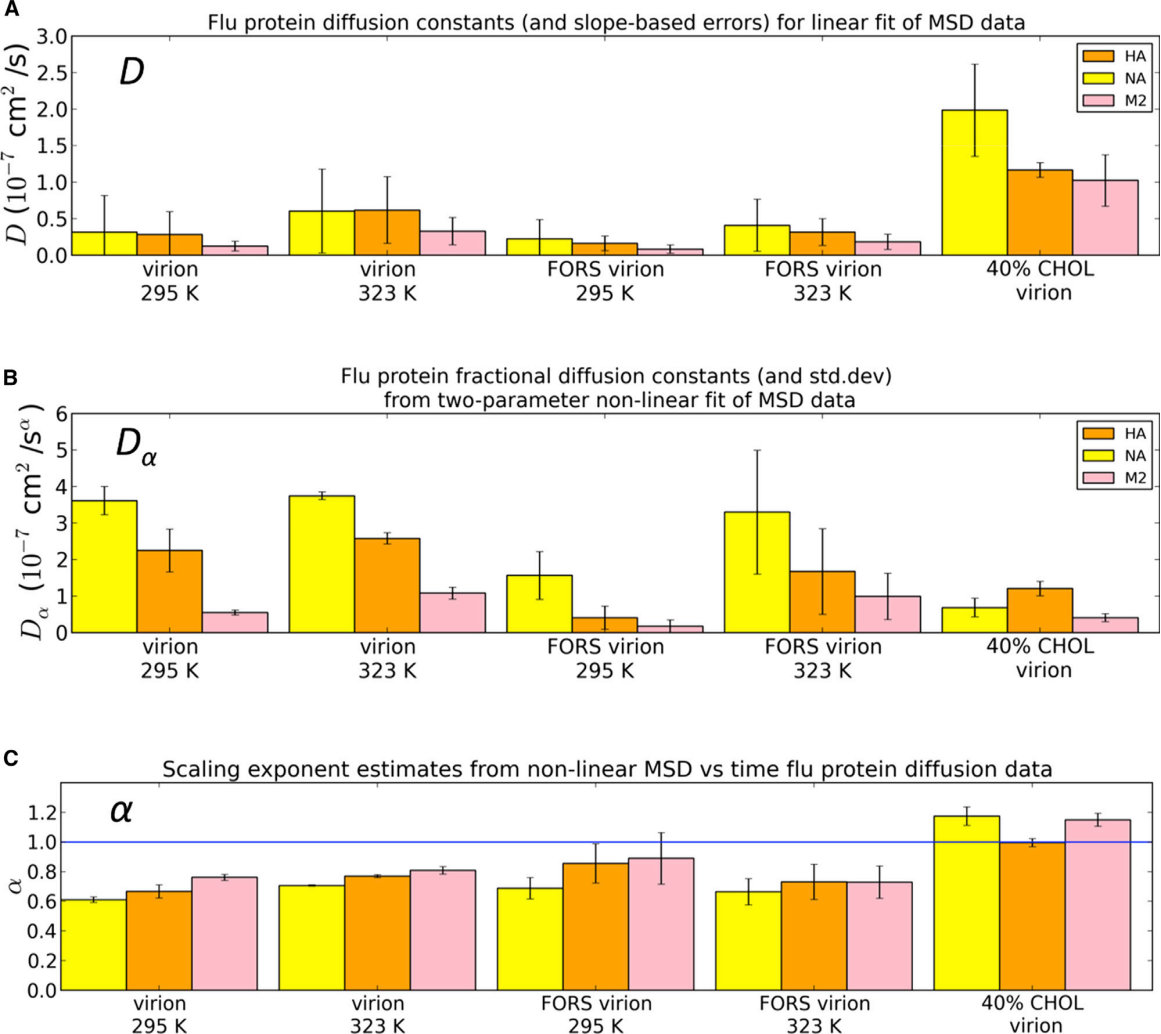
Protein Diffusion Coefficients (A–C) Protein diffusion coefficients calculated for either (A) linear (MSD = 4*Dt*) or (B) nonlinear (MSD = 4*D*_*α*_*t*^*α*^) diffusion models. The scaling exponents (C), *α*, were also calculated for the nonlinear fit to the data, with values <1 consistent with anomalous subdiffusion and values ≅1 consistent with random-walk diffusion. SD values were extracted from two-parameter fits for the nonlinear analysis while uncertainties in the linear data were estimated as the difference between the slopes of the first and final halves of the MSD versus time data. See also Figure S9.

The M2 protein consistently had an average diffusion constant lower than the other proteins (HA and NA; Figures 6 and 7), regardless of whether the MSD data were fitted to a normal or an anomalous diffusion model. The general order of diffusion coefficients was NA ≥ HA > M2. This may reflect a combination of the cross-sectional areas of the TM domains and the strength of their lipid interactions. M2 diffused very slowly (4.4 × 10^−14^ cm^2^/s, as measured by atomic force microscopy) in a supported gel phase dipalmitoyl phophatidylcholine bilayer (Hughes et al., 2004). In addition to the simulations discussed above, we also performed simulations in a simplified model envelope containing only three lipid species and a lower cholesterol content (40%; Figure S7). In these lower cholesterol simulations the diffusion constant of M2 (for the normal diffusion model) was closer to that of HA than for other conditions. Furthermore, while the virion models exhibited subdiffusion (mean *α* ∼0.8), the low (40%) cholesterol model exhibited approximately normal protein diffusion. The subdiffusive behavior of simple model transmembrane proteins embedded in a bilayer has previously been demonstrated using CG simulations (Schmidt and Weiss, 2011). Thus, the biologically realistic membrane lipid model leads to anomalous diffusion of the influenza virion proteins. As expected, temperature has an effect on diffusion coefficients—they are lower at room temperature (295 K) than at elevated temperature (323 K) (Figure S8). Perhaps less expected is the effect of the Forssman glycolipid, which substantially reduced protein diffusion coefficients.

If we take, e.g., HA in the FORS virion at 323 K, we obtain a mean value *D* of ∼0.3 × 10^−7^ cm^2^/s. In a recent study of membrane protein diffusion in crowded bilayers using CG simulations and a simple (PE/PG) lipid bilayer (Goose and Sansom, 2013), translational diffusion coefficients for individual bacterial outer membrane proteins (OMPs) varied between 0.5 and 3 × 10^−7^ cm^2^/s, falling below 0.5 × 10^−7^ cm^2^/s for crowded bilayers. Thus, the protein diffusion rates in the +FORS virion models are comparable with those in simple models of crowded lipid bilayers. Fluorescence recovery after photobleaching (FRAP) studies of crowded membrane proteins in reconstituted giant unilamellar vesicles (GUVs) revealed protein diffusion coefficients (0.4 × 10^−7^ cm^2^/s) (Ramadurai et al., 2009) that match our simulation-based values for the +FORS virion. However, FRAP measurements of HA diffusion on the surfaces of infected cells yielded much lower *D* values in the range 10^−10^ to 10^−9^ cm^2^/s (Shvartsman et al., 2003), possibly reflecting cytoskeletal interactions of lipid rafts. We previously reported diffusion coefficients of single CG HA molecules of *D* ∼ 10^−7^ cm^2^/s in bilayer patches with 35% cholesterol and of *D* ∼ 0.3 × 10^−7^ cm^2^/s with no cholesterol (Parton et al., 2013). Thus, previously reported CG simulation diffusion constants are reasonably consistent with our current range of virion-based diffusion constants given the differences in protein concentration, lipid composition, and bilayer geometry.

**Figure 8:**
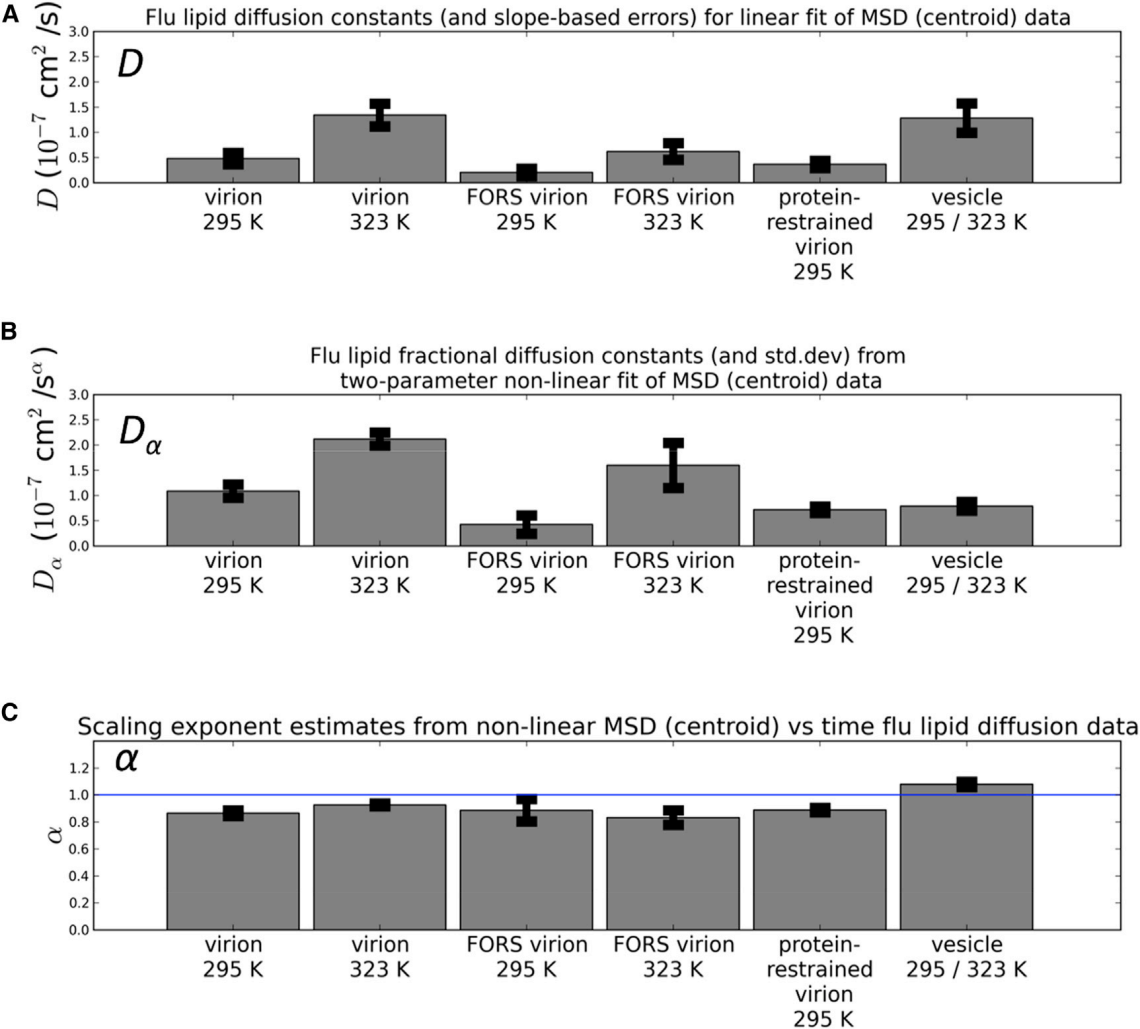
Lipid Diffusion Coefficients (A–C) Lipid diffusion coefficients calculated for either (A) linear (MSD = 4*Dt*) or (B) nonlinear (MSD = 4*D*_*α*_*t*^*α*^) diffusion models and their reported uncertainties were averaged across all lipid species for a given simulation condition. The scaling exponents (C), *α*, were also calculated for the nonlinear fit to the data. The uncertainty metrics are as described in Figure 7, and the full diffusion analysis results for all lipid species in all simulation conditions is available in the Supporting Information. See also Figure S10.

The lipid (Figure 8; Figures S9 and S10) *D* values ranged from ∼0.2 × 10^−7^ to ∼7 × 10^−7^ cm^2^/s. This range is consistent with that reported in a recent CG study of simple model systems (Goose and Sansom, 2013) discussed above, where phospholipid diffusion coefficients of *D* = 8.5 × 10^−7^ cm^2^/s were observed in the absence of protein, falling to ∼4.0 × 10^−7^ cm^2^/s at a high protein concentration. We also note that FRAP studies of crowded GUVs yielded lipid diffusion coefficients of ∼1 × 10^−7^ cm^2^/s (Ramadurai et al., 2009), again consistent with our results. Solid-state NMR spectroscopy has been used to measure the average diffusion of reconstituted influenza lipid mixtures, with *D* ranging from 0.7 to 3.5 × 10^−7^ cm^2^/s as temperature was varied from 290 to 310 K (Polozov et al., 2008). Thus, experimental and computational measurements of diffusion rates for comparable lipid compositions are in reasonable agreement, although given our observation of anomalous (subdiffusive) behavior (see below), one should exercise caution in making such comparisons. As expected, lipid diffusion constant values increase with temperature. At both temperatures, the presence of the Forssman glycolipid resulted in a substantial decrease in *D* values for all of the lipids present. In all cases, the Forssman glycolipid was on average the least mobile of the lipid species under similar conditions. Restraining the proteins (see above) also lowers all lipid diffusion coefficients. Overall, the lowest average lipid diffusion rates were for the virion including Forssman glycolipids at room temperature (295 K), as was the case for the proteins. Similar to the proteins, in most simulations the lipids exhibited subdiffusive (*α* < 1) behavior, with on average *α* ∼ 0.9. FRAP studies of crowded GUVs demonstrated anomalous lipid diffusion (*α* = 0.9) at higher degrees of membrane crowding (Ramadurai et al., 2009). Previous simulation results suggest that increasing protein concentration in membranes leads to the onset of anomalous diffusion (Javanainen et al., 2013), and *α* dropped from approximately 1 to 0.8 over the range of increasing protein concentration used in the recent CG study of a simple model discussed above (Goose and Sansom, 2013). Subdiffusion was not observed in the vesicle simulations.

Overall, we may conclude that the membrane of the influenza virus exhibits slow protein and lipid diffusion consistent with raft-like behavior. This is likely to contribute to the structural stability of the viral membrane in response to changes in environmental conditions.

### Protein and Lipid Domain Formation

**Figure 9:**
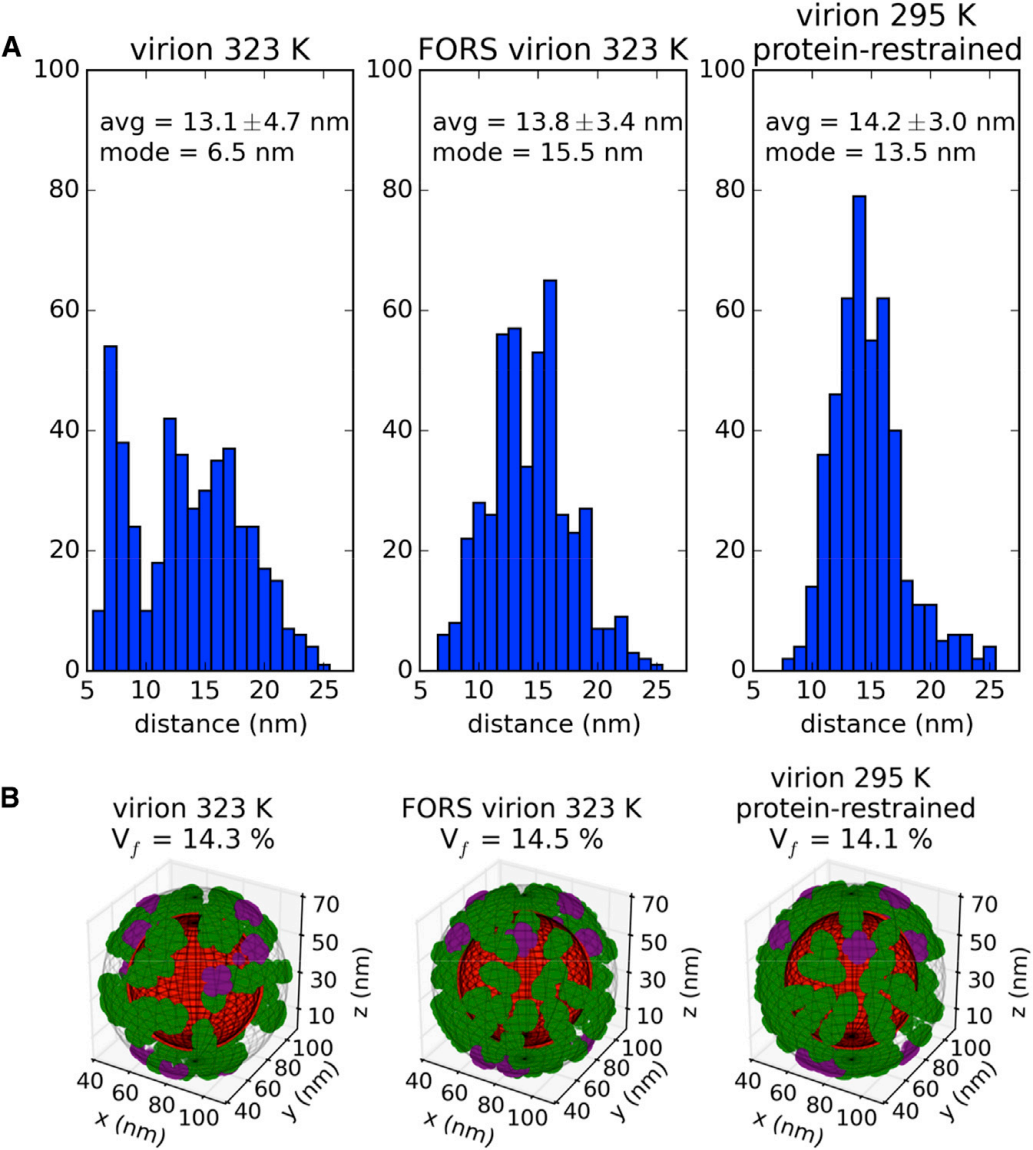
Spatial Disposition of Membrane Proteins (A) Comparison of interprotein distance histograms in final snapshots of simulations (excluding the M2 proton channel). The centroids of each protein were employed and the closest five neighboring proteins were included for 460 distances. (B) Assessment of influenza A spike glycoprotein fractional surface volume in final snapshots of production simulations. The phosphate particles in each virion (red) were used to define the inner boundary of the outer surface layer (contained within a diffuse meshgrid), while the outer boundary was assigned 13 nm farther from the virion centroid (contained within outermost diffuse meshgrid). The coordinates of particles representing the convex hulls of the spike glycoproteins are shown for HA (green) and NA (purple). The percentage of the outer surface layer volume (*V*_f_) occupied by the spike glycoproteins is indicated above each condition. See also Figures S11–S15 and S19.

Given the slow dynamics of the proteins and lipids, it is of interest to examine their spatial organization, as this offers the possibility of linking dynamic structural models through to cryo-tomographic images. The spacing (clustering) of proteins on the surface of the influenza A virion was previously studied by electron cryo-tomography (Harris et al., 2006; Wasilewski et al., 2012), providing useful information about the steric constraints of putative therapeutic binding sites. The average final-snapshot separation between the HA and NA spike proteins in our simulations ranges from 13.1 ± 4.7 nm to 14.2 ± 3.0 nm (Figure 9A; Figure S11), which agrees reasonably (i.e. within errors) with the experimental estimate of 11.2 ± 2.3 nm.

In addition, the simulations allow us to study the evolution of the interprotein spacing (clustering) behavior over time. The most striking change in clustering behavior was observed for the influenza A virion at 323 K (in the absence of the FORS glycolipid) with the average spacing changing over the 5 μs simulation from 14.2 ± 3.0 nm to 13.1 ± 4.7 nm, and with the mode of the distribution dropping drastically from 13.5 nm to 6.5 nm. This is consistent with formal clustering analysis for this simulation condition (Figure S12), where the number of protein clusters increases from 2 to 17. In contrast, in the presence of Forssman glycolipid there is less clustering of the spike proteins, with average final separation of 13.8 ± 3.4 nm, a mode of 15.5 nm, and no substantial evidence of clustering using a formal algorithm. The room temperature (295 K) simulations show similar behavior in the presence or absence of FORS, with final respective average separations of 13.9 ± 3.1 nm and 13.8 ± 3.8 nm. A decrease in interprotein separation to 13.0 ± 4.2 nm was observed for the simulation of the simpler (40% cholesterol) model. Thus, the glycolipid may reduce clustering of the HA and NA proteins, keeping them evenly distributed over the surface of the virion. Our results also suggest that the glycoprotein spikes of a spherical influenza virion may be suitably spaced to be bound by bivalent immunoglobulins G (IgGs), which have flexibly linked Fab domains that can extend 15 nm apart and crosslink adjacent spike glycoproteins (Wasilewski et al., 2012; Wrigley et al., 1983). The spacing between spike glycoproteins in our simulations is also sufficient to allow trivalent binding at the HA stem by the broadly neutralizing FI6 antibody (Wasilewski et al., 2012), which is effective against both group 1 and group 2 influenza A strains (Corti et al., 2011).

We also quantified the fractional volume of the outer virion surface, in a layer 13 nm thick, occupied by the spike proteins (Figure 9B; Figure S13). We obtained values between 14.1% and 15.0%, which are in good agreement with the experimental values reported for three X-31 virions (from 13.5% to 15.5%) (Wasilewski et al., 2012). Visualization of surface layer volumes occupied by proteins also reveals how the presence of the Forssman glycolipid prevents protein clustering, as discussed above.

The clustering of each lipid species was tracked over the course of the simulations, and the results were quantified using a formal clustering algorithm (Figures S14 and S15). The presence of the Forssman glycolipid appears to protect lipid clustering behaviors from temperature sensitivity. The minor populations of DOPE, DOPX, and POPS in the outer leaflet of the virion envelope exhibited six stable clusters each throughout the +FORS simulations at both temperatures. Conversely, in the absence of FORS at 323 K there were as many as 40 clusters at the end of the simulation for the corresponding lipid species.

### Geometric Restrictions on Host Cell Sialic Acid Binding

**Figure 10:**
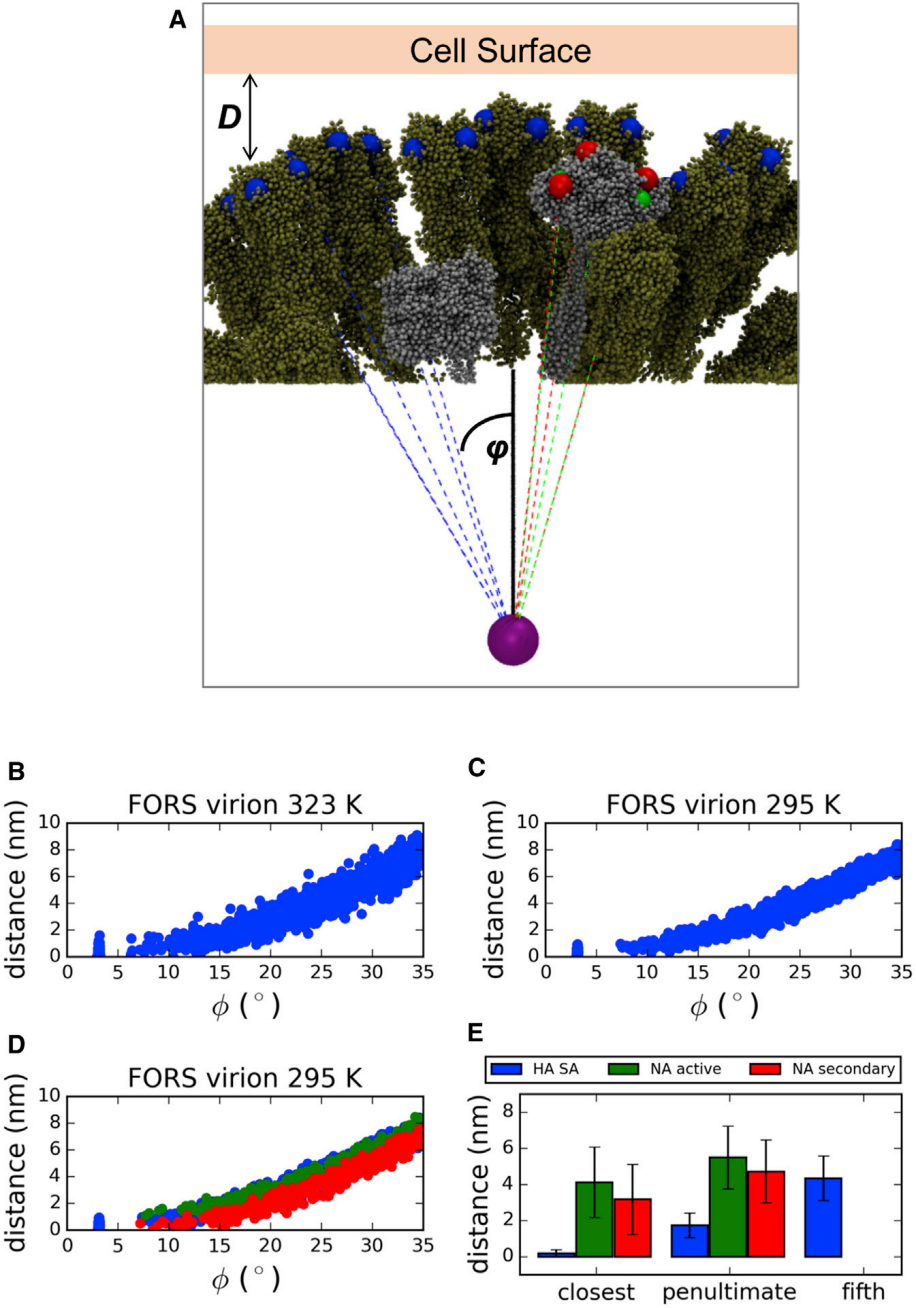
Geometric Constraints on the Binding of HA Trimers and NA Tetramers on the Approximately Spherical Influenza A Virion Surface at the End of the Simulations to Sialic Acid Receptors on an Idealized Host Cell Surface (A) Schematic of SA receptor binding sites (blue) in HA trimers (tan), and of active site (green) and secondary SA receptor binding sites (red) in NA tetramers (silver) for 1/80 randomly chosen influenza A virion-host cell attack orientations with a single reference HA aligned along the +*Z* direction (solid black line). A subset of the binding site vectors (dashed lines) are shown and their angles (φ) are measured relative to the +*Z* reference axis. Putative binding sites are only shown if they fall within φ ± 35° of the virion centroid (purple sphere). (B and C) The cumulative (all 80 attack orientations) HA binding site surface distances and their corresponding angles are plotted for the FORS-inclusive virion at both simulation temperatures. (D and E) The matching results for SA binding sites on NA are also plotted for comparison for the FORS-inclusive virion at 295 K (D), with the closest, penultimate and fifth-closest average protein binding site distances compared (E), where available. For the FORS-inclusive virion at 295 K, the two closest HA trimers have average SA to binding site distances of 0.18 ± 0.20 nm and 1.73 ± 0.68 nm, respectively, which may be compared with closest HA and closest neighbor measured experimentally (Wasilewski et al., 2012). See also Figure S16.

In addition to the average spacing of glycoproteins discussed above, the curvature of the influenza A lipid envelope is also an important determinant of the accessibility of the binding sites on HA and the active and secondary binding sites on NA for the receptors (sialic acid [SA]) on the host cell surface. Given the weak (2–3 mM) in vitro binding affinity of HA for SA receptors (Gamblin and Skehel, 2010; Sauter et al., 1989), it is likely that infection requires multivalent binding (Wasilewski et al., 2012). Given the roughly spherical ultrastructure of our simulated influenza A virions, we assessed the distance of SA binding sites from a planar surface, as a first approximation to the target membrane in a host cell or in vitro assay, averaging across the set of 80 possible attack orientations where a single HA is aligned for direct contact with the surface (Figure 10). We report a range of average SA binding site to surface distances for the closest HA neighbor to the direct-contact HA trimer between 1.2 ± 0.9 nm to 1.7 ± 0.9 nm (with *ϕ* ∼ 14°) (Figure S16). The results are reasonably consistent with previously reported electron cryo-tomography measurements of influenza A virions placing the closest neighbor HA SA sites on average 1.2 nm from a planar surface for *ϕ* = 12° (Wasilewski et al., 2012), especially considering the filamentous morphology of the latter virions. Of course, one would expect a target cell membrane not to be exactly planar. Based on large-scale simulations of bilayers with a lipid composition approximating that of a mammalian cell membrane (Koldsø et al., 2014), one would expect spontaneous fluctuations of the local bilayer geometry to be of a magnitude (in terms of radius of curvature) comparable to that of the dimensions of the virus particle, thus bringing substantially more SA residues within range of the HA molecules. Even if only 10% (n = 8) of the HA trimers in our model engaged host SA residues simultaneously the effective binding constant would depend on their product, yielding a much greater effective affinity. Our results also clearly demonstrate that NA is geometrically capable of competing with HA for interaction with SA beyond the site of direct contact, which may allow for SA cleavage by the NA active site (and virion budding) or additional binding of SA by the secondary site. The distinction between HA and NA was not possible in the above cryo-EM study, and we are now well positioned to examine low-virulence influenza strains where the NA stalk is shortened (Castrucci and Kawaoka, 1993; Durrant and Amaro, 2014).

### Model Limitations

The CG simulation approach is an approximation compared to all-atom simulations (Freddolino et al., 2006). However, it allows for substantially longer simulation times, approaching those accessible by experimental biophysical measurements and permitting assessment of the dynamic behavior of proteins and lipids. Using virion models to assess avidity and multivalent interactions with host cells could benefit from multiscale or hybrid simulations (Ayton and Voth, 2009b; Gonzalez et al., 2013; Wassenaar et al., 2013) that combine both all-atom and CG representations, with atomistic representation utilized in the regions of binding interactions. A possible refinement of the CG simulation methodology would be to scale the system reference pressure by the volume ratio between the simulation box and internal RPO shell, coupling the internal and external pressure independently, as recently described (Louhivuori et al., 2010). However, as discussed below, refinements of the RPO shell model for the virion contents might also be considered.

Another limitation of our virion models is in the representation of the proteins. Experimental structures of the cytoplasmic tail, transmembrane domain, and stem domain of NA are not available, and had to be modeled (Parton, 2011). The model for HA also lacks high-resolution experimental structural information for the TMD, cytoplasmic domain, and a linker between the ectodomain and the TMD (Parton et al., 2013). A portion of the cytoplasmic tail and the acylations are missing from the model of M2 (Parton, 2011), which may influence protein-specific lipid recruitment (Figure S17). Although an X-ray structure for the M1 matrix protein is available (Harris et al., 2006), its ultrastructure remains controversial (Fontana and Steven, 2013).

The RNP core of the virion is not included in our simulations, being replaced by a sphere of suitably restrained particles. The RPO shell does not interact directly with the lipid envelope in our model. In vivo, the inner leaflet of the envelope appears to be lined by the M1 matrix protein, which may influence lipid dynamics. Other approaches could include the use of mobile anions to model the RNA (Larsson et al., 2012). Low-resolution cryo-EM structures of RNPs are available (Zheng and Tao, 2013), and a refined representation of the core of the virion could be included in the future. The current influenza A lipidome is based on virions propagated in canine (MDCK) cells (Gerl et al., 2012), and may need some refinement for consistency with proper human virions (Kijimoto-Ochiai et al., 1981; Yamamoto et al., 2012). It may also be important to include glycans in the CG models of the spike proteins given that, for example, glycosylation of HA may modulate virulence (Medina et al., 2013).

There is some recent evidence that lateral diffusion in CG lipid bilayer simulations may require milliseconds to converge in highly crowded membranes (Javanainen et al., 2013). These authors report reasonable convergence (using a similar force field to ours) of diffusion measured as a Gaussian distribution with *α* generally >0 within 1 μs in a system with 10-fold greater protein/lipid ratio than in the virion model. Averaging MSD values of multiple subtrajectories has previously been used to improve convergence of diffusion in CG lipid bilayer simulations (Goose and Sansom, 2013), and we have employed a similar strategy with the virions.

### Conclusions

We have performed a number of CG resolution simulations of complete influenza virion membranes under a variety of conditions. Room temperature simulations with Forssman glycolipid present result in reduced mobility of bilayer species. The presence of protein restraints (to mimic interaction of spike proteins with M1 matrix) also restricts lipid mobility. We anticipate that reduced mobility in the virion membrane is likely to confer physical robustness to changes in environmental conditions. Our simulations also suggest that the viral membrane proteins do not promiscuously “clump together” and are competent for IgG antibody binding. There is a substantial public health interest in understanding the biophysical properties of the influenza A virion. Worldwide, influenza causes more than 250,000 deaths annually (http://www.who.int/mediacentre/factsheets/2003/fs211/en/), and the virion has been projected to persist in distilled water for longer than 3 years (Stallknecht et al., 1990). Furthermore, the virus can survive for extended periods (>12 days) in seawater (Mihai et al., 2011). Thus, we have produced a computational platform for probing the structural and biophysical stability of the virion in water. There are several other pleomorphic enveloped viruses that affect human health, including rubella virus, HIV, hantavirus, Ebola virus, rabies virus, and hepatitis C. The simulation and modeling studies described here may provide transferable approaches to studies of these other viruses.

## Experimental Procedures

### Simulation Details

All vesicle/virion simulations were performed using GROMACS 4.5.5 (Hess et al., 2008) (http://www.gromacs.org) and the MARTINI 2.1 force field (Marrink et al., 2007; Monticelli et al., 2008), with all simulation times reported without correction factors. Default ionization states were assumed for amino acids and lipid headgroups. The force field was modified to include a “restrained shell particle” (RPO) which was attractive to water, while super-repulsive to other particles. RPO particles were always position-restrained in each dimension with a force constant of 10^3^ kJ/mol/nm^2^. The following lipid models were also included: ether-linked dioleoylphosphatidylethanolamine (DOPX), in which we modeled the ether linkage at the C1 position by changing particle 4 in DOPE from type Na to N0; and hydroxylated sphingomyelin (PPCH), in which we modeled the hydroxyl group of sphingomyelin (PPCS) particle 5 by changing this from particle type C1 to Nda. The Forssman glycolipid was parameterized in two stages. First, the ceramide backbone was based on the matching particles provided for sphingomyelin in the MARTINI force field. Second, the headgroup of the Forssman glycolipid comprised the monosaccharides glucose, galactose, and *N*-acetylgalactosamine, with the glycosidic linkages as follows: GalNac-α(1-3)–GalNac-β(1-3)–Gal-α(1-4)–Gal-β(1-4)–Glc-β(1-1)–Cer. The initial monosaccharide parameters were based on the sweet MARTINI force field (Lopez et al., 2009) and previous in-house parameterization of the glycolipid GM3 (D.S., personal communication), and were refined by comparison with all-atom simulations using the GLYCAM force field (Kirschner et al., 2008). Further details of the CG Forssman glycolipid are provided in the Supplemental Information.

The HA, NA, and M2 proteins were modeled as described previously (Parton, 2011), and as described in more detail in the Supplemental Information (Figure S18). In brief, the model of HA was based on the X-ray structure Protein Data Bank (PDB) code 1MQM. This X-ray structure does not include the TM domain or the cytoplasmic domains, or a short linker between the ectodomain and TM domain. This missing region was modeled as an α helix. Palmitoyl chains were added to residues Cys555, Cys562, and Cys565 of the TM domain. The cytoplasmic domain was treated as unstructured. The NA stalk domain was modeled as a polyalanine coiled coil (based on a tetrameric coiled coil motif from the GCN4 leucine zipper protein, PDB code 1GCL, with its length (∼10 nm) matched to cryo-EM images of the protein. The CG model of the M2 proton channel was derived directly from an NMR structure (PDB code 2RLF) comprising residues 18–60 of the M2 protein and including 15 residues of the C-terminal cytoplasmic tail as well as the α-helical TM domain.

Simulations employed 10 fs time steps, and coordinates were saved every 10^4^ steps (every 0.1 ns). Coulomb and van der Waals interactions were respectively shifted off between 0.0 and 1.2 nm and 0.9 and 1.2 nm. Acylated proteins, lipids, RPO particles, and solvent were separately temperature coupled using the Berendsen algorithm (Berendsen et al., 1984) with a 1.0 ps time constant. Isotropic pressure coupling was performed using the Berendsen algorithm with a 1.1 ps time constant, 1 × 10^−6^/bar compressibility, and a 1.0 bar reference pressure. In the case where all 107 viral membrane proteins were restrained, each of the proteins was subject to center of mass pulling (in all three dimensions) using an umbrella potential in GROMACS. The pull force (harmonic force constant, *k* = 10^5^ kJ/mol/nm^2^) was exerted in the direction of an absolute reference point at the origin, with initial pull vector set to (0,0,0) and pull rate set to 0 nm/ps (immobilized reference) for each protein. Forces and center of mass values from the pull code were written every 10^4^ steps (every 0.1 ns). All systems were neutralized with Na^+^ ions (Figure S3).

## Author Contributions

T.R. benchmarked, set up, performed, and analyzed simulations. D.P. developed initial viral models and simulations. D.S. provided glycolipid parameters, and E.J. and P.W.F. provided additional methodologies. T.R. and M.C. produced visualizations. T.R., M.B., and M.S.P.S. designed the research project. T.R., P.W.F., and M.S.P.S. wrote the manuscript.

## Supplemental Information

Document S1. Figures S1–S19 and Supplemental Computational Procedures

Document S2. virion_295K_final_snapshot.zip

Document S3. virion_295K_protein_restrained_final_snapshot.zip

Document S4. virion_323K_final_snapshot.zip

Document S5. virion_FORS_295K_final_snapshot.zip

Document S6. virion_FORS_323K_final_snapshot.zip

Document S7. Article plus Supplemental Information
