# Supplementary material for: Nothing to Sneeze At: A Dynamic and Integrative Computational Model of an Influenza A Virion
Source: Structure. 2015 Mar 3;23(3):584–97. doi: 10.1016/j.str.2014.12.019 (PMC4353694; doi:10.1016/j.str.2014.12.019)
Supplement: Document S1. Figures S1–S19 and Supplemental Computational Procedures [file mmc1.pdf]

Structure, Volume *23*

Supplemental Information

Nothing to Sneeze At: A Dynamic and Integrative Computational Model of an Influenza A Virion

Tyler Reddy, David Shorthouse, Daniel L. Parton, Elizabeth Jefferys, Philip W. Fowler, Matthieu Chavent, Marc Baaden, and Mark S.P. Sansom

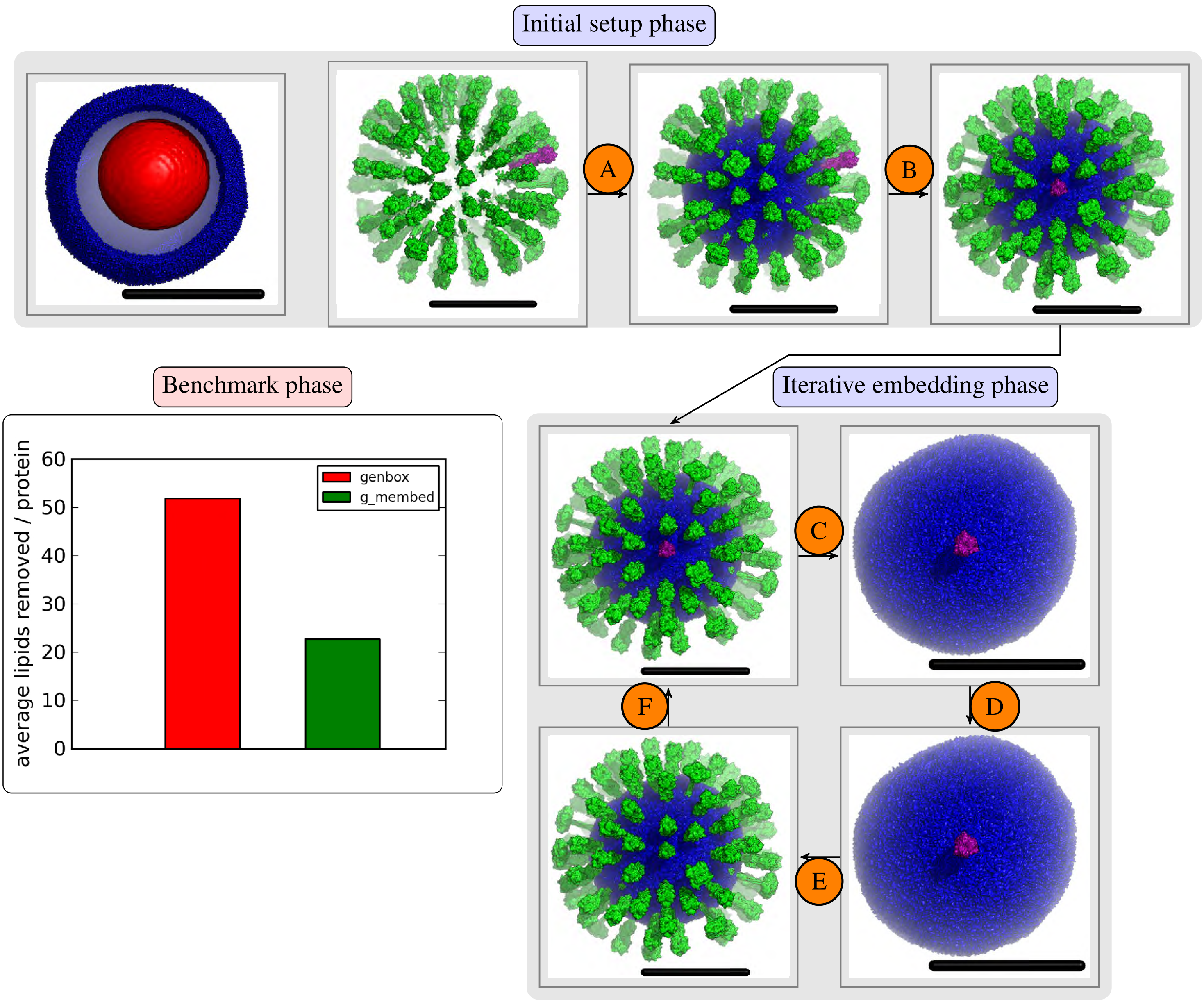

Figure S1: (Related to Figure 1) Procedure for embedding viral proteins into equilibrated vesicle. Lipids are shown in blue and RPO core in red, with a 40 nm scale bar shown in black. The protein membrane-embedding candidate (an HA trimer) is shown in purple and the other proteins in green. In part (A), the equilibrated vesicle and RPO coordinates are combined with the protein coordinates directly by superposition. In part (B), the system is translated to place the centroid of the RPO core at the origin and aligned such that the principal axis of the protein-embedding candidate is along the Z-axis. In part (C), only the protein embedding candidate and already-embedded proteins are retained prior to g_membed, with all other superposed proteins stripped from the system. In part (D), the g_membed code is used to embed the candidate protein by shrinking to 0.1 fractional xy size and expanding to full size in 1000 steps (0.8 nm probe radius, NVT). In part (E), stripped protein coordinates are reintegrated to their superposed positions. Finally, in part (F), the system is rotated to place the next protein membrane-embedding candidate along the Z-axis, unless the loop has completed and all proteins have been embedded. Benchmarking results compare the number of lipids removed per protein using this approach and a crude solvation-based approach reported previously (Parton, 2011).

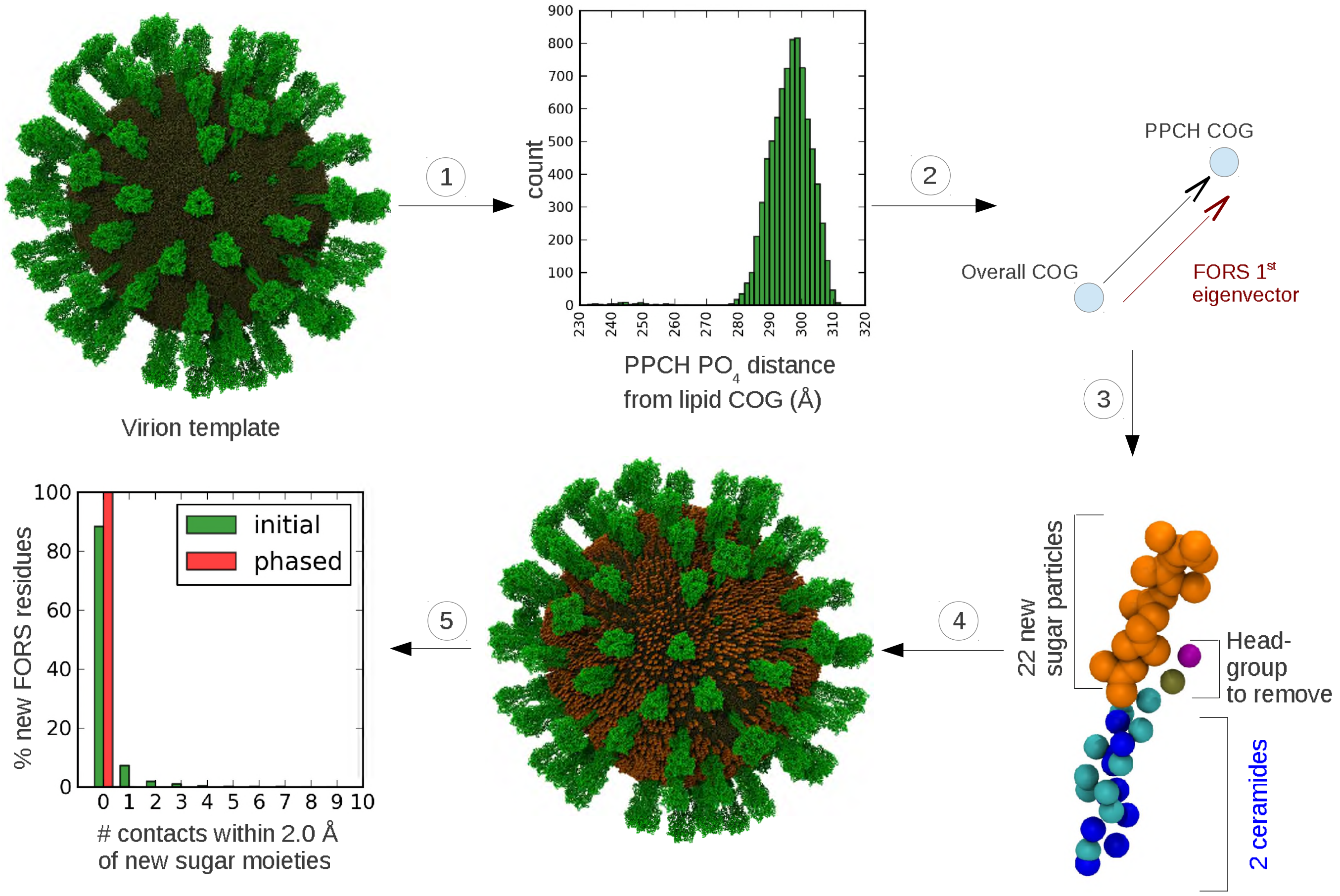

Figure S2: (Related to Figure 1) Producing an influenza virion with Forssman glycolipid by computational mutagenesis. The initial configuration consists of a virion template with 7931 sphingolipids (hydroxylated sphingomyelin residues, PPCH). An assessment of PPCH headgroup distance from the lipid centroid of the system allows identification of the large majority of PPCH residues residing in the outer leaflet (*step 1*). A random subset (68 % to match lipidome (Gerl et al., 2012)) of outer leaflet PPCH residues (> 280 Å threshold) was used to generate vectors connecting the overall lipid centroid of the system to their lipid residue centroid. The first eigenvector of a new FORS glycolipid molecule was aligned to each of the latter vectors to ensure that substitution-candidate FORS glycolipids preserved the bilayer orientation of the original PPCH residues (*step 2*). The new FORS molecules were then translated such that the centroid of their last 10 particles matched the centroid of the last 10 particles of the original PPCH (*step 3*). With an approximate overlap of ceramide tails for the original and substitution-candidate sphingolipids, the original ceramide was preserved (to avert introduction of steric conflicts) while the PPCH headgroup was replaced with the FORS glycan (*step 4*). The 5337 new sugar moieties (*orange*) initially exhibit steric conflicts within a 2.0 Å threshold and were therefore progressively incorporated to the system by adjustment of the GROMACS soft core potential, which resolved steric conflicts for 99.96 % of the FORS sugar moieties (*step 5*).

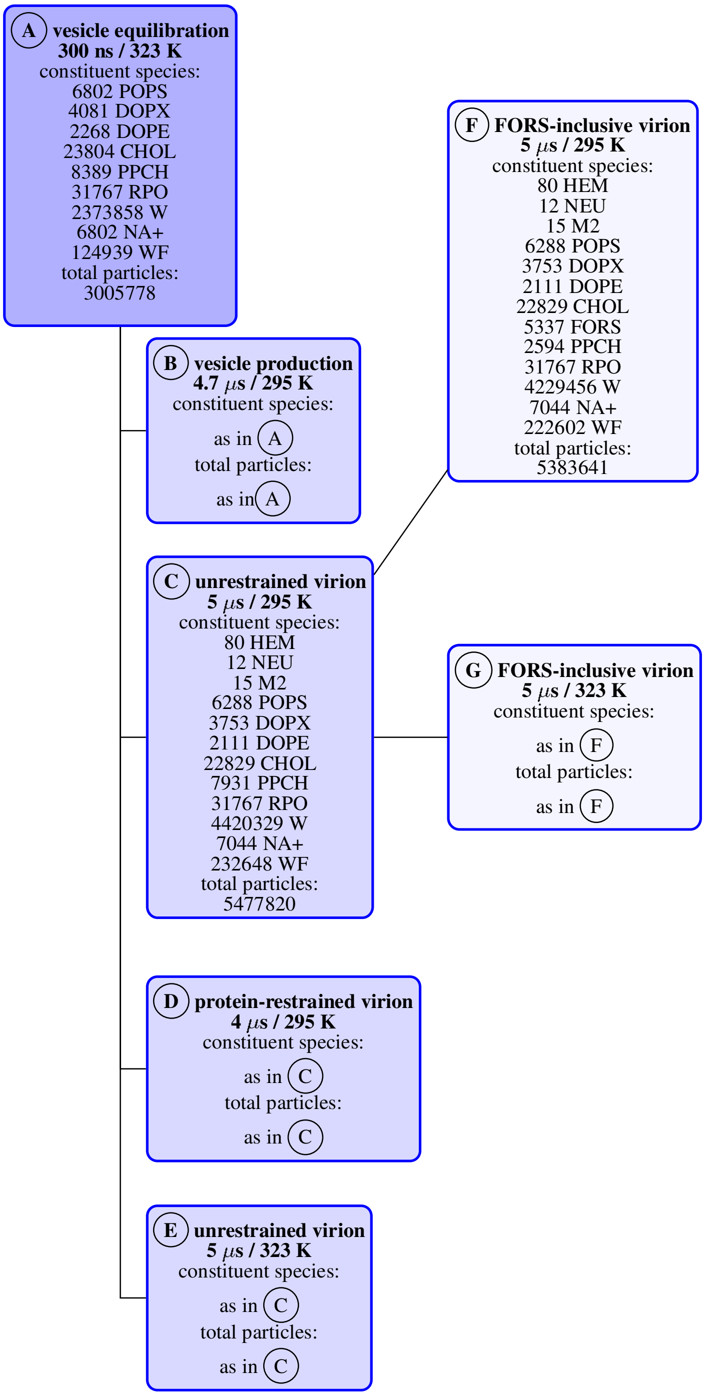

Figure S3: (Related to Figures 1,3) Flow chart summary of the construction and interdependence of vesicle and virion simulation constructs of influenza A. Substantially more detailed than version in the manuscript proper.

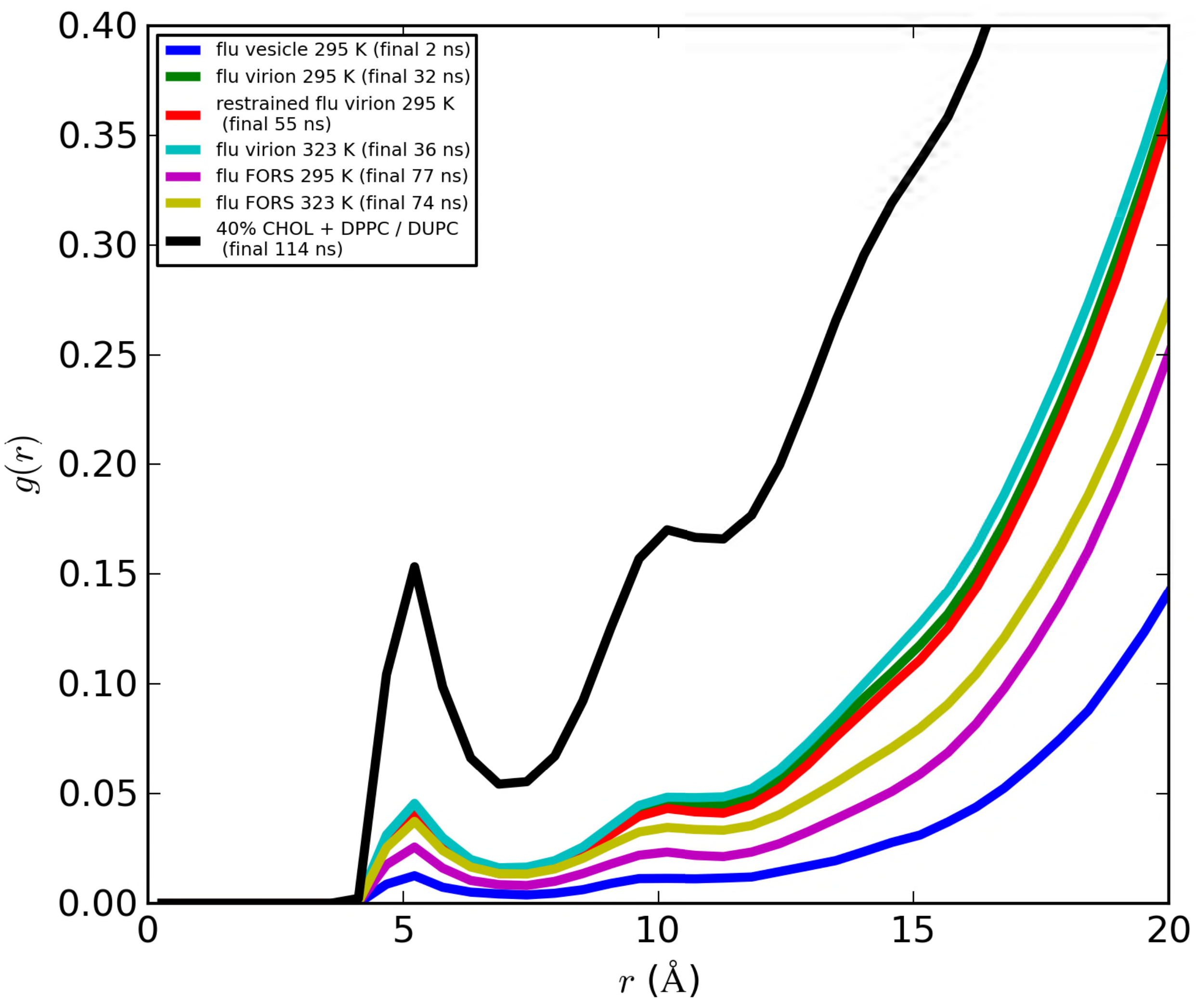

Figure S4: (Related to Figure 2) RDFs between influenza lipid tail particles and solvent particles.

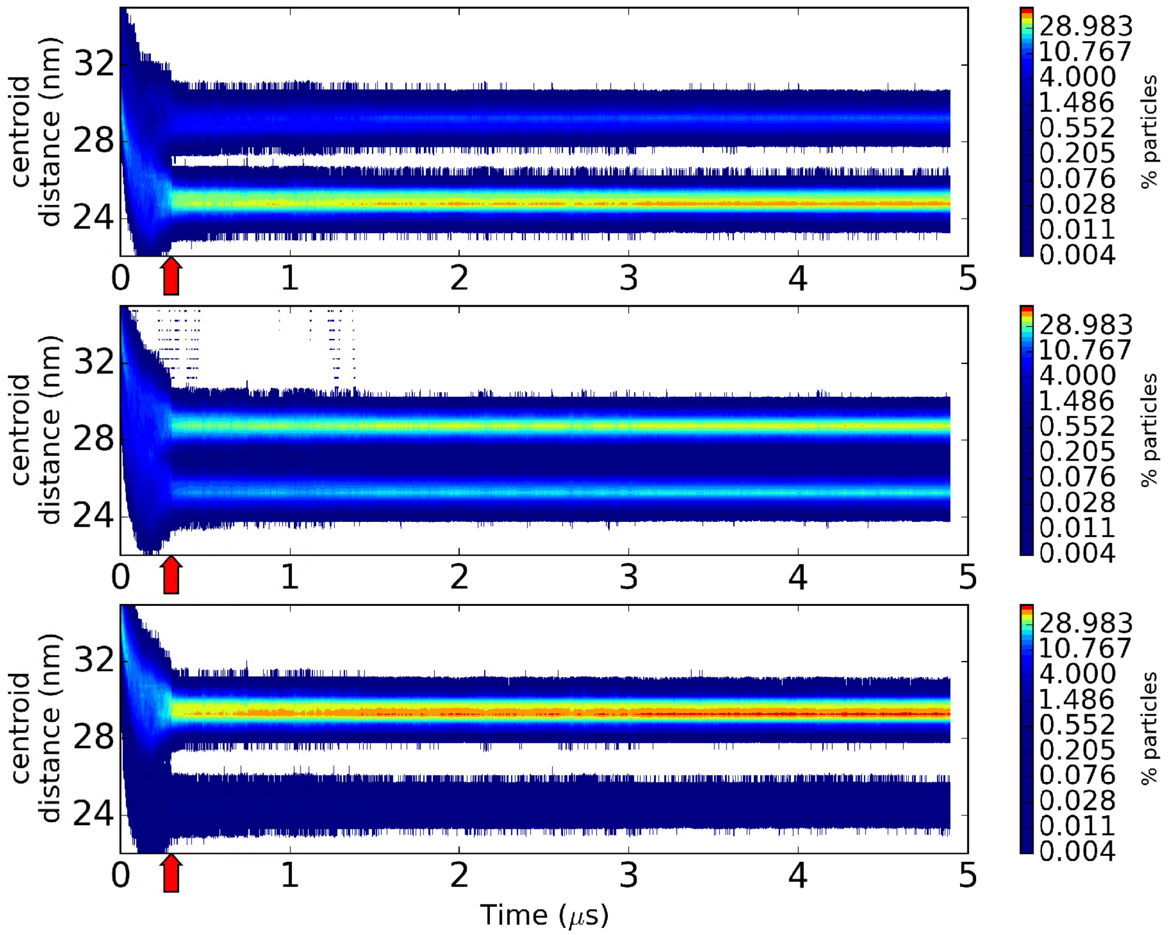

Figure S5: (Related to Figure 2) Tracking the leaflet distribution of viral lipids in a vesicle. Contour plots of the distance between lipid species and the vesicle centroid are shown for representative inner leaflet species (POPS, *top*), central species (CHOL, *middle*), and outer leaflet species (PPCH, *bottom*). Phosphate particles were used for POPS and PPCH, while the ROH group was used for calculations with CHOL. The red arrows indicate the time (0.3 μs) where the temperature was transitioned from 323 K to 295 K.

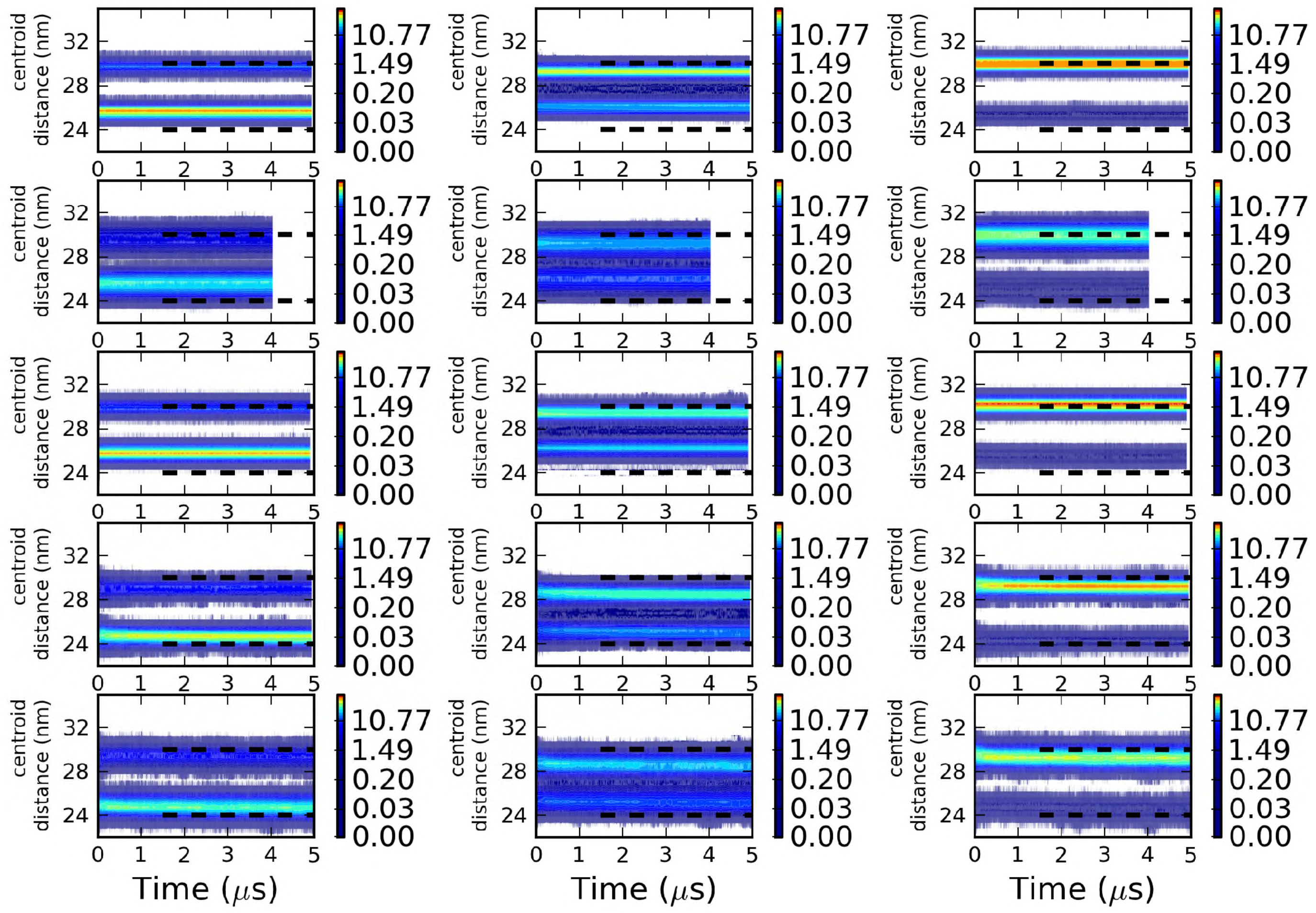

Figure S6: (Related to Figure 5) Comparing the leaflet distribution of lipids in a set of virions. Contour plots of the distance between the lipid species and the virion lipid centroid are shown for representative inner leaflet species (POPS, *left*), central species (CHOL, *middle*), and outer leaflet species (PPCH, *right*), with values categorized according to the % particles at a particular distance. The different virion conditions include unrestrained proteins at 295 K (*first row*), restrained proteins at 295 K (*second row*), unrestrained proteins at 323 K (*third row*), and unrestrained proteins with FORS glycolipid at 295 K (*fourth row*) or 323 K (*fifth row*).

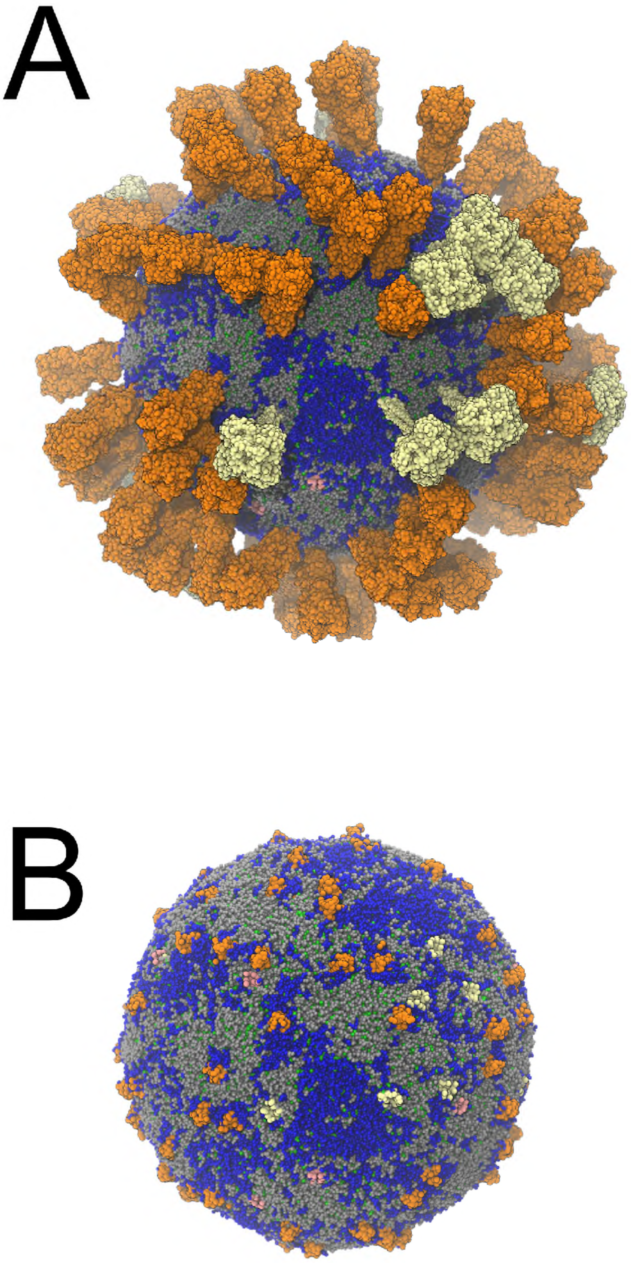

Figure S7: Final snapshots of the $1.8\ \mu$s virion production simulation (lipid envelope composition: 40% CHOL, 36% DPPC, 24% DUPC) with (A) or without (B) viral protein ectodomains at 310 K. Species are coloured by the following scheme: HA (*orange*), NA (*white*), M2 (*pink*), DPPC (*grey*), DUPC (*dark blue*), CHOL (*green*). Solvent particles have been excluded for clarity.

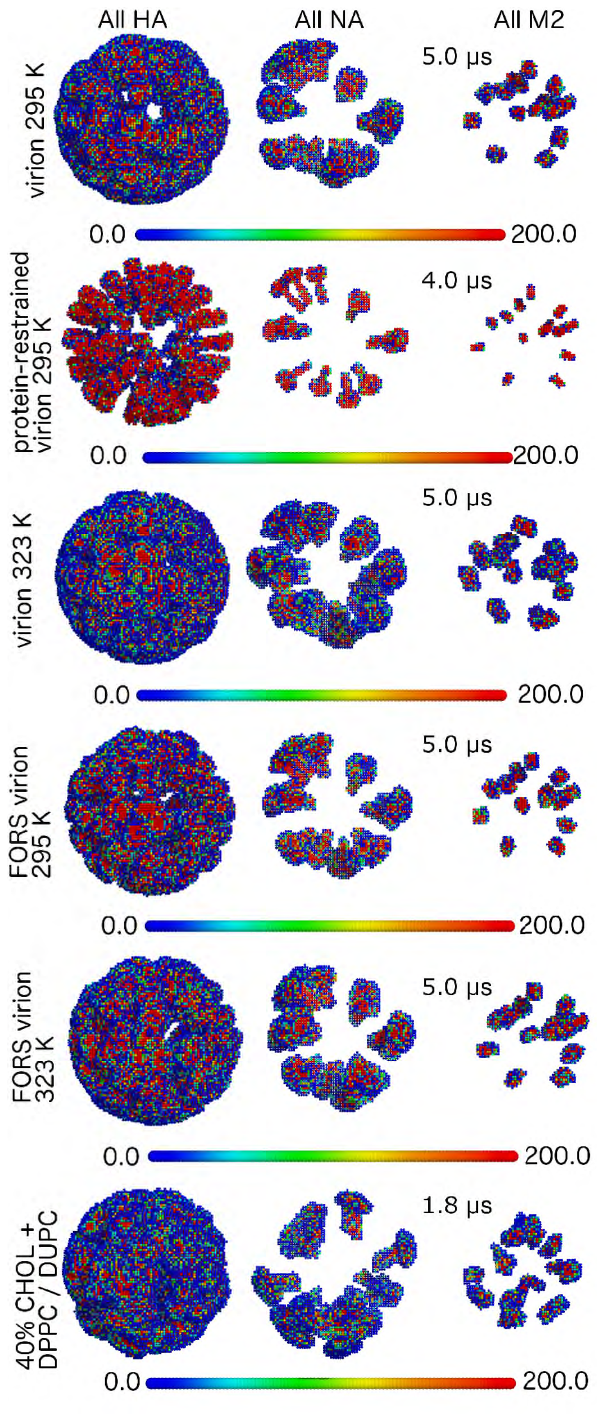

Figure S8: (Related to Figure 6) Assessment of virion protein mobility using contour maps on a linear scale of 0 to 200 counts in 12.5 Å side cubic bins. The counts are for the presence of protein particles accumulated over the total set of frames in a given replicate, with 10000 frames per μs.

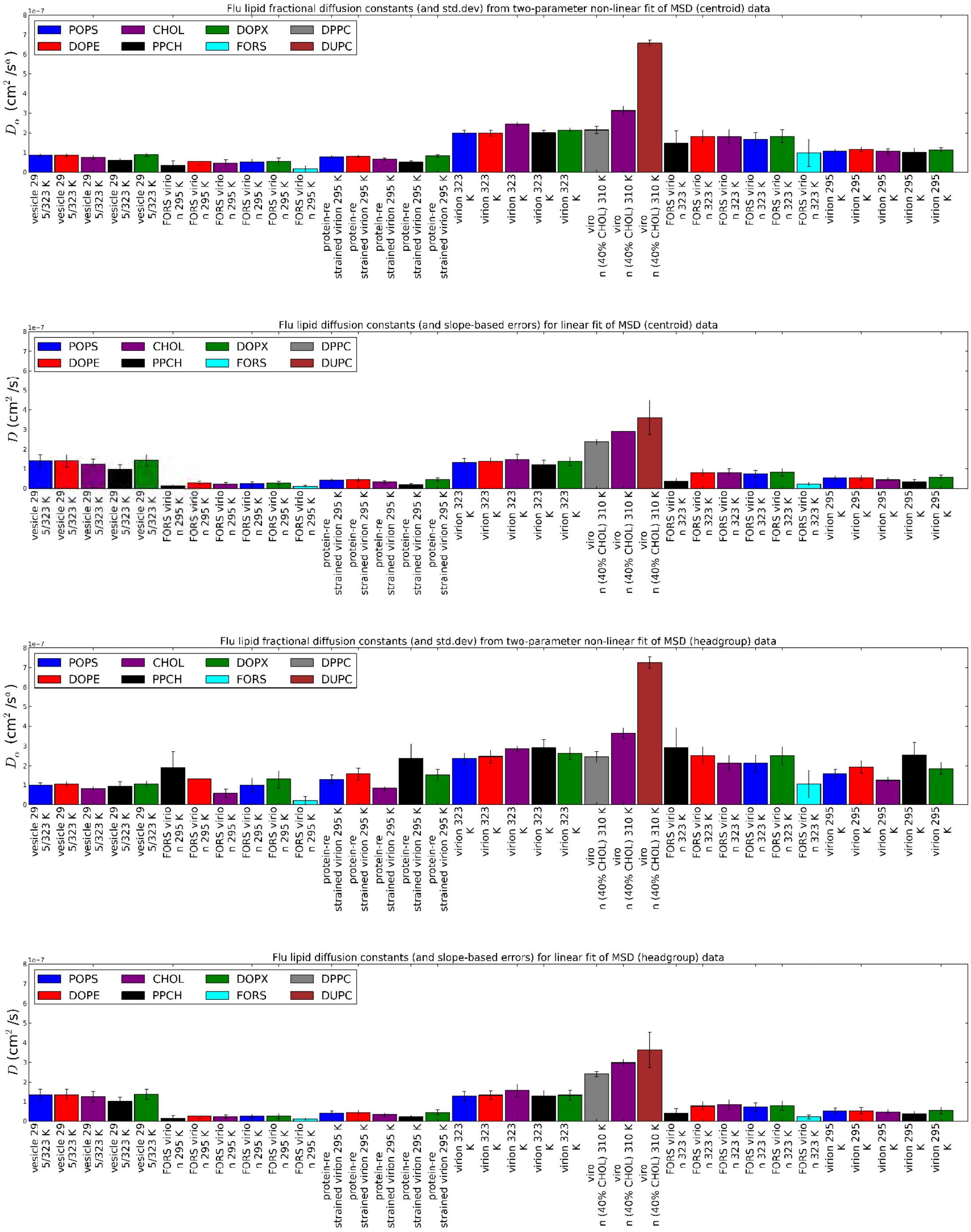

Figure S9: (Related to Figure 7) Comparison of diffusion constants for all lipid species in all influenza A simulation conditions. The constants were calculated based on the centroid of lipid residues (*top*) or on their headgroup particles only (*bottom*), using either two-parameter non-linear fits to the MSD vs time data or using linear fits. Standard deviations are shown for the former fitting procedure while the difference in the slopes of the two halves of the data are used to estimate errors in the latter. 1.8 μs of data were available for the 40% CHOL condition, and approx. 4 μs were available in the presence of protein mobility restraints, otherwise 5 μs trajectories were used.

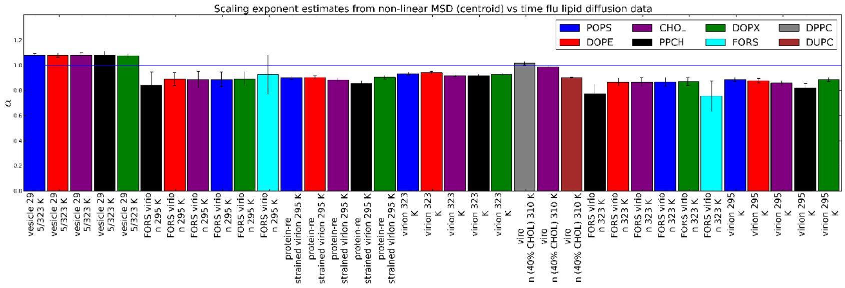

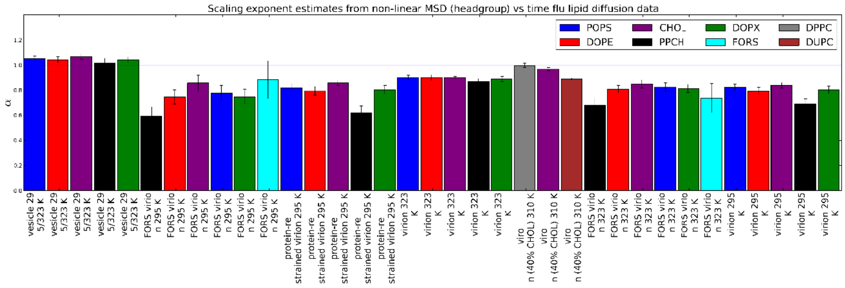

Figure S10: (Related to Figure 8) Comparison of diffusion scaling exponents (α) for all lipid species in all influenza A simulation conditions. α values were calculated based on the centroid of the lipid residues (*top*) or on their headgroup particles only (*bottom*). The non-linear two-parameter fit analysis produces α directly as a fitting parameter. It is possible to estimate the uncertainty as the square root of the diagonal of the covariance matrix from the non-linear fit, producing the standard deviations shown here. A guide line (*blue*) is shown for true random walk diffusion ($\alpha = 1$).

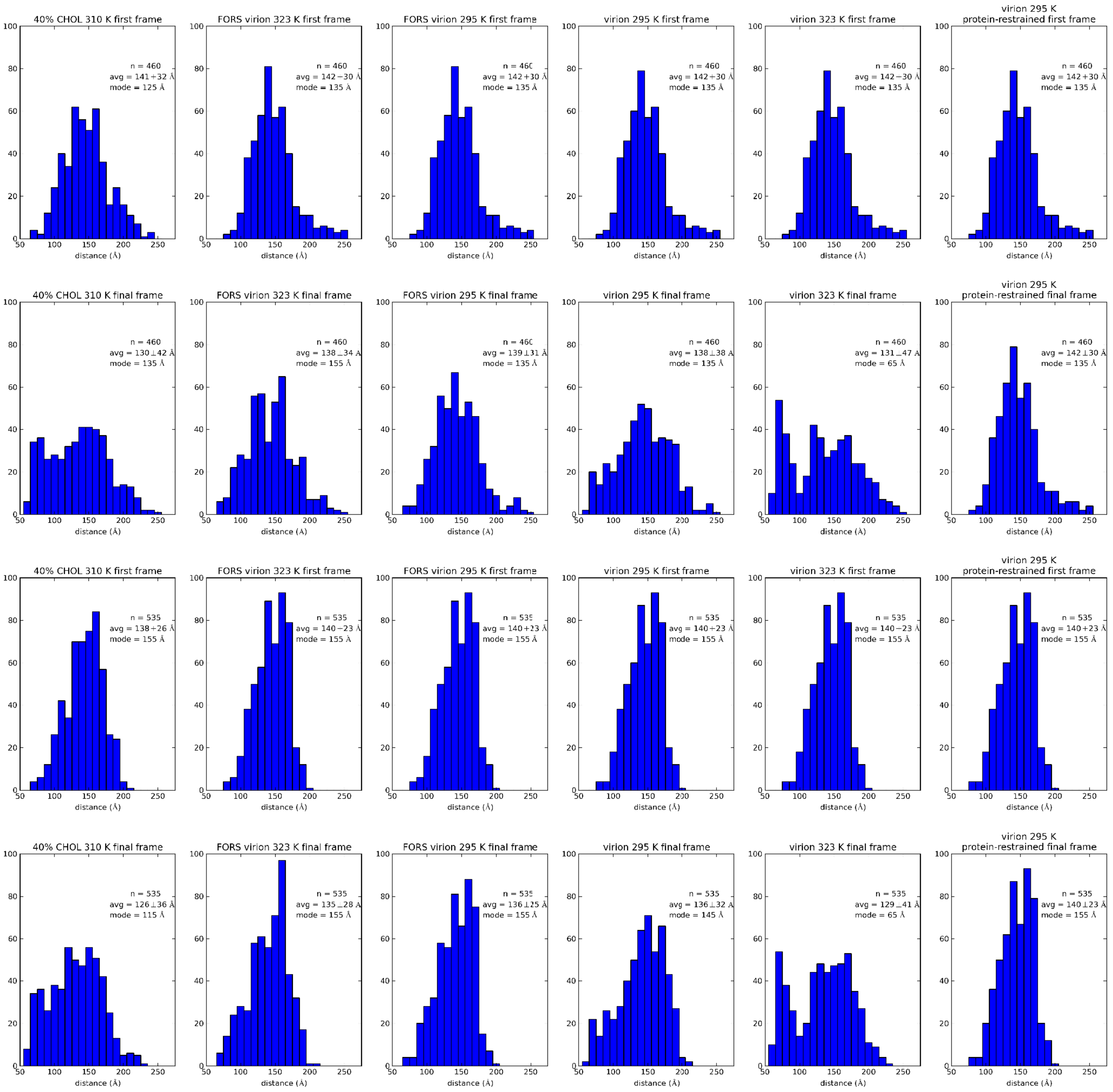

Figure S11: (Related to Figure 9) Comparison of interprotein distance histograms in first and final snapshots of simulations when excluding (*top 2 rows*) or including (*bottom 2 rows*) the M2 proton channel. The centroids of each protein were employed and the top five contacts per protein were included for 460 distances (no M2) or 535 distances (with M2).

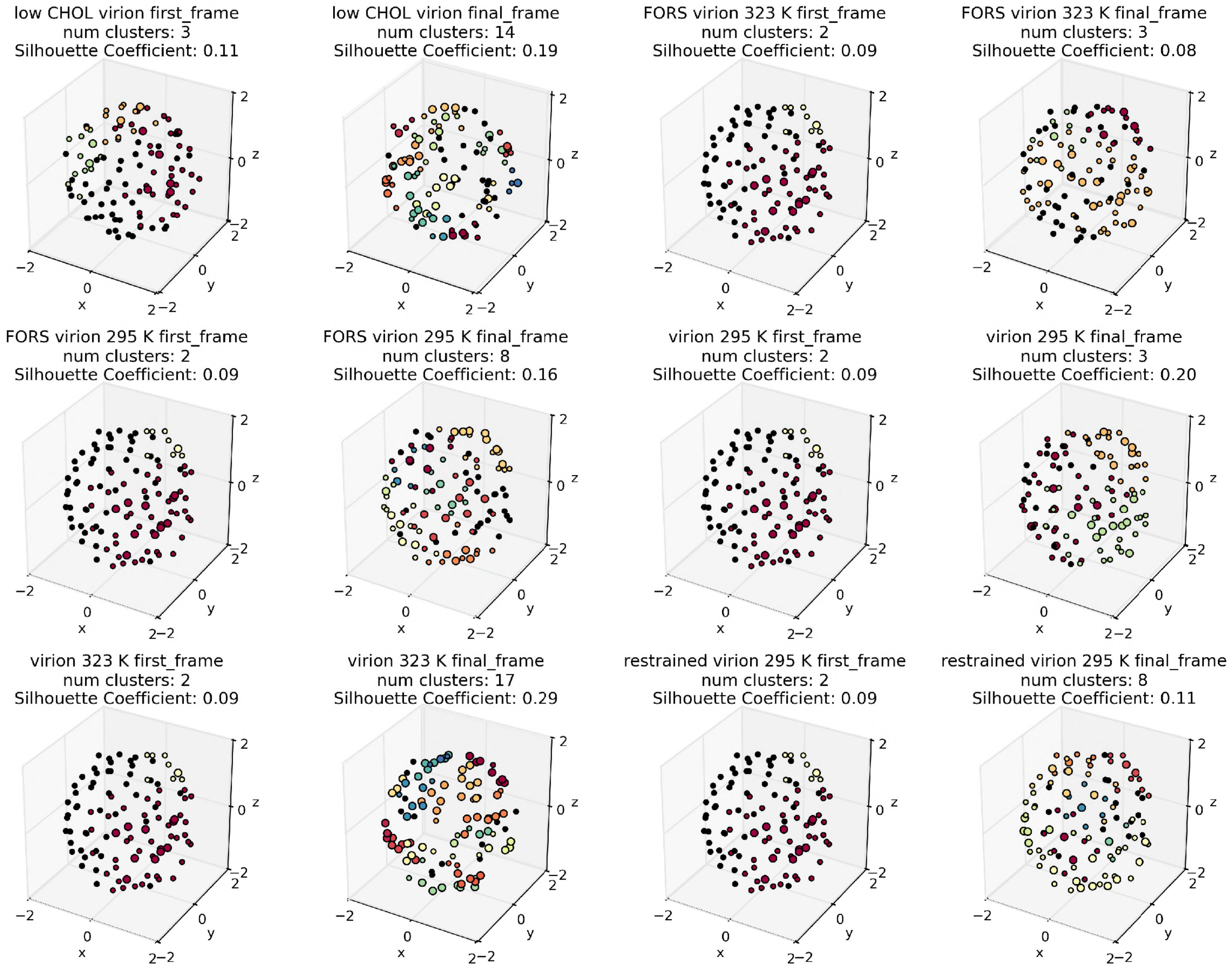

Figure S12: (Related to Figure 9) Clustering of proteins at start and end of influenza A virion simulations using the DBSCAN algorithm. Normalized protein centroid coordinates are coloured based on cluster assignment, with large points representing core samples, small points for edge values, and black dots for coordinates assigned as noise. Silhouette coefficient values closer to 1.0 provide higher confidence in the quality of the cluster assignments.

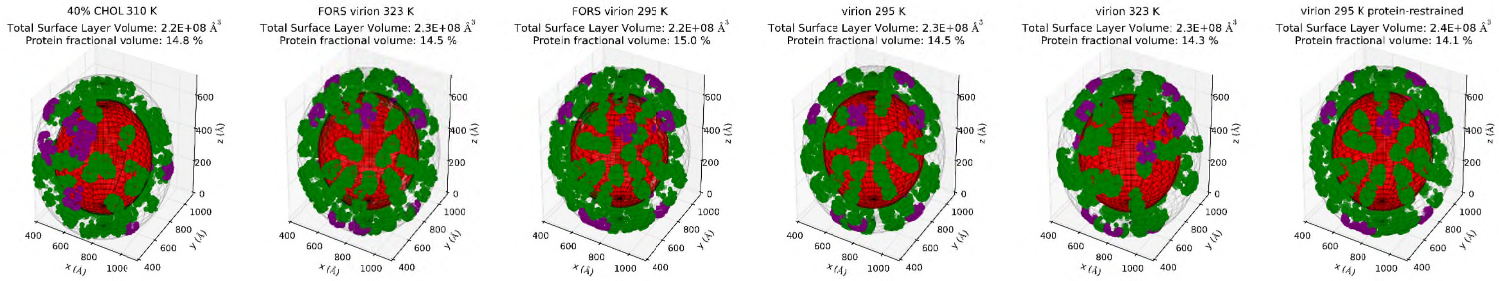

Figure S13: (Related to Figure 9) Assessment of influenza A spike glycoprotein fractional surface volume in final snapshots of production simulations. The phosphate particles in each virion (*red*) were used to define the inner boundary of the outer surface layer (contained within a diffuse meshgrid), while the outer boundary was assigned 13 nm farther from the virion centroid (contained within outermost diffuse meshgrid). The coordinates of particles representing the convex hulls of the spike glycoproteins are shown for HA (*green*) and NA (*purple*). The % of the outer surface layer volume occupied by the spike glycoproteins is indicated above each condition.

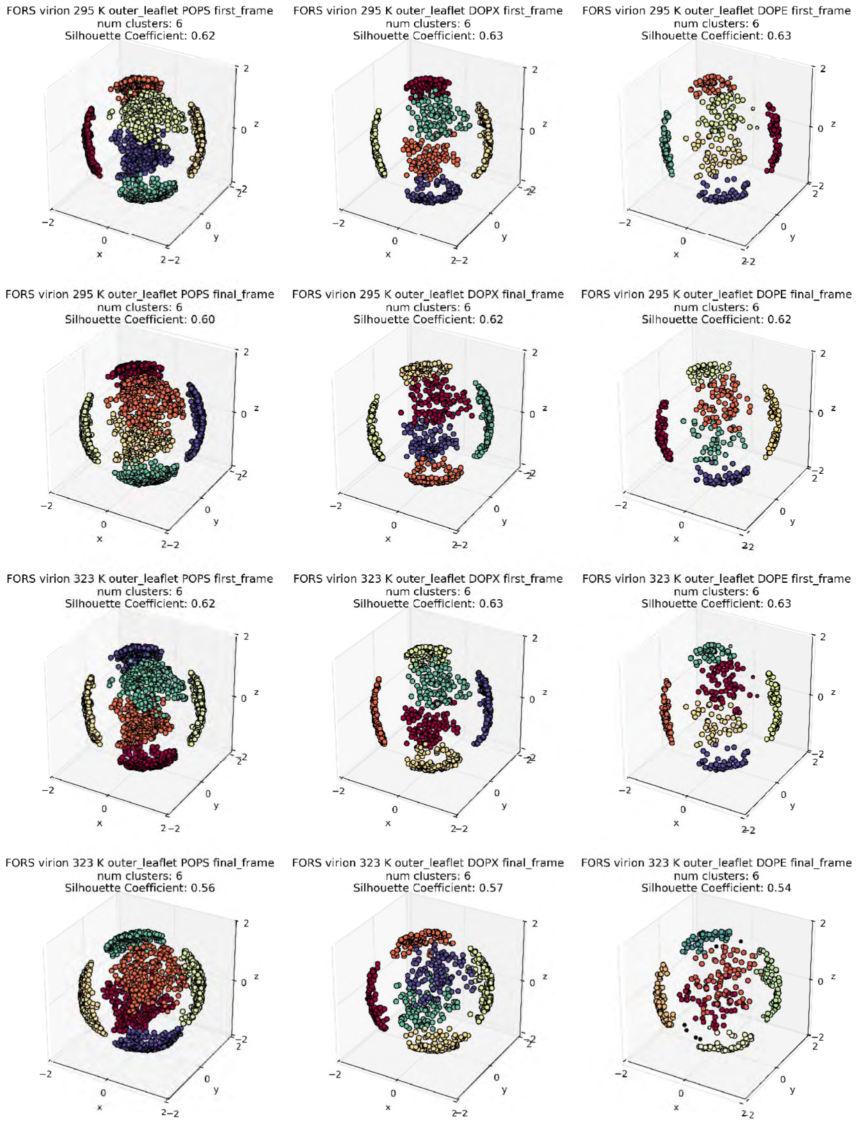

Figure S14: (Related to Figure 9) Clustering of minor outer leaflet lipid species in first and final snapshots of Forssman glycolipid-inclusive virion simulations using DBSCAN algorithm at two temperatures. Plot details as described in Figure S12.

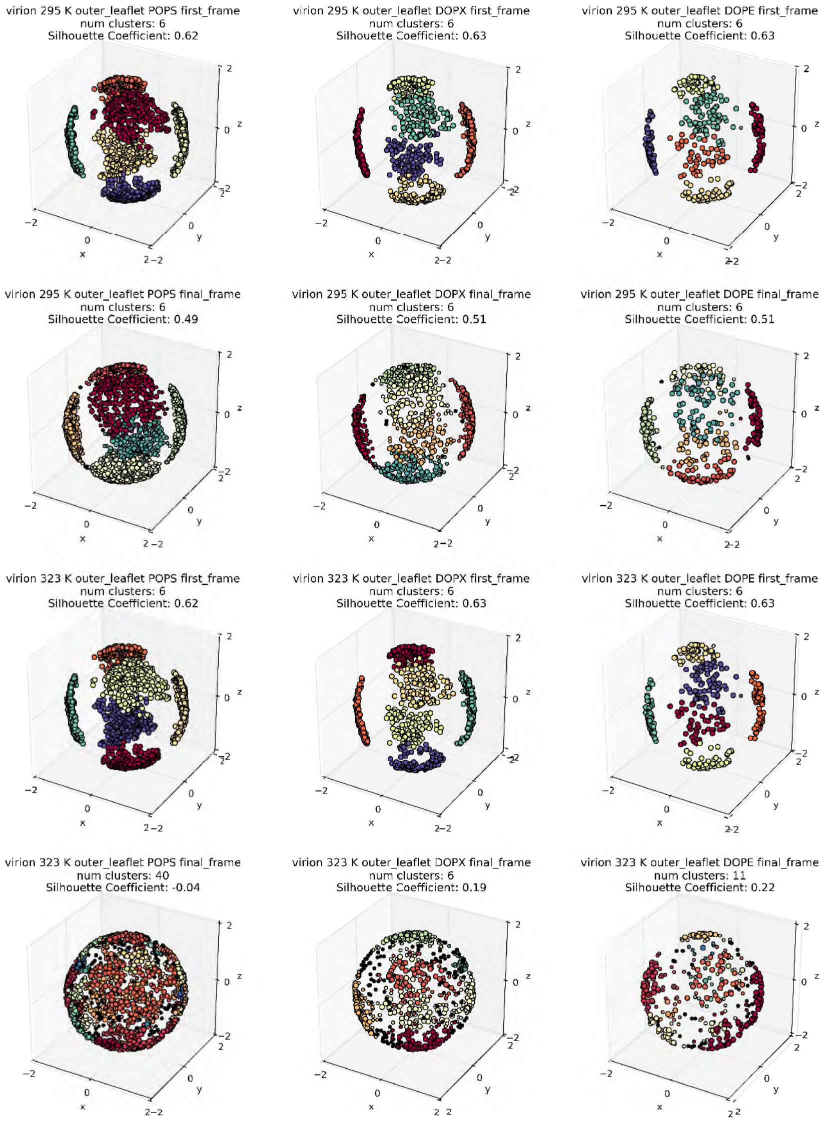

Figure S15: (Related to Figure 9) Clustering of minor outer leaflet lipid species in first and final snapshots of virion simulations lacking the Forssman glycolipid using DBSCAN algorithm at two temperatures. Plot details as described in Figure S12.

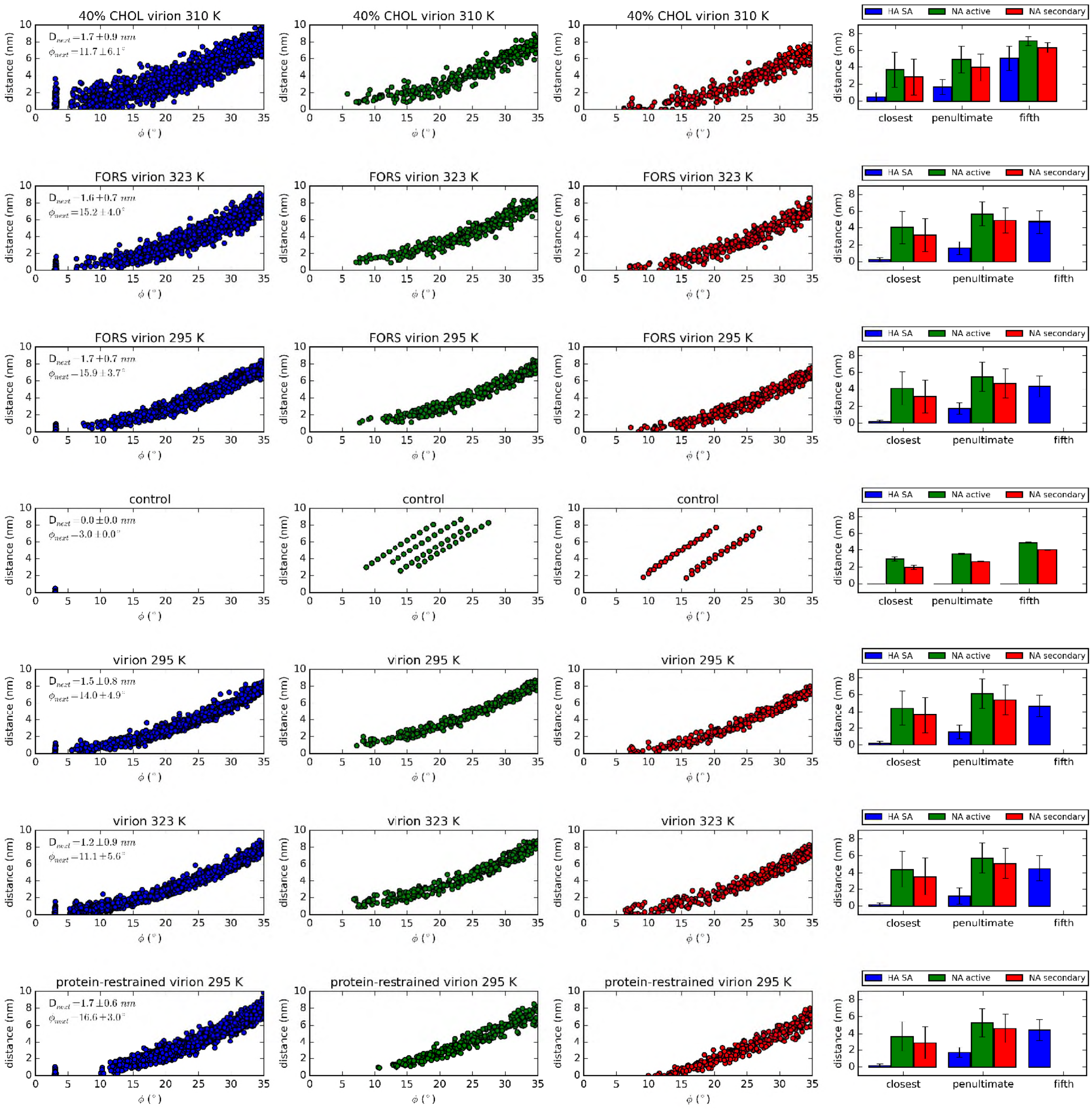

Figure S16: (Related to Figure 10) Geometric constraints on host cell sialic acid binding to HA trimers and NA tetramers on the curved influenza A virion surface at the end of the simulations. The definitions of the ϕ and distance parameters are as demonstrated in Figure 10. For all simulation conditions, the relationship between surface distance and ϕ is plotted for the HA SA binding sites (*blue*), the NA active sites (*green*) and the NA secondary sites (*red*). The data is accumulated over all eighty possible virion-host cell attack orientations where a single HA trimer is aligned along the +Z axis. The bar charts (*fourth column*) summarize the overall average surface distance for the SA binding sites on the closest, penultimate and fifth closest proteins, where available, with their standard deviations. The inset distance and ϕ values for the HA SA site data (*first column*) correspond to the penultimate (second closest) HA trimer, for comparison with experimental values for the closest neighbour HA (Wasilewski et al., 2012). A control condition (*fourth row*) with linear-spaced NA tetramers and superposed HA trimers was used to verify the algorithm.

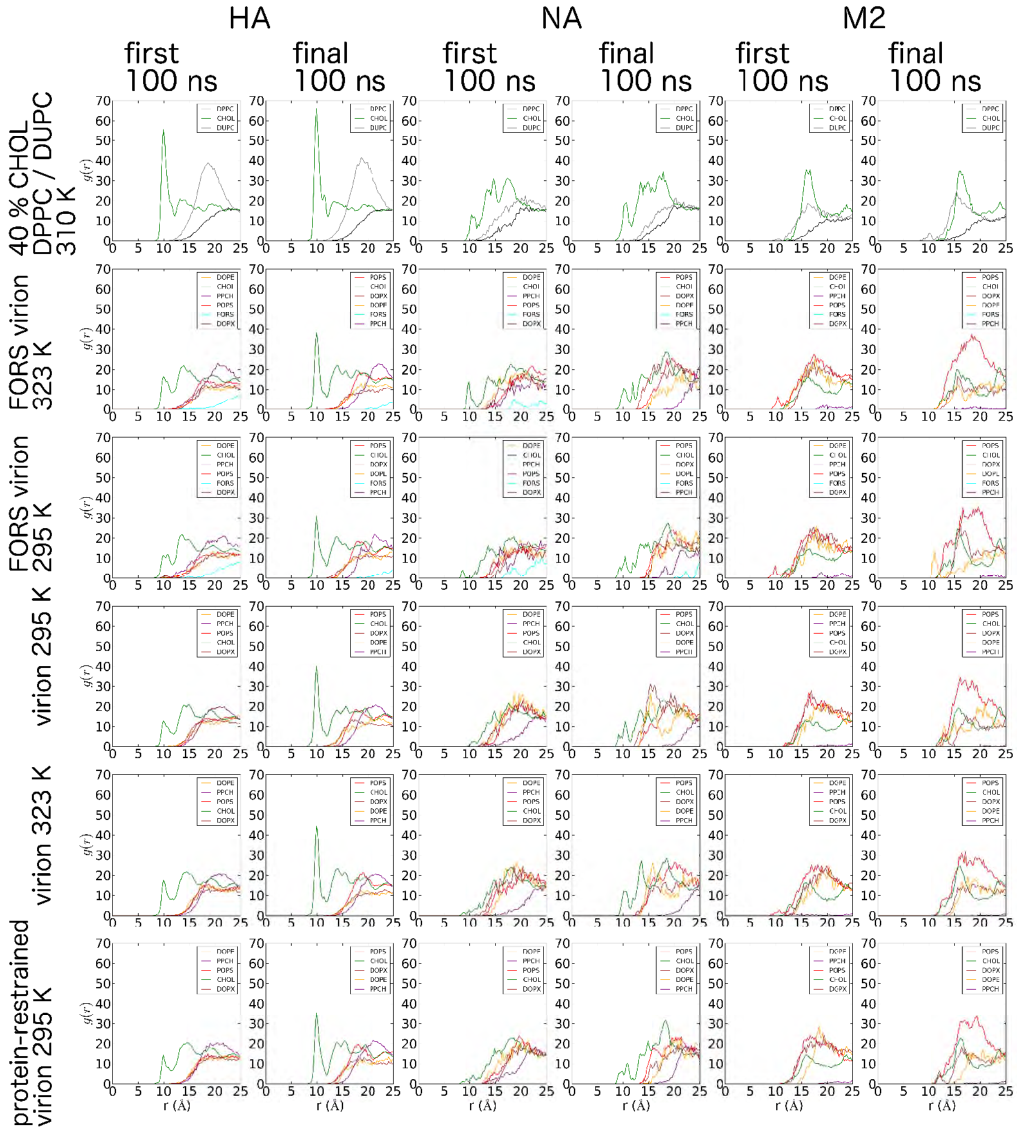

Figure S17: (Related to Figure 5) Radial distribution functions between influenza A protein types (HA, NA, M2) and lipid species compared between first 100 ns and final 100 ns simulation windows for all simulation conditions. The calculations were performed between the TMD region centroids of the biological assemblies (HA trimer, NA tetramer, M2 tetramer) and the centroids of each type of lipid molecule.

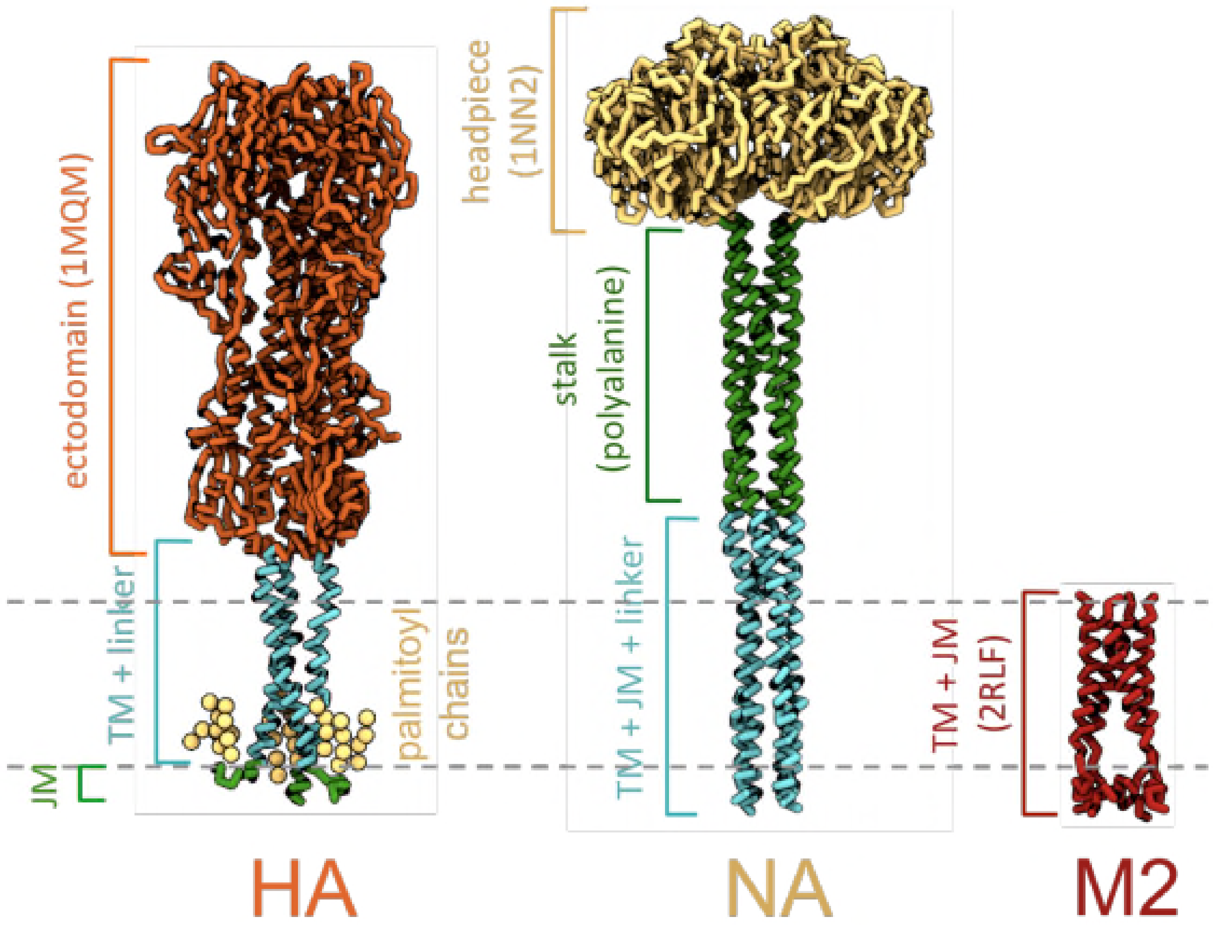

Figure S18: (Related to Figure 1) CG representations of the three species of influenza A envelope protein. The gray broken lines indicate the approximate location of the bilayer headgroups. **HA**: the ectodomain (orange) was derived from the X-ray structure (PDB code: 1MQM) (Ha et al., 2003); the linker and TM domain (cyan) were modeled as α-helix; the C-terminal tail (green) was modeled as an unstructured region with attached palmitoyl tails (yellow). **NA**: the headpiece (yellow) was derived from the X-ray structure (PDB code: 1NN2) (Varghese and Colman, 1991); the stalk (green) was modeled as a polyalanine coiled coil; the linker, TM domain, and N-terminal tail (cyan) were modeled as α-helix. **M2**: the model was derived from the NMR structure (PDB code: 2RLF) (Schnell and Chou, 2008).

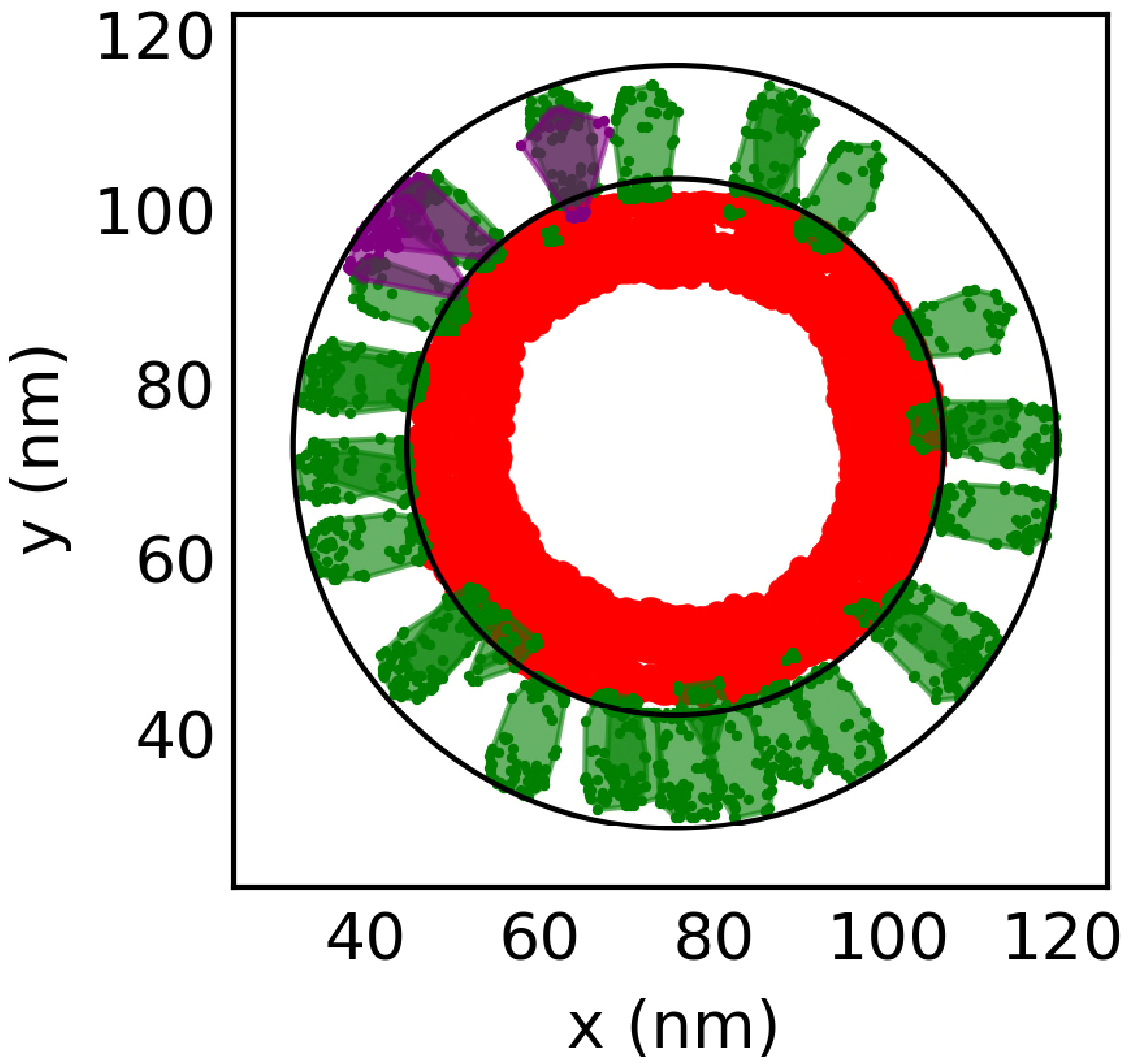

Figure S19: (Related to Figure 9) 20 nm cross-sectional slice through the Z-axis of an influenza A computational model demonstrating the three volumes required to calculate the fraction of the outer surface layer volume (V_f) occupied by the spike glycoproteins. $V_f = (V_{HA} + V_{NA})/V_{ring}$

Supplemental Computational Procedures

Simulation details. The refined procedure for virion model construction starts by alternating Packmol (Martinez et al., 2009) packing steps and GROMACS energy minimization steps to produce a lipid vesicle of the appropriate size and composition. The target starting diameter is actually much larger than the lower bound of the experimental estimate of the outer diameter, in order to avoid severe steric clashes. All vesicle / virion simulations were performed using GROMACS 4.5.5 (Hess et al., 2008) and the MARTINI 2.1 forcefield (Marrink et al., 2007; Monticelli et al., 2008). The forcefield was modified to produce the following custom particles / molecules: the restrained shell particle (RPO: attractive to water, super repulsive to other particles) (Parton, 2011), ether-linked dioleoyl phosphatidylethanolamine (DOPX: mimic ether-linkage at C1 position by changing particle 4 in DOPE from type Na to N0), hydroxylated sphingomyelin (PPCS) (PPCH: mimic hydroxyl group at PPCS particle 5 by adjusting from particle type C1 to Nda). RPO particles were always position-restrained in each dimension with a force constant of 10^3 kJ mol^{-1} nm^{-2}. The Forssman glycolipid was parametrized in two sections. First, the ceramide backbone was based on the matching particles provided for sphingomyelin in the MARTINI forcefield. Second, the headgroup of the Forssman glycolipid was comprised of the monosaccharides glucose, galactose, and N-acetylgalactosamine, with the glycosidic linkages as follows: GalNac-α (1-3) – GalNac-β (1-3) – Gal α (1-4) – Gal β (1-4) – Glc β (1-1) - Cer. The initial monosaccharide parameters were based on the sweet MARTINI forcefield (López et al., 2009) and previous in-house parametrization of the glycolipid GM3 (D.S. and H.K., personal communication). All sugars were initially represented as triangles, consisting of polar (P) particles. The side chains of N-acetylgalactosamine are more complex, and were represented as Nda particles given their similarity to an amino acid backbone. The five headgroup carbohydrates were connected using 'rotational nodes,' while the connection between the carbohydrates and ceramide backbone was parametrized by comparison to all-atom simulations using the GLYCAM forcefield (Kirschner et al., 2008). CG particles were mapped onto the atomistic results to compare bonds, angles,

and dihedral angles. Force matching between the two resolution scales was performed to tune the CG force constants for the Forssman glycolipid. Most properties of the atomistic system were successfully reproduced in the CG representation using an iterative refinement process. Similar parametrizations of PIP2 (Stansfeld et al., 2009) and cardiolipin (Dahlberg and Maliniak, 2010) have accurately reproduced lipid binding sites in crystal structures. The HA, NA and M2 proteins were modelled as described previously (Parton, 2011). Production simulations employed 5×10^8 steps with 10 fs time steps, and the composition of each system is summarized in Figure S3. Trajectory coordinates were written every 10^4 steps (every 0.1 ns). Coulomb and VDW interactions were respectively shifted off between 0.0 and 1.2 nm and 0.9 and 1.2 nm. Acylated proteins, lipids, RPO particles, and solvent were separately temperature coupled using the Berendsen algorithm (Berendsen et al., 1984) with a 1.0 ps time constant. Isotropic pressure coupling was performed using the Berendsen algorithm with a 1.1 ps time constant, 1×10^{-6} bar^{-1} compressibility, and a 1.0 bar reference pressure. In the case where all 107 viral membrane proteins were restrained, each of the proteins was subject to center of mass pulling (in all 3 dimensions) using an umbrella potential in GROMACS. The pull force (harmonic force constant, $k = 10^5$ kJ mol^{-1} nm^{-2}) was exerted in the direction of an absolute reference point at the origin, with initial pull vector set to (0,0,0) and pull rate set to 0 nm / ps (immobilized reference) for each protein. Forces and center of mass values from the pull code were written every 10^4 steps (every 0.1 ns).

Protein Models.

HA model.

The model of HA (Figure S18) was that described previously (Parton et al., 2013). Briefly, the model was based on the X-ray structure (PDB code: 1MQM) of the protein from the A/duck/Ukraine/1/63 (H3N8) influenza strain (Ha et al., 2003), which was converted to the CG representation. The X-ray structure does not include

the TM domain or the cytoplasmic domains, or a short linker between the ectodomain and TM domain. This missing region was modeled as α-helix. Palmitoyl chains were added to residues Cys555, Cys562 and Cys565 of the TM domain. The resultant model of the intact HA trimer was then simulated in a bilayer patch, allowing for relaxation of the structure. To maintain the protein tertiary structure, elastic network restraints were applied with the ElNeDyn tool (Periole et al., 2009), using a cutoff of 1.4 nm and force constants of 1000 kJ mol^{-1} nm^{-2}. The cytoplasmic domain was treated as unstructured and excluded from the restraint network.

NA model.

The NA stalk domain was modelled as a polyalanine coiled coil (based on a tetrameric coiled coil motif from the GCN4 leucine zipper protein (PDB code: 1GCL (Harbury et al., 1993))), with its length (approx. 10 nm) matched to cryo-EM images of the protein (Harris et al., 2006). In reality, the stalk domain of NA is thought to be mostly unstructured, with inter-subunit disulfide bonds stabilizing the tetrameric arrangement (Blok and Air, 1982). However, the exact sequence is largely unconserved and mutations of long stretches can be accepted without compromising the viability of the protein or virus (Luo et al., 1993). In contrast, the length of the stalk is thought to be an important factor; long deletions attenuate the growth rate and alter the host specificity of the virus (Castrucci and Kawaoka, 1993; Luo et al., 1993; Matsuoka et al., 2009).

M2 model.

The M2 proton channel is a 97-residue tetrameric TM protein. The CG model was derived directly from a NMR structure of the protein in a micellar environment (PDB code: 2RLF) (Schnell and Chou, 2008). This structure was solved for a construct comprising residues 18-60, which includes 15 residues of the C-terminal cytoplasmic

tail as well as the α-helical TM domain. The protein is normally palmitoylated at C50 (Sugrue et al., 1990), but this residue was mutated to a serine in the construct used to derive the NMR structure. Palmitoyl chains were thus not included in the CG model. Palmitoylation has been found to have no effect on viral replication in vitro, although it may provide some contribution to virulence in vivo (Grantham et al., 2009).

Analysis of trajectories. Particle coordinates in trajectory files were exposed using the open source Python MDAnalysis library (Michaud-Agrawal et al., 2011) and analyzed using in-house Python code. We also used IPython (Pérez and Granger, 2007), numpy (Oliphant, 2007), SciPy (Jones et al., 2001–), scikit-learn (Pedregosa et al., 2011) and matplotlib (Hunter, 2007) for scientific computing in Python. VMD (Humphrey et al., 1996) and PyMOL (Schrödinger, LLC, 2010) were used for visualization.

Incorporation of viral proteins into equilibrated vesicle. Models of the HA, NA and M2 proteins were obtained as previously described (Parton et al., 2013; Parton, 2011). Two coordinate files were used to start the procedure. The first contained the equilibrated vesicle and its RPO core with solvent removed from the system. The second contained only proteins: 80 HA trimers, 12 NA tetramers, and 15 M2 tetramers, with an approximately equidistant distribution about the surface of the equilibrated vesicle. The distribution and alignment of proteins was performed using a point-charge separation-based approach (Parton, 2011; Tatham, 2013). The two coordinate files were merged to produce a single set of coordinates with the appropriately-placed proteins *superposed* on the equilibrated vesicle. The merged coordinates were adjusted such that the principal axis of the first protein membrane-embedding candidate (1 of the 107 proteins) was aligned along the positive Z-axis along with the center of the RPO core placed at the origin. At this stage, all proteins except the embedding candidate are stripped from the coordinate file and their coordinates are stored. However, in subsequent embedding rounds, all proteins which are already embedded are permitted to remain in the sytem for the embedding process, otherwise the lipids may collpase back into the previously-occupied spaces. In

short, only 1 *superposed* protein is permitted in the system for embedding–the protein to be embedded. The g_membed program (Wolf et al., 2010) was then used to embed a given protein in the vesicle membrane using a fractional xy starting size of 0.1 and expanding to full horizontal size in 1000 steps, using a probe radius of 0.8 nm. NVT conditions (rather than NPT) were employed for embedding to preserve the box dimensions and simplify the reintegration of the remaining *superposed* coordinates back to the newly-embedded system, until all proteins were embedded.

Forssman glycolipid incorporation with Alchembed. An unpublished in-house procedure (named Alchembed) was used to exploit the free energy machinery (Beutler et al., 1994) of GROMACS for progressive activation of the non-bonded interactions of FORS glycolipid residues. 1000 molecular dynamics steps using a 1 fs integration timestep were performed with an increase in scaling factor ($\Delta\lambda$) of 10^{-3} per step. With λ starting at zero, the particles of the FORS glycolipid were therefore gradually incorporated into the sytem until both VDW and Coulomb intermolecular interactions were fully activated ($\lambda = 1$). Nonbonded interactions between and within FORS particles were also switched off initially and gradually activated during the simulation. Position restraints were applied only to the ceramide backbone of FORS with a force constant of 5 kJ mol^{-1} Å^{-2} and to RPO with a force constant of 10 kJ mol^{-1} Å^{-2}. Protein, lipid, RPO and FORS particles were separately temperature-coupled to an external bath using the Berendsen thermostat at 323 K with a time constant of 1.0 ps. The Berendsen barostat was used for isotropic pressure coupling at a reference pressure of 1.0 bar, a compressibility of 10^{-6} bar^{-1} and a coupling constant of 1.1 ps. The soft-core alpha parameter was 0.5 and the power for λ in the soft-core function was 1.0.

Sphericity tracking analysis. Sphericity (Ψ) was formally defined as a measure of the roundness of a particle in the context of geology (Wadell, 1935) (Equation (1)).

$$\Psi = \frac{\pi^{\frac{1}{3}}(6V_p)^{\frac{2}{3}}}{A_p} \tag{1}$$

Here, V_p is the volume of the particle and A_p its surface area. For every tenth frame (every 1 ns) of each vesicle or virion trajectory, the full set of lipid particle coordinates was selected. The convex hull of the lipid coordinates, conceptually similar to the outer surface coordinates, was calculated using an algorithm implemented in SciPy (Jones et al., 2001–), which exposes the vertices of the triangular facets of the convex polytope. The coordinates of these vertices were translated such that the Cartesian centroid of all facet vertices was at the origin. The surface area (A_p) of the convex hull was calculated by summing the areas from all triangular facets in a given frame. Each triangular facet was then treated as the base of a tetrahedron with an apex at the origin. As the system has been translated to place the origin within the convex hull, the sum of the absolute values of the volumes of each component tetrahedron can be used to calculate the volume (V_p) of the convex hull (*i.e.,* estimate volume bounded by lipids). Each individual tetrahedral volume is calculated based on a linear determinant of the triangular facet coordinates, which is simpler with a vertex at the origin (Equation (2)). The sphericity value in a given frame was then calculated using Equation (1).

$$V_p = \frac{1}{6}\begin{vmatrix} x_1 & y_1 & z_1 \\ x_2 & y_2 & z_2 \\ x_3 & y_3 & z_3 \end{vmatrix} \tag{2}$$

Outer diameter tracking. At 100 frame (10 ns) intervals, the full set of hydroxylated sphingomyelin (PPCH) molecule PO_4 particles was selected. Another selection made for each parsed frame was for all particles excluding the RPO core. The centroid of the latter set of particles was taken to represent the centroid of the vesicle (centroid of all lipids) or virion (centroid of all lipids + proteins) in a given frame, with RPO particles

having been excluded because the RPO core is often not centrally located due to drift of the virion relative to this restrained body of particles. A distance matrix was calculated between the full set of phosphate particles and the centroid. Double the average value of all phosphate radial distances was taken as an estimate of the outer diameter of the vesicle or virion.

Stratification tracking analysis. For every frame (every 0.1 ns) of each parsed replicate trajectory, the centroid of all lipid species in the system was determined. The PO_4 particles of a given lipid species or the ROH particles of CHOL were also selected. A distance matrix was calculated between the full set of PO_4 or ROH particles and the lipid centroid of the system. These radial distances were histogrammed in 0.5 nm bins between 0 and 80 nm, and converted to values as % of the given lipid population within a particular radial distance bin. The % values were countour plotted using logarithmic levels of base 2 varying between powers of -8.0 and 6.0.

Protein mobility tracking. The mobility of each of the 107 membrane proteins in flu virions at 295 K, and with the presence or absence of restraints on protein motion, was analysed in every 100 frames (every 10 ns) of the relevant trajectories. 107 protein centroids were determined in each parsed frame to simplify the tracking of any given protein, and there was no correction for any minor rotational or translational motion of the overall virion. The analysis was substantially more efficient as a result of manual indexing of the coordinate arrays rather than using built-in MDAnalysis selection and centroid functions.

Protein positional probability 3D mapping. For every frame (every 0.1 ns) in every parsed virion trajectory, the protein coordinates were obtained. The coordinate arrays for each protein type were individually histogrammed over 80 bins in each dimension of the full systems–100 nm side-length cubes (each bin is a cube with 12.5 Å side-length). After the first frame, coordinate histograms were still produced, but were combined with the histrogram from the previous frame. Compounding histograms on a per-frame basis vastly reduces the memory consumption of the code.

Diffusion analysis. Lipid and protein mean square displacement (MSD) values were calculated over a range of window sizes: 1, 3, 5, 10, 25, 50, 100, 200, 300, 400, and 500 ns. Diffusion constants were estimated from a least squares first degree (linear) polynomial fit of the MSD vs time data, either using the full complement of window sizes or excluding potentially ballistic motion at smaller window sizes ($\leq$ 50 ns). 50 ns is well outside the previously-described cutoff (30 ns) for anomalous diffusion in CG lipid simulations (Goose and Sansom, 2013). The uncertainty in the calculated diffusion constants was estimated as the difference of the linear slopes from the first and second halves of the data, which is the approach used by the GROMACS tools function g_msd. Lipid diffusion constants were calculated using both the centroid of all particles in each lipid or using only a single CG headgroup particle. For protein diffusion constant calculations, only the centroids of the biological assemblies were used. The scaling exponent (α) was estimated as the slope of the log MSD vs log time data. A second set of diffusion calculations were performed assuming anomalous diffusion ($\alpha \neq 1$) by non-linear least squares fitting to the two parameter equation (3) described previously (Kneller et al., 2011):

$$\text{MSD} = 4D_\alpha t^\alpha, 0 < \alpha < 2 \tag{3}$$

Where D_α is measured in units of length^2/time$^\alpha$. The standard deviations of both parameters were taken from the square root of the diagonal of the covariance matrix from the non-linear least squares fit.

Radial Distribution Functions. Radial distribution functions (RDFs) were calculated between lipid tail particles and bulk solvent molecules in each of the influenza CG-MD simulation replicates for between the final 2 ns and final 114 ns. The variety of time windows is the result of compromise for the extremely large system size and distance matrices which make the calculations extremely slow. The lipid residues and their assigned tail particles for the purposes of the RDFs: DUPC (C4B), DPPC (C4B), POPS (C5B), DOPX (C5B), DOPE (C5B), FORS (C4B), and PPCH (C4B). CHOL was not included in these analyses. Solvent selections were

limited to W, WF or ION particles within 100.0 Å of the tail particles in each frame (to manage the size of the distance matrix). Lipid-solvent distances were categorized into one of 200 bins between 0 and 110.0 Å, with calculations following multicore and normalization strategies described previously (Levine et al., 2011; Michaud-Agrawal et al., 2011), and with verification of results against GROMACS tools function g_rdf for a single frame. A similar algorithm was employed for calculation of RDFs between the TMD region centroids of the three types of influenza protein (HA, NA, M2) and the centroids of the lipid molecules of each type in each condition, for both the first and last 100 ns of a given simulation.

Sialic acid binding site surface distance and angle calculations. Three sialic acid (SA) binding sites per HA trimer were assigned based on the proximity of residues to (or their direct inclusion in) the canonical 220 loop (Q226, P227, G228), 130 loop (S136, S137, A138) or 190 helix (Y98, W153, H183) (Ha et al., 2003). The overall centroid of the pertinent CG residue centroids in a given monomer was used to define a single coordinate representing the SA binding site. Although SA surface distance and angle calculations have been described for filamentous virions (Wasilewski et al., 2012), our algorithm for spherical virions was slightly different. All coordinates were translated such that the centroid of all virion lipid particles was placed at the origin. Then an iterative procedure was applied to each of the 80 HA trimers on the surface of a given virion in a snapshot taken near the end of the simulation. First, the centroid of the three SA sites in the HA trimer is calculated. The angle and axis of rotation are calculated for alignment with the +Z axis for the vector passing through the origin and the reference SA site centroid. After applying the same coordinate transformation to the other SA site coordinates a planar surface is placed at the maximum of all SA site Z coordinates. For all 240 SA binding sites in the current reference frame, the restriction on rotation $\phi \leq 35^{\circ}$ relative to the reference HA axis was enforced to match the previous calculations on filamentous virions. The surface distance was calculated as the difference in Z coordinates between the planar surface and the SA site that falls within the ϕ restriction region. For each of the 80 attack angles of a given virion the six closest SA binding site distances and their

corresponding rotations (ϕ) were recorded and final average and standard deviation values are reported after splitting to the top three (closest HA) and next three (closest neighbour HA) SA binding site surface distances. Final reported distance and ϕ values represent an accumulation over all 80 HA reference frames (virion attack orientations). We also performed a similar calculation that included the NA active and secondary sites that can associate with SA (Colman et al., 1983; Sung et al., 2010).

Spike glycoprotein fractional surface volume calculation. We defined the outer surface of a given virion in the final frame of its trajectory as the space bounded between the outer leaflet and a perimeter 13 nm (length of 1 HA molecule) beyond, for consistency with previous experimental data (Wasilewski et al., 2012). The radius of the outer leaflet was defined as the average of the 20 largest CG PO_4 particle distances from the centroid of all lipids in the virion. The total volume of the outer surface layer was then calculated as the difference in the volumes of the two bounding spheres. We calculated the convex hulls of the CG particles representing the spike glycoproteins, excluding any CG particles that fall outside the region bounded by the two spheres. The volumes of the convex hulls of the spike glycoproteins (HA and NA) were individually calculated by summing the volumes of tetrahedra encompassing a facet of the convex hull with a vertex at the origin, similar to the process described for the sphericity calculations. The sum of all spike glycoprotein volumes was represented as a % of the total volume of the outer surface layer (Figure S19).

Protein separation distances on virion surfaces. Two separate virion surface interprotein distance calculations were performed–in the presence or absence of the M2 protein, primarily because the simulations allow for access to the positions of M2 proteins while the published experimental separation data does not (Wasilewski et al., 2012). In either case, the full distance matrix between all proteins, specifically the centroids of their constituent CG particles, was calculated in the first and final frames of a given simulation. The distances to the five closest neighbouring proteins were stored for each individual protein on the virion surface, and categorized

into one of 20 bins in the distance range bounded by 50 and 250 Å for the histograms reported in this work. The average, standard deviation, mode and total number of distances used for the full set of closest five neighbour calculations of a given virion are also reported.

Protein and lipid clustering analysis. The protein clustering analysis was performed on the full set of 107 influenza membrane protein centroids, and was not subdivided by protein type, for the first and final time points of each protein-inclusive simulation condition. The data was preprocessed by removing the mean and scaling to unit variance. The DBSCAN clustering algorithm implemented in scikit-learn (Pedregosa et al., 2011) was applied over the parameter value ranges of $0.2 \leq \epsilon < 1.2$ in increments of 0.1 and $3 \leq min\_samples < 12$ in increments of 1. The best result was selected on the basis of the maximal silhouette coefficient (s) calculated by the scikit-learn metrics library, where $-1 \leq s \leq 1$, and negative values indicate problematic cluster assignments, values near 0 indicate overlapping clusters, and values closer to 1 indicate a higher confidence in cluster assignments. The optimal cluster assignments were only analyzed and plotted if more than one cluster could be assigned for a given condition. A similar algorithm was employed for the clustering analysis of lipids, except that lipids were categorized based on both their species and their leaflet in the viral envelope. The leaflet assignments of lipid species were performed based on distance of headgroup particles from virion centroid, with inner leaflet within 27 nm and outer leaflet beyond that distance.

Supplemental References

Berendsen, H., Postma, J., Vangunsteren, W., Dinola, A., and Haak, J. (1984). Molecular-dynamics with coupling to an external bath. J. Chem. Phys. *81*, 3684–3690.

Beutler, T.C., Mark, A.E., van Schaik, R.C., Gerber, P.R., and van Gunsteren, W.F. (1994). Avoiding singularities and numerical instabilities in free energy calculations based on molecular simulations. Chemical Physics Letters *222*, 529 – 539.

Blok, J., and Air, G.M. (1982). Variation in the membrane-insertion and stalk sequences in eight subtypes of influenza type A virus neuraminidase. Biochemistry *21*, 4001–4007.

Castrucci, M.R., and Kawaoka, Y. (1993). Biologic importance of neuraminidase stalk length in influenza A virus. J. Virol. *67*, 759–764.

Colman, P.M., Varghese, J.N., and Laver, W.G. (1983). Structure of the catalytic and antigenic sites in influenza virus neuraminidase. Nature *303*, 41–44.

Dahlberg, M., and Maliniak, A. (2010). Mechanical properties of coarse-grained bilayers formed by cardiolipin and zwitterionic lipids. J. Chem. Theory Comput. *6*, 1638–1649.

Gerl, M.J., Sampaio, J.L., Urban, S., Kalvodova, L., Verbavatz, J.M., Binnington, B., Lindemann, D., Lingwood, C.A., Shevchenko, A., Schroeder, C., *et al.* (2012). Quantitative analysis of the lipidomes of the influenza virus envelope and MDCK cell apical membrane. J. Cell Biol. *196*, 213–221.

Goose, J.E., and Sansom, M.S.P. (2013). Reduced lateral mobility of lipids and proteins in crowded membranes. PLoS Comput. Biol. *9*, e1003033.

Grantham, M.L., Wu, W.H., Lalime, E.N., Lorenzo, M.E., Klein, S.L., and Pekosz, A. (2009). Palmitoylation of

the influenza A virus M2 protein is not required for virus replication in vitro but contributes to virus virulence. J. Virol. *83*, 8655–8661.

Ha, Y., Stevens, D.J., Skehel, J.J., and Wiley, D.C. (2003). X-ray structure of the hemagglutinin of a potential H3 avian progenitor of the 1968 hong kong pandemic influenza virus. Virology *309*, 209 – 218.

Harbury, P., Zhang, T., Kim, P., and Alber, T. (1993). A switch between two-, three-, and four-stranded coiled coils in gcn4 leucine zipper mutants. Science *262*, 1401–1407.

Harris, A., Cardone, G., Winkler, D.C., Heymann, J.B., Brecher, M., White, J.M., and Steven, A.C. (2006). Influenza virus pleiomorphy characterized by cryoelectron tomography. Proc. Natl. Acad. Sci. U. S. A. *103*, 19123–19127.

Hess, B., Kutzner, C., van der Spoel, D., and Lindahl, E. (2008). Gromacs 4: Algorithms for highly efficient, load-balanced, and scalable molecular simulation. J. Chem. Theory Comput. *4*, 435–447.

Humphrey, W., Dalke, A., and Schulten, K. (1996). VMD – Visual Molecular Dynamics. J. Mol. Graphics *14*, 33–38.

Hunter, J.D. (2007). Matplotlib: A 2d graphics environment. Comput. Sci. Eng. *9*.

Jones, E., Oliphant, T., Peterson, P., *et al.* SciPy: Open source scientific tools for Python (2001–). URL `http://www.scipy.org/`.

Kirschner, K.N., Yongye, A.B., Tschampel, S.M., González-Outeirio, J., Daniels, C.R., Foley, B.L., and Woods, R.J. (2008). GLYCAM06: A generalizable biomolecular force field. Carbohydrates. J. Comput. Chem. *29*, 622–655.

Kneller, G.R., Baczynski, K., and Pasenkiewicz-Gierula, M. (2011). Communication: Consistent picture of

lateral subdiffusion in lipid bilayers: Molecular dynamics simulation and exact results. J. Chem. Phys. *135*, 141105.

Levine, B.G., Stone, J.E., and Kohlmeyer, A. (2011). Fast analysis of molecular dynamics trajectories with graphics processing units-radial distribution function histogramming. J. Comput. Phys. *230*, 3556 – 3569.

López, C.A., Rzepiela, A.J., de Vries, A.H., Dijkhuizen, L., Hnenberger, P.H., and Marrink, S.J. (2009). Martini coarse-grained force field: Extension to carbohydrates. J. Chem. Theory Comput. *5*, 3195–3210.

Luo, G., Chung, J., and Palese, P. (1993). Alterations of the stalk of the influenza virus neuraminidase: deletions and insertions. Virus Res. *29*, 141–153.

Marrink, S.J., Risselada, H.J., Yefimov, S., Tieleman, D.P., and de Vries, A.H. (2007). The MARTINI force field: coarse grained model for biomolecular simulations. J. Phys. Chem. B *111*, 7812–7824.

Martinez, L., Andrade, R., Birgin, E.G., and Martinez, J.M. (2009). PACKMOL: a package for building initial configurations for molecular dynamics simulations. J Comput Chem *30*, 2157–2164.

Matsuoka, Y., Swayne, D.E., Thomas, C., Rameix-Welti, M.A., Naffakh, N., Warnes, C., Altholtz, M., Donis, R., and Subbarao, K. (2009). Neuraminidase stalk length and additional glycosylation of the hemagglutinin influence the virulence of influenza H5N1 viruses for mice. J. Virol. *83*, 4704–4708.

Michaud-Agrawal, N., Denning, E.J., Woolf, T.B., and Beckstein, O. (2011). MDAnalysis: A toolkit for the analysis of molecular dynamics simulations. J. Comput. Chem. *32*, 2319–2327.

Monticelli, L., Kandasamy, S.K., Periole, X., Larson, R.G., Tieleman, D.P., and Marrink, S.J. (2008). The MARTINI Coarse-Grained Force Field: Extension to Proteins. J. Chem. Theory Comput. *4*, 819–834.

Oliphant, T.E. (2007). Python for scientific computing. Comput. Sci. Eng. *9*.

Parton, D.L., Tek, A., Baaden, M., and Sansom, M.S.P. (2013). Formation of raft-like assemblies within clusters of influenza hemagglutinin observed by MD simulations. PLoS Comput. Biol. *9*, e1003034.

Parton, D. (2011). Pushing the boundaries: molecular dynamics simulations of complex biological membranes. PhD thesis (Oxford, Oxon : University of Oxford).

Pedregosa, F., Varoquaux, G., Gramfort, A., Michel, V., Thirion, B., Grisel, O., Blondel, M., Prettenhofer, P., Weiss, R., Dubourg, V., *et al.* (2011). Scikit-learn: Machine learning in Python. Journal of Machine Learning Research *12*, 2825–2830.

Pérez, F., and Granger, B.E. (2007). IPython: a system for interactive scientific computing. Comput. Sci. Eng. *9*, 21–29.

Periole, X., Cavalli, M., Marrink, S.J., and Ceruso, M.A. (2009). Combining an elastic network with a coarse-grained molecular force field: Structure, dynamics, and intermolecular recognition. J. Chem. Theory Comput. *5*, 2531–2543.

Schnell, J.R., and Chou, J.J. (2008). Structure and mechanism of the M2 proton channel of influenza A virus. Nature *451*, 591–595.

Schrödinger, LLC. The PyMOL molecular graphics system, version 1.3r1 (2010.).

Stansfeld, P.J., Hopkinson, R., Ashcroft, F.M., and Sansom, M.S.P. (2009). PIP2-Binding Site in Kir Channels: Definition by Multiscale Biomolecular Simulations. Biochemistry *48*, 10926–10933.

Sugrue, R.J., Belshe, R.B., and Hay, A.J. (1990). Palmitoylation of the influenza A virus M2 protein. Virology *179*, 51–56.

Sung, J.C., Wynsberghe, A.W.V., Amaro, R.E., Li, W.W., and McCammon, J.A. (2010). Role of secondary sialic acid binding sites in influenza N1 neuraminidase. J. Am. Chem. Soc. *132*, 2883–2885.

Tatham, S. Simon Tatham's Home Page (2013). URL `http://www.chiark.greenend.org.uk/~sgtatham/`. [Online; accessed 24-June-2013].

Varghese, J., and Colman, P. (1991). Three-dimensional structure of the neuraminidase of influenza virus A/Tokyo/3/67 at 2.2 Å resolution. Journal of Molecular Biology *221*, 473–486.

Wadell, H. (1935). Volume, shape, and roundness of quartz particles. J. Geol. (Chicago, IL, U. S.) *43*, 250–280.

Wasilewski, S., Calder, L.J., Grant, T., and Rosenthal, P.B. (2012). Distribution of surface glycoproteins on influenza A virus determined by electron cryotomography. Vaccine *30*, 7368–7373.

Wolf, M.G., Hoefling, M., Aponte-Santamaría, C., Grubmüller, H., and Groenhof, G. (2010). g_membed: Efficient insertion of a membrane protein into an equilibrated lipid bilayer with minimal perturbation. J. Comput. Chem. *31*, 2169–2174.
