# Supplementary material for: Nothing to Sneeze At: A Dynamic and Integrative Computational Model of an Influenza A Virion
Source: Structure. 2015 Mar 3;23(3):584–97. doi: 10.1016/j.str.2014.12.019 (PMC4353694; doi:10.1016/j.str.2014.12.019)
Supplement: Document S7. Article plus Supplemental Information [file mmc7.pdf]

Theory

Structure

Nothing to Sneeze At: A Dynamic and Integrative Computational Model of an Influenza A Virion

Graphical Abstract

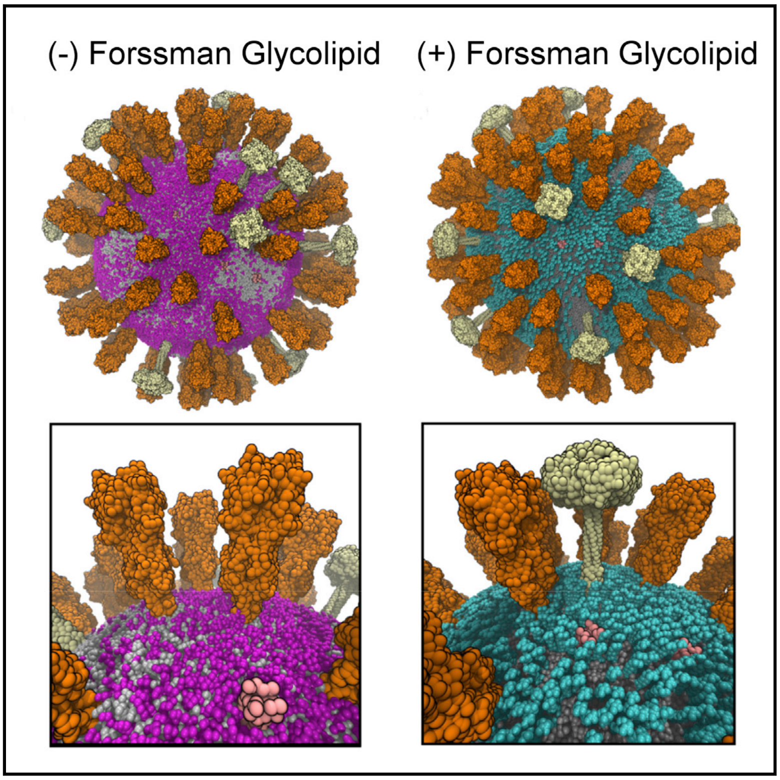

Highlights

- Microsecond timescale molecular dynamics simulations of enveloped influenza virion
- Forssman glycolipid alters the biophysical properties of the influenza A virion
- Spacing of the spike glycoproteins is compatible with bivalent antibody association
- Mobility of viral proteins influences distribution of lipids in the viral envelope

Authors

Tyler Reddy, David Shorthouse, ..., Marc Baaden, Mark S.P. Sansom

Correspondence

mark.sansom@bioch.ox.ac.uk

In Brief

Reddy et al. performed the first microsecond timescale coarse-grained molecular dynamics simulations of enveloped virions in explicit solvent. The simulated properties of the influenza A virion were consistent with experimental measurements, and revealed that the Forssman glycolipid affects several biophysical characteristics of the virion.

Reddy et al., 2015, Structure *23*, 584–597
March 3, 2015 ©2015 The Authors
http://dx.doi.org/10.1016/j.str.2014.12.019

Structure
Theory

Nothing to Sneeze At: A Dynamic and Integrative Computational Model of an Influenza A Virion

Tyler Reddy,[1] David Shorthouse,[1,3] Daniel L. Parton,[1,4] Elizabeth Jefferys,[1] Philip W. Fowler,[1] Matthieu Chavent,[1] Marc Baaden,[2] and Mark S.P. Sansom[1,*]
[1]Department of Biochemistry, University of Oxford, South Parks Road, Oxford OX1 3QU, UK
[2]Institut de Biologie Physico-Chimique, Centre National de la Recherche Scientifique, UPR9080, Université Paris Diderot, Sorbonne Paris Cité, Paris, France
[3]Present address: MRC Cancer Unit, University of Cambridge, Cambridge CB2 0XZ, UK
[4]Present address: Memorial Sloan Kettering Cancer Center, New York, NY, USA
*Correspondence: mark.sansom@bioch.ox.ac.uk
http://dx.doi.org/10.1016/j.str.2014.12.019
This is an open access article under the CC BY license (http://creativecommons.org/licenses/by/4.0/).

SUMMARY

The influenza virus is surrounded by an envelope composed of a lipid bilayer and integral membrane proteins. Understanding the structural dynamics of the membrane envelope provides biophysical insights into aspects of viral function, such as the wide-ranging survival times of the virion in different environments. We have combined experimental data from X-ray crystallography, nuclear magnetic resonance spectroscopy, cryo-electron microscopy, and lipidomics to build a model of the intact influenza A virion. This is the basis of microsecond-scale coarse-grained molecular dynamics simulations of the virion, providing simulations at different temperatures and with varying lipid compositions. The presence of the Forssman glycolipid alters a number of biophysical properties of the virion, resulting in reduced mobility of bilayer lipid and protein species. Reduced mobility in the virion membrane may confer physical robustness to changes in environmental conditions. Our simulations indicate that viral spike proteins do not aggregate and thus are competent for multivalent immunoglobulin G interactions.

INTRODUCTION

There have been a number of structural studies on the influenza A virus (e.g. Calder et al., 2010; Harris et al., 2006; Wasilewski et al., 2012), which is surrounded by a pleomorphic lipid bilayer envelope that imposes challenges for high-resolution structural characterization. These have provided important details about the morphology of the virions and the distribution of their surface glycoproteins, but structural studies that include detailed analysis of the lipids are lacking. Indeed, the lipid composition of the influenza A envelope has only recently been established (Gerl et al., 2012). The importance of lipids in the stability of the influenza A virion is clear from a number of studies. Both H5N1 and H1N1 viruses were more stable in water when grown in mammalian cells versus counterparts propagated in avian cells, even for viruses with the same genetic background (Shigematsu et al., 2014). Only the lipid composition and the glycosylation states of the viruses differed. A progressive ordering with decreasing temperature for influenza A lipids studied by nuclear magnetic resonance (NMR) spectroscopy implicated the lipids in seasonal behavior (Polozov et al., 2008). Lipids form much of the outer protective shell of the influenza A virion, and they are a logical target for additional biophysical analysis.

Molecular dynamics simulations provide an opportunity to integrate structural data from a variety of experimental sources. For example, an impressive set of 0.1 μs, 64 million atom, molecular dynamics simulations were used to model the HIV-1 capsid (Zhao et al., 2013). However, these simulations omitted the lipid envelope of the virus, enabling the method for model construction to be strongly guided by the experimental electron densities from cryo-electron microscopy (cryo-EM). A multiscale approach was used for examining the full-scale immature HIV-1 virion (Ayton and Voth, 2010). The system was highly coarse-grained (CG) with a protein model corresponding to approximately 7–9 amino acid residues per particle, and used a relatively simple (DOPS/DOPC) and symmetric lipid bilayer membrane. An all-atom simulation of a complete virus, including its RNA core, has also been performed (Freddolino et al., 2006), based on the crystal structure of satellite tobacco mosaic virus. This virus contains no lipid, and the viral envelope consists of 60 copies of a single protein arranged in an icosahedron. Recent modeling of nonenveloped icosahedral virions revealed their mechanical properties and possible mechanisms for capsid dissolution via calcium ion depletion (Larsson et al., 2012; Zink and Grubmüller, 2009, 2010). Likewise, recent modeling of the rabbit hemorrhagic disease virus (Wang et al., 2013), which is also icosahedral and contains no lipids, was based on fitting the model to available X-ray diffraction and cryo-EM data. Previous influenza virus membrane protein simulations have largely been focused on isolated components of the virion, e.g. modeling of fusion peptide activity (Risselada et al., 2012) or of hemagglutinin (HA) clustering in model membranes (Parton et al., 2013).

In this study, we use CG molecular dynamics simulations (Stansfeld and Sansom, 2011) building on structural information

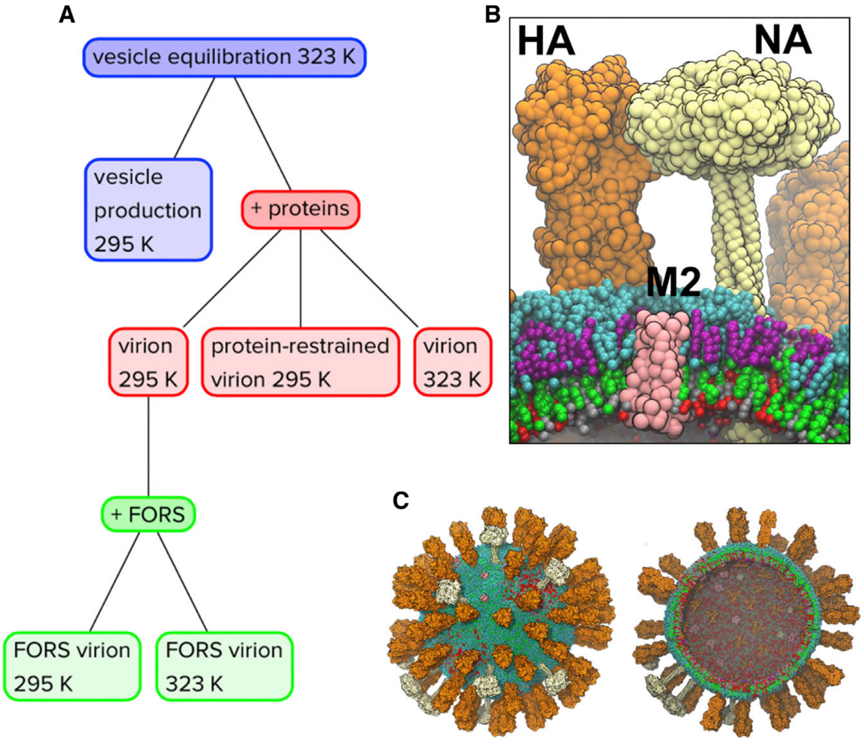

Figure 1. Influenza Virion Models and Simulations

(A) Flowchart summary of the influenza A virion simulations. The initial vesicle equilibration simulation was for 0.3 μs; all other simulations were for ~5 μs. The lipid species used include palmitoyl-oleoyl-phosphatidylserine (POPS), dioleoylphosphatidylethanolamine (DOPE), ether-linked DOPE (DOPX), cholesterol (CHOL), hydroxylated sphingomyelin (PPCH), and the Forssman glycolipid (FORS). All virion simulations including proteins contained 80 HA trimers, 12 NA tetramers, and 15 M2 tetramers. A restrained inner core of 31,767 particles was assembled into a hollow sphere to represent the approximate volume occupied by the nucleoprotein. The systems also contained ~5% antifreeze water.

(B) Zoom-in view of a representative region from the FORS-inclusive virion simulation at 323 K showing the HA (orange), NA (white), and M2 (pink) viral proteins in the context of the multicomponent asymmetric lipid envelope (CHOL: green; DOPE/X: red; FORS: cyan; POPS: silver; PPCH: purple).

(C) The starting configuration of the 295 K virion simulation. An outside view (left) and a cross-sectional view (right) are shown, the central core having been omitted from the latter diagram for clarity. See also Figures S1–S3 and S18. See also Supplemental Information Files S20–S24 for coordinate files corresponding to the virus models at the end of each of the five simulations.

from X-ray crystallography (Ha et al., 2003; Varghese and Colman, 1991), NMR spectroscopy (Schnell and Chou, 2008), cryo-EM (Harris et al., 2006), and lipidomics data (Gerl et al., 2012) to produce a detailed (near atomic resolution) computational model of the influenza A virion. This integration of structural information from a number of sources has allowed us to perform microsecond-scale CG molecular dynamics simulations of the outer envelope of an enveloped virion in explicit solvent. These simulations reveal the structural and dynamic properties of the viral envelope which contribute to its stability, and will allow us to initiate models of virion/target cell recognition. The complex lipid dynamics revealed in our simulations extend and complement static structural data from cryo-EM and related experimental approaches. We provide the virion coordinates and simulation parameters openly to the community.

RESULTS AND DISCUSSION

Influenza Virion Models and Progress of the Simulations

The construction of a computational model of the membrane envelope of a virion is in itself challenging and warrants some discussion. More generally, generating computational models of pleomorphic enveloped viruses, for which high-resolution structural detail of the entire virion is more difficult to obtain, has not been described in detail. The essence of the problem is to model a "moving target" at the same time as retaining available structural data, rather than fitting to reasonably well defined experimental density.

We started by generating an initial lipid bilayer vesicle model (Figure 1A), with a diameter of ~74 nm and with a lipid bilayer composition approximating the known viral lipidome (Gerl et al., 2012). This model includes the following lipid species: 15% palmitoyl-oleoyl-phosphatidylserine (POPS), 5% dioleoylphosphatidylethanolamine (DOPE), 9% ether-linked DOPE (DOPX), 53% cholesterol, and 18% hydroxylated sphingomyelin (PPCH), with a total of ~43,000 lipid molecules present. Using the MARTINI CG model (which has been widely used to model and simulate a wide range of membrane proteins and systems [Marrink and Tieleman, 2013]) this yields a simulation system of >5M particles (full coordinates available in Supplemental Information). We used a spherical hollow core of positionally restrained "RPO" particles to substitute for the ribonucleoprotein (RNP) core of the virion in all simulations. The shape of the virion would be anticipated to remain spherical in the absence of the RPO core. Previous CG simulations of vesicles of up to 20 nm outer diameter demonstrated near perfect sphericity on a microsecond timescale (Marrink and Mark, 2003), and short CG simulations of 190 nm outer diameter liposomes were stable in the absence of an internal core (Ayton and Voth, 2009a).

The initial vesicle model was simulated for 0.3 μs to equilibrate the packing of the lipids. There was a rapid (~50 ns) relaxation that resulted in shrinking of the vesicle (the lipids were initially loosely packed) and "repairing" of holes in the lipid bilayer. Thus, the outer diameter (Figure 2A) dropped from ~74 nm to ~59 nm, attaining a final size consistent with the lower bounds estimated from experiments discussed below. Monitoring the sphericity (Ψ) (Wadell, 1935) of the equilibrating vesicle revealed that there was a degree of deformation during the initial shrinking followed by a return to a more spherical shape (Ψ = 1) by the end of the equilibration period. As the shrinking of the vesicle is a nonequilibrium process, we do anticipate some pressure difference between the inside and

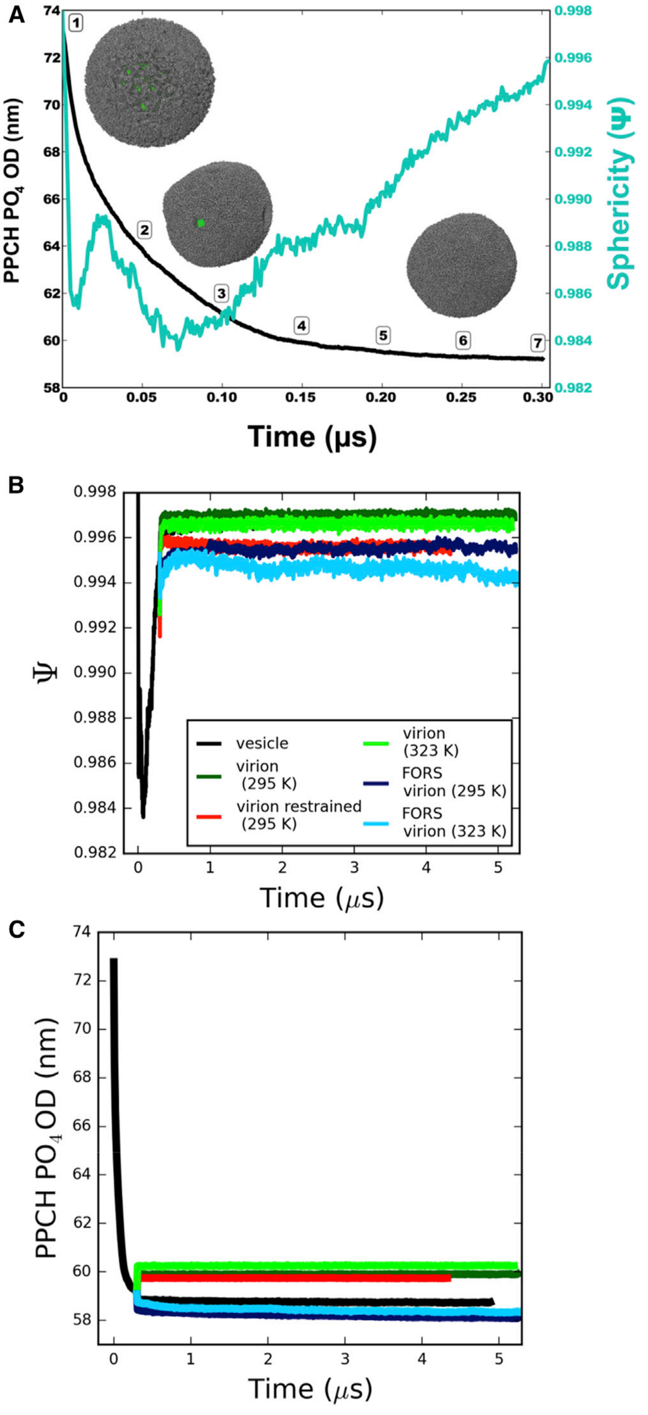

Figure 2. Progress of the Simulations

(A) Progress of the 0.3 µs vesicle equilibration simulation, tracking the outer diameter (black line, left-hand axis) and sphericity (cyan, right-hand axis) as functions of time. The outer diameter is calculated as twice the average radius of all hydroxylated sphingomyelin headgroup phosphate (PPCH PO_4) particles from the lipid centroid of the system. Seven time points are marked in 50 ns intervals. The inset images of the equilibrating vesicle (at time points 1, 3, and 6) have lipids shown in gray and the central core in green.

(B and C) Progress of the virion model simulations was also monitored by tracking sphericity (B) and outer diameter (C). The vesicle (black, temperature adjusted from 323 to 295 K at 0.3 µs), the virion (295 K, dark green; 323 K, light green), the protein-restrained virion (red), and the Forssman glycolipid inclusive virion (295 K, dark blue; 323 K, light blue) are shown. See also Figures S4 and S5.

outside of the structure, and the resulting membrane tensions may influence lipid dynamics.

The three species of influenza A integral membrane proteins (Figure 1B) were then inserted into the vesicle (Figure S1) to yield an initial virion model containing 80 hemagglutinin (HA) trimers, 12 neuraminidase (NA) tetramers, and 15 M2 tetramers. The copy numbers of HA and NA proteins were derived from their surface density in 3D tomograms of the virion (Harris et al., 2006). The number of M2 proteins derived from the lower bound of an experimental estimate of 15–25 channels per virion (Takeda et al., 2002). The HA models were palmitoylated at their C termini (Parton, 2011) (see also Supplemental Information). In two of the virion simulations, Forssman glycolipid (globopentosylceramide) was included in the outer leaflet of the lipid bilayer (Figures 1A and 3; Figure S2), resulting in an overall composition of 6% hydroxylated sphingomyelin (PPCH) and 12% Forssman glycolipid. The Forssman glycolipid is the most abundant sphingolipid in the influenza A virion (Gerl et al., 2012), with a ~2-fold greater abundance than sphingomyelin. The Forssman glycolipid has a terminal *N*-acetylgalactosamine in α1-3 linkage to the terminal *N*-acetylglucosamine of globoside (Stanley and Cummings, 2009).

The resultant membrane is relatively crowded, although a little sparser than for some cell membranes, e.g. ca. 25% of the area of red blood cell membranes is estimated to be protein (Dupuy and Engelman, 2008). Calculating the "fractional volume" of a spherical shell corresponding to the ectodomains of the spike proteins (see below for details) indicates they occupy ca. 15% of the viral membrane area (Wasilewski et al., 2012); thus the proteins do not completely cover the surface of the virion. By visual inspection, the Forssman glycolipid largely "covers" the bilayer surface (Figure 4B). In particular, the glycolipid largely masks the M2 channel proteins from the external surface of the virion. This may have implications in terms of the access of drug molecules (adamantanes), which act by blocking the M2 channel protein (Gu et al., 2013).

Based on the vesicle and virion models, a number of simulations were performed (Figure 1A; Figure S3). These explored both room (295 K) and elevated (323 K) temperatures, and also the presence or absence of the Forssman glycolipid (Figure 3), which is found in the influenza lipidome instead of 68% of the outer leaflet hydroxylated sphingomyelin molecules. We also performed a simulation in which the centroids of the membrane proteins were restrained, to mimic possible interactions with the inner matrix formed by the M1 matrix proteins of the virus (Veit and Thaa, 2011). However, it is useful to note that successful budding of approximately 10% of influenza A virions occurs with an apparent lack of the M1 layer, indicating that the simulations without protein restraints are of biological relevance, especially to the low pH, M1 detached, fusion-compatible state of the virus (Fontana and Steven, 2013). Thus we have what is perhaps the most realistic current computational model of an influenza A virion tractable for microsecond timescale simulation (Figure 4).

The progress of the simulations was monitored, including the sphericity and diameters of the lipid bilayer components of the

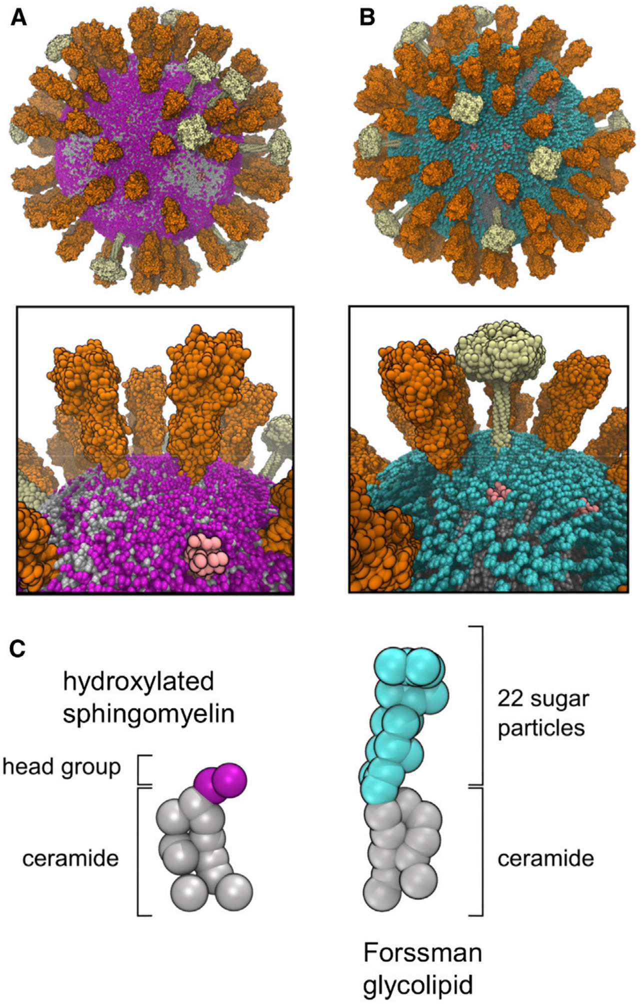

Figure 3. Virion Models and Glycolipids

(A and B) Visual comparison of virion starting models without (A) and containing (B) the Forssman glycolipid (*cyan*). Overall and zoom-in views of the respective virions are shown.

(C) Comparison of the CG representations of a hydroxylated sphingomyelin and a Forssman glycolipid. See also Figure S3.

models. In all cases, these values were consistent with an overall spherical geometry being retained, with some degree of influence on the shape of the virion caused by the presence or absence of the Forssman glycolipid (Figure 2B). Restriction of protein mobility via restraints caused a similar reduction in sphericity to the presence of glycolipid. This suggests a possible role for protein-lipid interactions in the shaping of the virion.

The diameters of the virion models were also all stable on a microsecond timescale (Figure 2C). Interestingly, virions were substantially smaller when glycolipid was present in place of sphingomyelin. This suggests that it is important to include glycolipids when constructing computational models of enveloped virions. We measured virion outer diameters using lipids, as they have a higher sampling density than the proteins. The range of simulation diameters (58–61 nm) for the lipid components of all the virion simulations was consistent with the experimentally determined minimum virion diameter of 84 nm (Harris et al.,

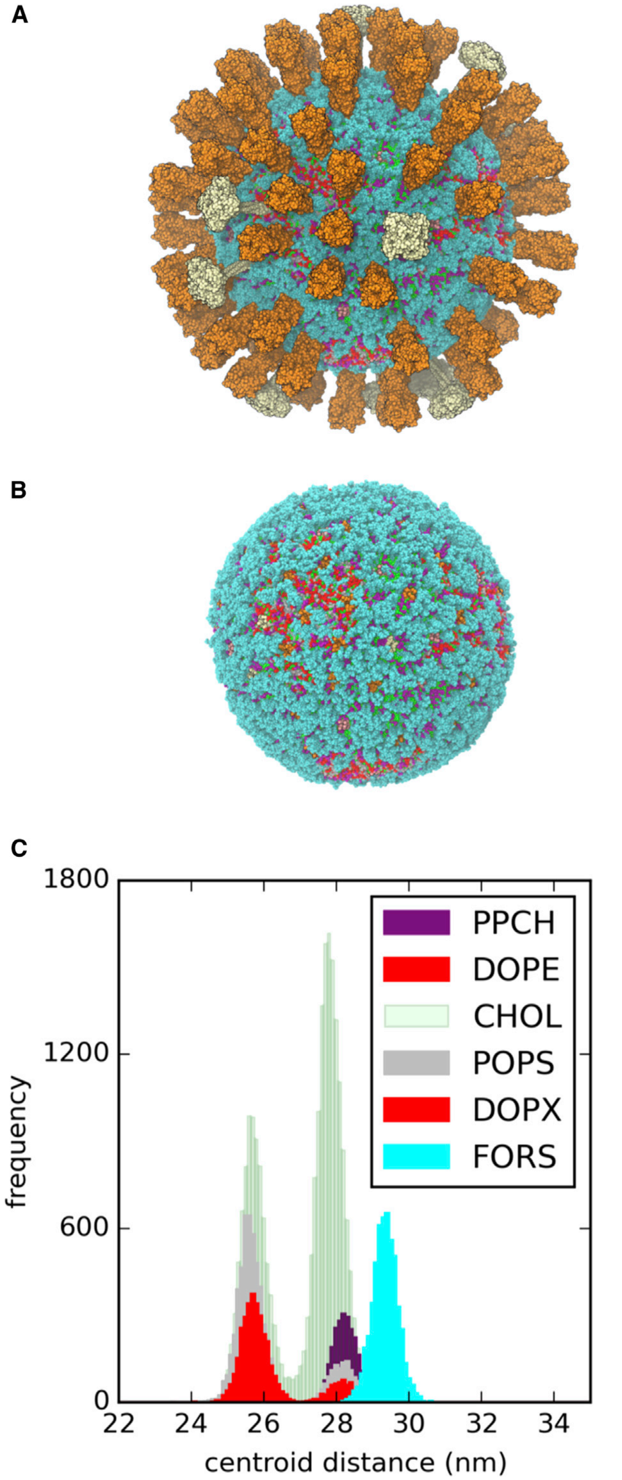

Figure 4. Snapshot at the End of the 295 K + Forssman Glycolipid Virion Simulation

(A and B) Protein ectodomains are shown in (A) and omitted for clarity in (B) (HA orange, NA white, M2 pink). The glycolipid is shown in cyan.

(C) The composition of lipid species and their distributions between the inner and outer leaflets of the bilayer are shown as a histogram of centroid distances from the overall virion centroid, with the colors approximately matched to those used for lipids in the structural snapshots.

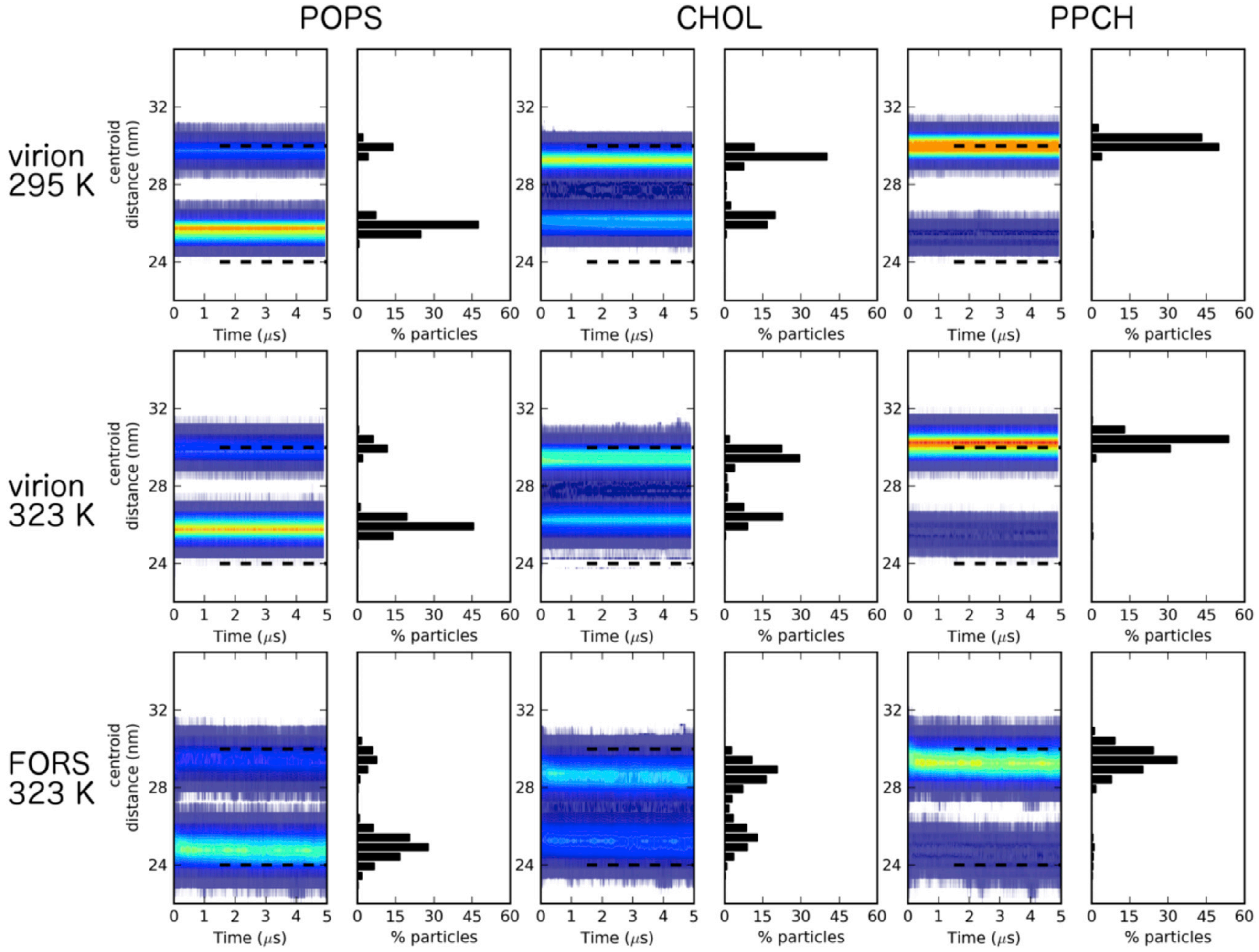

Figure 5. Distribution of Selected Lipids between the Outer and Inner Leaflets of the Bilayer

Heatmaps are shown for the time-dependent distributions of the distances between lipid species and the virion centroid. These are shown for representative inner leaflet (POPS, left), central (CHOL, middle), and outer leaflet species (PPCH, right). Phosphate particles were used for POPS and PPCH, while the ROH group was used for calculations with CHOL. The histogram data from the last analyzed frame of each replicate is shown to the right of each contour plot. See also Figures S6 and S17.

2006), after taking into account the 10 nm to 14 nm lengths of the two spike proteins (Schmitt and Lamb, 2005).

We also assessed the models on the basis of the extent of solvent penetration into the lipid envelope (Figure S4). In all cases, the degree of solvent particle penetration into the hydrophobic core of the lipid bilayer is low. This is clearest for the lipid (no protein) vesicle, while slightly more water penetrates in the case of the virion models. Interestingly it has been shown that enveloped vesicular stomatitis virions are more sensitive to osmotic stress before protease treatment (Bittman et al., 1976), suggesting that viral membrane proteins may play a role in maintaining envelope permeability.

We measured the distribution of the lipids between the two bilayer leaflets in each simulation, and observed stable leaflet populations for, e.g., PS as a representative inner leaflet lipid species, hydroxylated sphingomyelin as a representative outer leaflet lipid species, and cholesterol (Figure 5; Figures S5 and S6). In the presence of glycolipid or positional restraints on the membrane proteins a noticeably broader distribution of lipids was seen, indicating possible lipid mixing between leaflets. It is interesting that whether the membrane protein dynamics are reduced by restraining their motion directly or by inclusion of glycolipids (see discussion of diffusion coefficients below) the same effect is observed, suggesting that relatively immobile proteins may allow some degree of local perturbation of the structure of the lipid bilayer.

Diffusion of Lipids and Proteins

The influenza virion membrane provides an example of a complex biological membrane, differing from simpler model vesicle systems (Gambin et al., 2006) in that it contains multiple protein molecules and a complex mixture of lipid species, including a high (>50%) fraction of cholesterol. It is therefore of particular interest to use simulations to probe the diffusion dynamics of the proteins and lipids. Protein and lipid translational diffusion parameters were calculated from mean square displacement

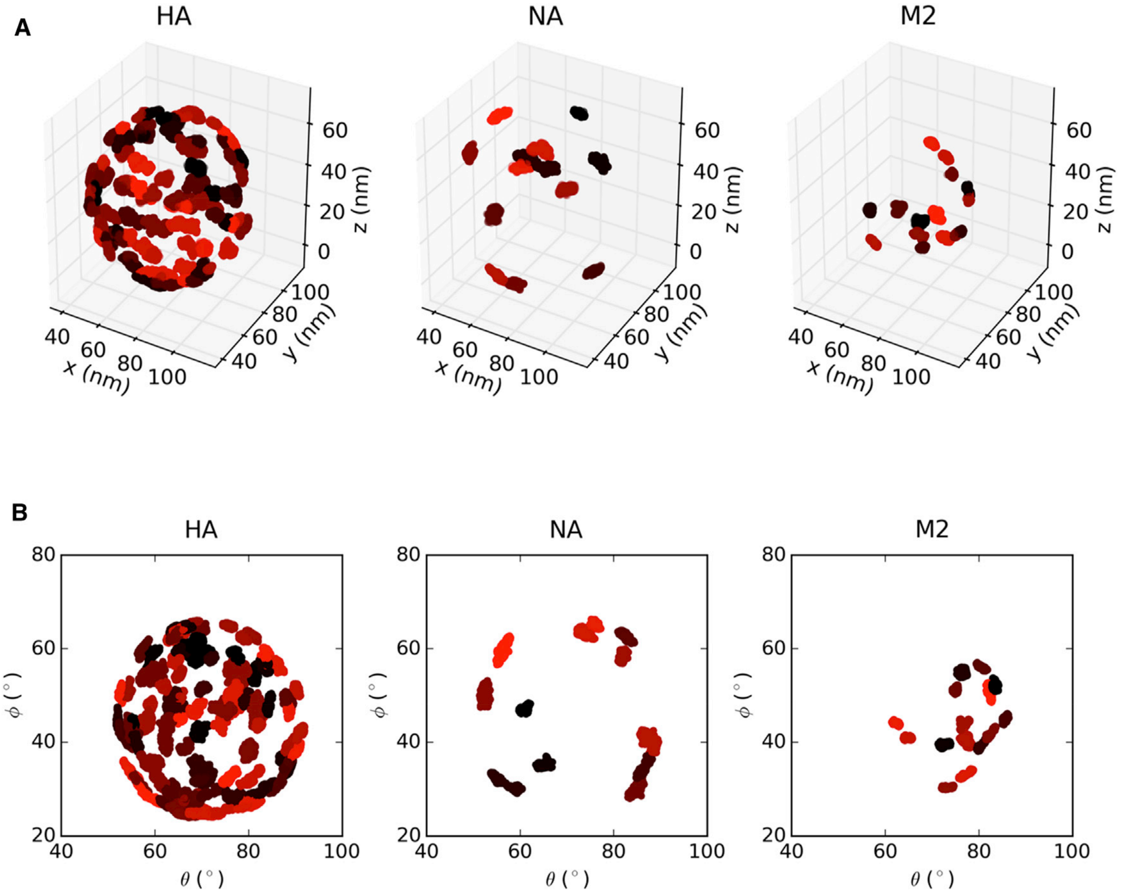

Figure 6. Protein Mobility during the Simulation

(A and B) The centroids of all 107 influenza membrane proteins were tracked over the duration of the virion simulation at 295 K excluding the Forssman glycolipid. The results are shown in Cartesian coordinate space (A) or in 2D spherical polar projection space (B). Each individual protein centroid is colored in a different shade for each of the three protein types. See also Figure S8.

(MSD) versus time data, interpreted either with a "normal" diffusion model (MSD = $4Dt$) or via an anomalous diffusion model in which the fractional diffusion constant (D_α) and scaling exponent (α) were calculated from two-parameter nonlinear fits to the MSD versus time data (MSD = $4D_\alpha t^\alpha$), as described previously (Kneller et al., 2011). In the latter approach, anomalous diffusion is defined as $\alpha \neq 1$. Subdiffusion ($\alpha < 1$) has been documented in experimental and computational studies of lipid bilayers (Almeida and Vaz, 1995; Banks and Fradin, 2005; Feder et al., 1996; Jacobson et al., 1987).

The M2 protein consistently had an average diffusion constant lower than the other proteins (HA and NA; Figures 6 and 7), regardless of whether the MSD data were fitted to a normal or an anomalous diffusion model. The general order of diffusion coefficients was NA $\geq$ HA > M2. This may reflect a combination of the cross-sectional areas of the TM domains and the strength of their lipid interactions. M2 diffused very slowly (4.4×10^{-14} cm^2/s, as measured by atomic force microscopy) in a supported gel phase dipalmitoyl phophatidylcholine bilayer (Hughes et al., 2004). In addition to the simulations discussed above, we also performed simulations in a simplified model envelope containing only three lipid species and a lower cholesterol content (40%; Figure S7). In these lower cholesterol simulations the diffusion constant of M2 (for the normal diffusion model) was closer to that of HA than for other conditions. Furthermore, while the virion models exhibited subdiffusion (mean α ~0.8), the low (40%) cholesterol model exhibited approximately normal protein diffusion. The subdiffusive behavior of simple model transmembrane proteins embedded in a bilayer has previously been demonstrated using CG simulations (Schmidt and Weiss, 2011). Thus, the biologically realistic membrane lipid model leads to anomalous diffusion of the influenza virion proteins. As expected, temperature has an effect on diffusion coefficients—they are lower at room temperature (295 K) than at elevated temperature (323 K) (Figure S8). Perhaps less expected is the effect of the Forssman glycolipid, which substantially reduced protein diffusion coefficients.

If we take, e.g., HA in the FORS virion at 323 K, we obtain a mean value D of ~0.3×10^{-7} cm^2/s. In a recent study of membrane protein diffusion in crowded bilayers using CG simulations and a simple (PE/PG) lipid bilayer (Goose and Sansom, 2013), translational diffusion coefficients for individual bacterial outer membrane proteins (OMPs) varied between 0.5 and 3×10^{-7} cm^2/s, falling below 0.5×10^{-7} cm^2/s for crowded

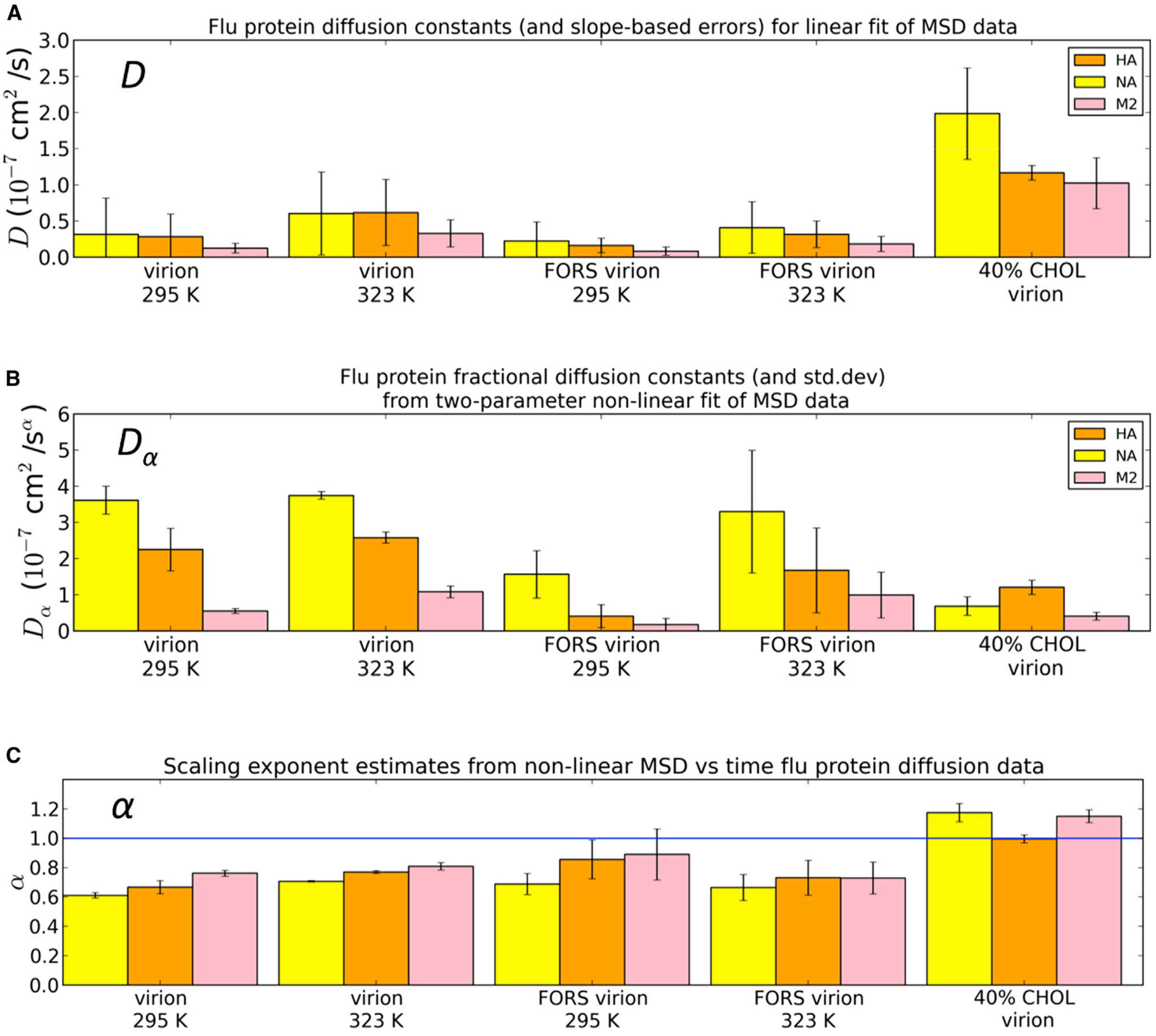

Figure 7. Protein Diffusion Coefficients

(A–C) Protein diffusion coefficients calculated for either (A) linear (MSD = $4Dt$) or (B) nonlinear (MSD = $4D_\alpha t^\alpha$) diffusion models. The scaling exponents (C), α, were also calculated for the nonlinear fit to the data, with values <1 consistent with anomalous subdiffusion and values $\cong$1 consistent with random-walk diffusion. SD values were extracted from two-parameter fits for the nonlinear analysis while uncertainties in the linear data were estimated as the difference between the slopes of the first and final halves of the MSD versus time data. See also Figure S9.

bilayers. Thus, the protein diffusion rates in the +FORS virion models are comparable with those in simple models of crowded lipid bilayers. Fluorescence recovery after photobleaching (FRAP) studies of crowded membrane proteins in reconstituted giant unilamellar vesicles (GUVs) revealed protein diffusion coefficients (0.4×10^{-7} cm^2/s) (Ramadurai et al., 2009) that match our simulation-based values for the +FORS virion. However, FRAP measurements of HA diffusion on the surfaces of infected cells yielded much lower D values in the range 10^{-10} to 10^{-9} cm^2/s (Shvartsman et al., 2003), possibly reflecting cytoskeletal interactions of lipid rafts. We previously reported diffusion coefficients of single CG HA molecules of $D \sim 10^{-7}$ cm^2/s in bilayer patches with 35% cholesterol and of $D \sim 0.3 \times 10^{-7}$ cm^2/s with no cholesterol (Parton et al., 2013). Thus, previously reported CG simulation diffusion constants are reasonably consistent with our current range of virion-based diffusion constants given the differences in protein concentration, lipid composition, and bilayer geometry.

The lipid (Figure 8; Figures S9 and S10) D values ranged from $\sim 0.2 \times 10^{-7}$ to $\sim 7 \times 10^{-7}$ cm^2/s. This range is consistent with that reported in a recent CG study of simple model systems (Goose and Sansom, 2013) discussed above, where phospholipid diffusion coefficients of $D = 8.5 \times 10^{-7}$ cm^2/s were observed in the absence of protein, falling to $\sim 4.0 \times 10^{-7}$ cm^2/s at a high protein concentration. We also note that FRAP studies of crowded GUVs yielded lipid diffusion

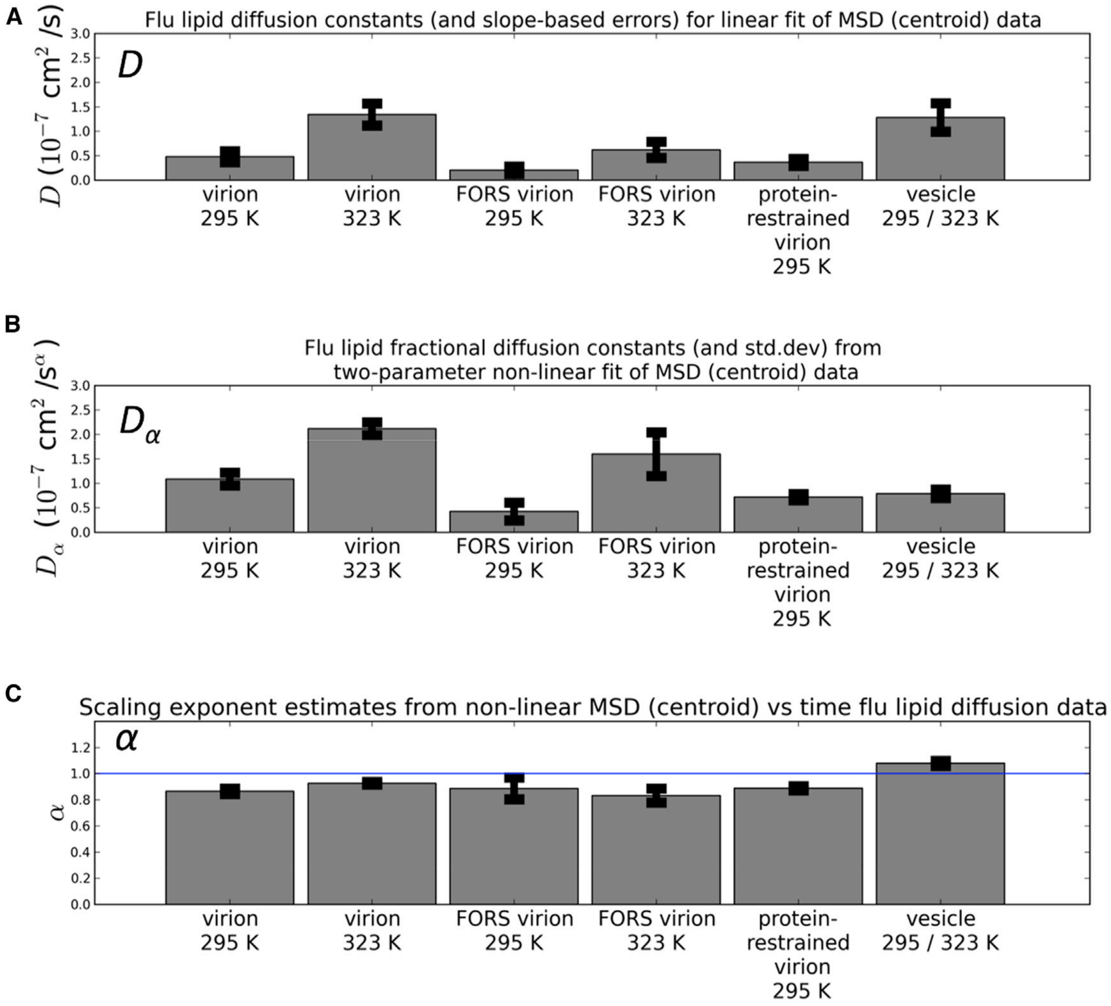

Figure 8. Lipid Diffusion Coefficients

(A–C) Lipid diffusion coefficients calculated for either (A) linear (MSD = $4Dt$) or (B) nonlinear (MSD = $4D_\alpha t^\alpha$) diffusion models and their reported uncertainties were averaged across all lipid species for a given simulation condition. The scaling exponents (C), α, were also calculated for the nonlinear fit to the data. The uncertainty metrics are as described in Figure 7, and the full diffusion analysis results for all lipid species in all simulation conditions is available in the Supporting Information. See also Figure S10.

coefficients of ~1 × 10^{-7} cm^2/s (Ramadurai et al., 2009), again consistent with our results. Solid-state NMR spectroscopy has been used to measure the average diffusion of reconstituted influenza lipid mixtures, with D ranging from 0.7 to 3.5 × 10^{-7} cm^2/s as temperature was varied from 290 to 310 K (Polozov et al., 2008). Thus, experimental and computational measurements of diffusion rates for comparable lipid compositions are in reasonable agreement, although given our observation of anomalous (subdiffusive) behavior (see below), one should exercise caution in making such comparisons. As expected, lipid diffusion constant values increase with temperature. At both temperatures, the presence of the Forssman glycolipid resulted in a substantial decrease in D values for all of the lipids present. In all cases, the Forssman glycolipid was on average the least mobile of the lipid species under similar conditions. Restraining the proteins (see above) also lowers all lipid diffusion coefficients. Overall, the lowest average lipid diffusion rates were for the virion including Forssman glycolipids at room temperature (295 K), as was the case for the proteins. Similar to the proteins, in most simulations the lipids exhibited subdiffusive ($\alpha < 1$) behavior, with on average $\alpha \sim 0.9$. FRAP studies of crowded GUVs demonstrated anomalous lipid diffusion ($\alpha = 0.9$) at higher degrees of membrane crowding (Ramadurai et al., 2009). Previous simulation results suggest that increasing protein concentration in membranes leads to the onset of anomalous diffusion (Javanainen et al., 2013), and α dropped from approximately 1 to 0.8 over the range of increasing protein concentration used in the recent CG study of a simple model discussed above (Goose and Sansom, 2013). Subdiffusion was not observed in the vesicle simulations.

Overall, we may conclude that the membrane of the influenza virus exhibits slow protein and lipid diffusion consistent with raft-like behavior. This is likely to contribute to the structural stability of the viral membrane in response to changes in environmental conditions.

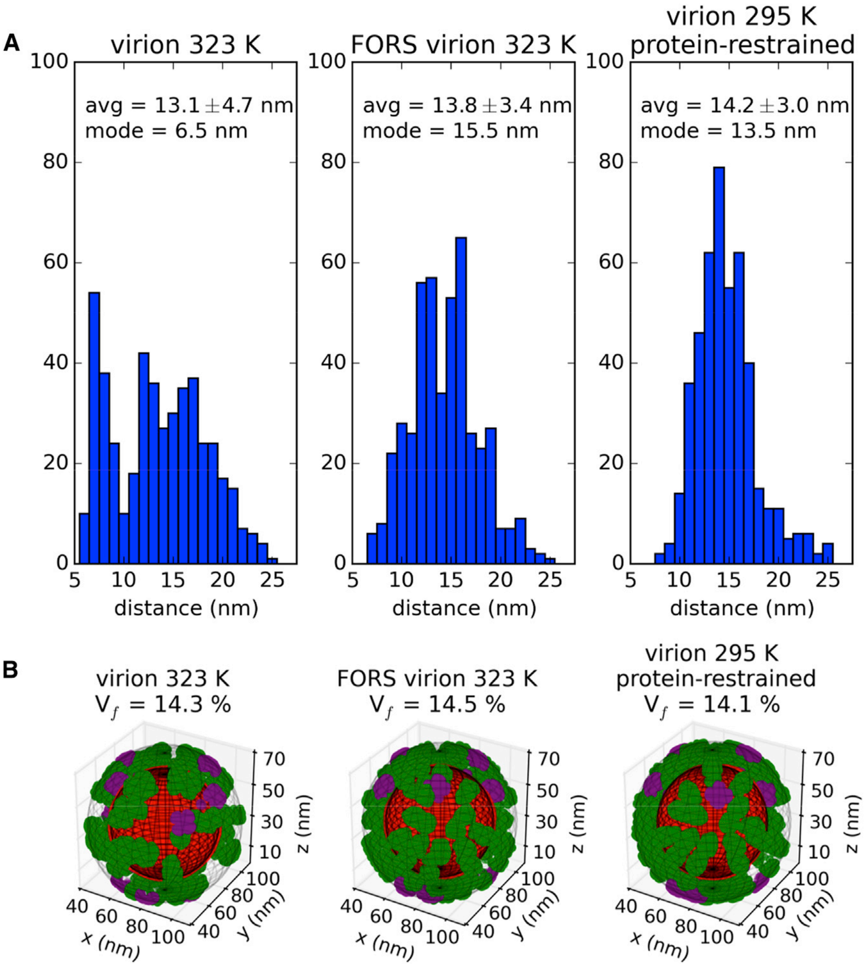

Figure 9. Spatial Disposition of Membrane Proteins

(A) Comparison of interprotein distance histograms in final snapshots of simulations (excluding the M2 proton channel). The centroids of each protein were employed and the closest five neighboring proteins were included for 460 distances.

(B) Assessment of influenza A spike glycoprotein fractional surface volume in final snapshots of production simulations. The phosphate particles in each virion (red) were used to define the inner boundary of the outer surface layer (contained within a diffuse meshgrid), while the outer boundary was assigned 13 nm farther from the virion centroid (contained within outermost diffuse meshgrid). The coordinates of particles representing the convex hulls of the spike glycoproteins are shown for HA (green) and NA (purple). The percentage of the outer surface layer volume (V_f) occupied by the spike glycoproteins is indicated above each condition. See also Figures S11–S15 and S19.

Protein and Lipid Domain Formation

Given the slow dynamics of the proteins and lipids, it is of interest to examine their spatial organization, as this offers the possibility of linking dynamic structural models through to cryo-tomographic images. The spacing (clustering) of proteins on the surface of the influenza A virion was previously studied by electron cryo-tomography (Harris et al., 2006; Wasilewski et al., 2012), providing useful information about the steric constraints of putative therapeutic binding sites. The average final-snapshot separation between the HA and NA spike proteins in our simulations ranges from 13.1 ± 4.7 nm to 14.2 ± 3.0 nm (Figure 9A; Figure S11), which agrees reasonably (i.e. within errors) with the experimental estimate of 11.2 ± 2.3 nm.

In addition, the simulations allow us to study the evolution of the interprotein spacing (clustering) behavior over time. The most striking change in clustering behavior was observed for the influenza A virion at 323 K (in the absence of the FORS glycolipid) with the average spacing changing over the 5 μs simulation from 14.2 ± 3.0 nm to 13.1 ± 4.7 nm, and with the mode of the distribution dropping drastically from 13.5 nm to 6.5 nm. This is consistent with formal clustering analysis for this simulation condition (Figure S12), where the number of protein clusters increases from 2 to 17. In contrast, in the presence of Forssman glycolipid there is less clustering of the spike proteins, with average final separation of 13.8 ± 3.4 nm, a mode of 15.5 nm, and no substantial evidence of clustering using a formal algorithm. The room temperature (295 K) simulations show similar behavior in the presence or absence of FORS, with final respective average separations of 13.9 ± 3.1 nm and 13.8 ± 3.8 nm. A decrease in interprotein separation to 13.0 ± 4.2 nm was observed for the simulation of the simpler (40% cholesterol) model. Thus, the glycolipid may reduce clustering of the HA and NA proteins, keeping them evenly distributed over the surface of the virion. Our results also suggest that the glycoprotein spikes of a spherical influenza virion may be suitably spaced to be bound by bivalent immunoglobulins G (IgGs), which have flexibly linked Fab domains that can extend 15 nm apart and crosslink adjacent spike glycoproteins (Wasilewski et al., 2012; Wrigley et al., 1983). The spacing between spike glycoproteins in our simulations is also sufficient to allow trivalent binding at the HA stem by the broadly neutralizing FI6 antibody (Wasilewski et al., 2012), which is effective against both group 1 and group 2 influenza A strains (Corti et al., 2011).

We also quantified the fractional volume of the outer virion surface, in a layer 13 nm thick, occupied by the spike proteins (Figure 9B; Figure S13). We obtained values between 14.1% and 15.0%, which are in good agreement with the experimental values reported for three X-31 virions (from 13.5% to 15.5%) (Wasilewski et al., 2012). Visualization of surface layer volumes occupied by proteins also reveals how the presence of the Forssman glycolipid prevents protein clustering, as discussed above.

The clustering of each lipid species was tracked over the course of the simulations, and the results were quantified using a formal clustering algorithm (Figures S14 and S15). The presence of the Forssman glycolipid appears to protect lipid

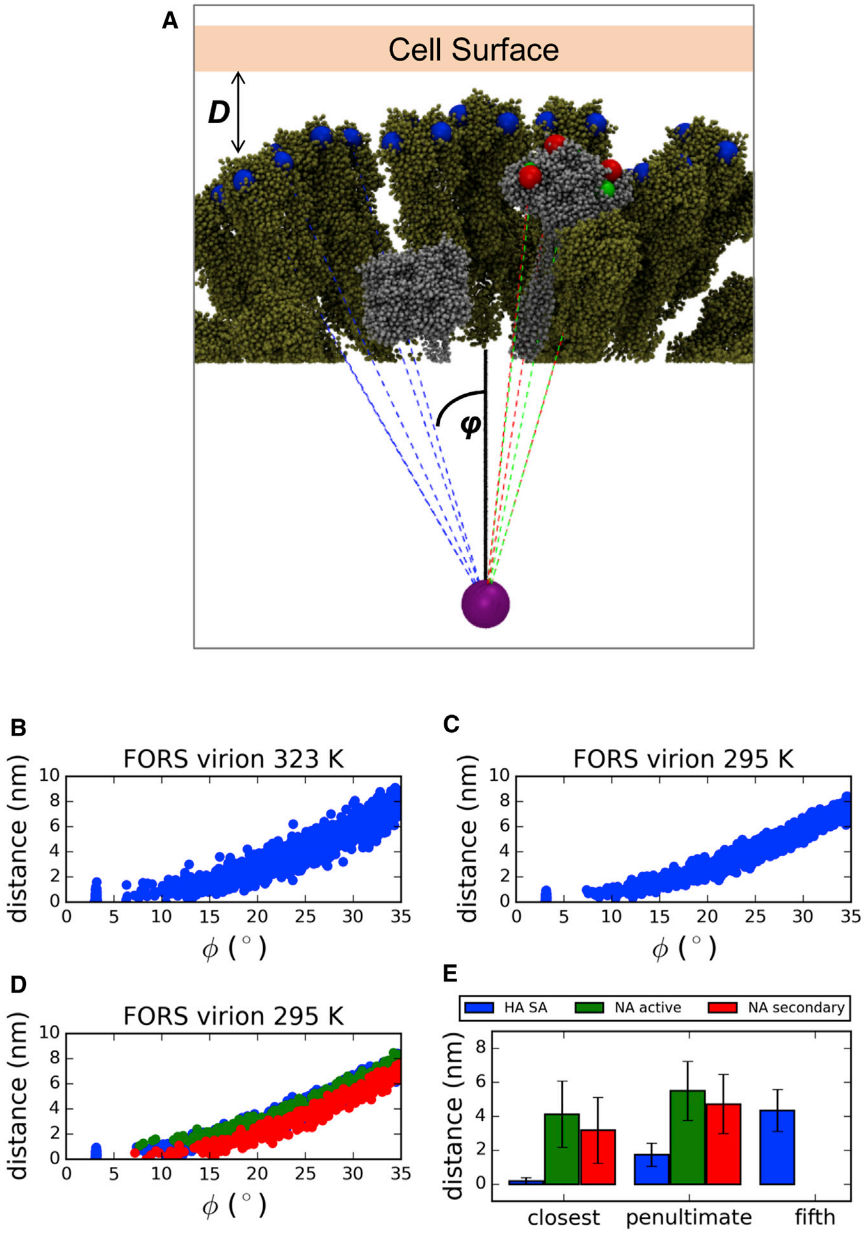

Figure 10. Geometric Constraints on the Binding of HA Trimers and NA Tetramers on the Approximately Spherical Influenza A Virion Surface at the End of the Simulations to Sialic Acid Receptors on an Idealized Host Cell Surface

(A) Schematic of SA receptor binding sites (blue) in HA trimers (tan), and of active site (green) and secondary SA receptor binding sites (red) in NA tetramers (silver) for 1/80 randomly chosen influenza A virion-host cell attack orientations with a single reference HA aligned along the +*Z* direction (solid black line). A subset of the binding site vectors (dashed lines) are shown and their angles (φ) are measured relative to the +*Z* reference axis. Putative binding sites are only shown if they fall within $\varphi \pm 35°$ of the virion centroid (purple sphere).

(B and C) The cumulative (all 80 attack orientations) HA binding site surface distances and their corresponding angles are plotted for the FORS-inclusive virion at both simulation temperatures.

(D and E) The matching results for SA binding sites on NA are also plotted for comparison for the FORS-inclusive virion at 295 K (D), with the closest, penultimate and fifth-closest average protein binding site distances compared (E), where available. For the FORS-inclusive virion at 295 K, the two closest HA trimers have average SA to binding site distances of 0.18 ± 0.20 nm and 1.73 ± 0.68 nm, respectively, which may be compared with closest HA and closest neighbor measured experimentally (Wasilewski et al., 2012). See also Figure S16.

clustering behaviors from temperature sensitivity. The minor populations of DOPE, DOPX, and POPS in the outer leaflet of the virion envelope exhibited six stable clusters each throughout the +FORS simulations at both temperatures. Conversely, in the absence of FORS at 323 K there were as many as 40 clusters at the end of the simulation for the corresponding lipid species.

Geometric Restrictions on Host Cell Sialic Acid Binding

In addition to the average spacing of glycoproteins discussed above, the curvature of the influenza A lipid envelope is also an important determinant of the accessibility of the binding sites on HA and the active and secondary binding sites on NA for the receptors (sialic acid [SA]) on the host cell surface. Given the weak (2–3 mM) in vitro binding affinity of HA for SA receptors (Gamblin and Skehel, 2010; Sauter et al., 1989), it is likely that infection requires multivalent binding (Wasilewski et al., 2012). Given the roughly spherical ultrastructure of our simulated influenza A virions, we assessed the distance of SA binding sites from a planar surface, as a first approximation to the target membrane in a host cell or in vitro assay, averaging across the set of 80 possible attack orientations where a single HA is aligned for direct contact with the surface (Figure 10). We report a range of average SA binding site to surface distances for the closest HA neighbor to the direct-contact HA trimer between 1.2 ± 0.9 nm to 1.7 ± 0.9 nm (with $\phi \sim 14°$) (Figure S16). The results are reasonably consistent with previously reported electron cryo-tomography measurements of influenza A virions placing the closest neighbor HA SA sites on average 1.2 nm from a planar surface for $\phi = 12°$ (Wasilewski et al., 2012), especially considering the filamentous morphology of the latter virions. Of course, one would expect a target cell membrane not to be exactly planar. Based on large-scale simulations of bilayers with a lipid composition approximating that of a mammalian cell membrane (Koldsø et al., 2014), one would expect spontaneous fluctuations of the local bilayer geometry to be of a magnitude (in terms of

radius of curvature) comparable to that of the dimensions of the virus particle, thus bringing substantially more SA residues within range of the HA molecules. Even if only 10% (n = 8) of the HA trimers in our model engaged host SA residues simultaneously the effective binding constant would depend on their product, yielding a much greater effective affinity. Our results also clearly demonstrate that NA is geometrically capable of competing with HA for interaction with SA beyond the site of direct contact, which may allow for SA cleavage by the NA active site (and virion budding) or additional binding of SA by the secondary site. The distinction between HA and NA was not possible in the above cryo-EM study, and we are now well positioned to examine low-virulence influenza strains where the NA stalk is shortened (Castrucci and Kawaoka, 1993; Durrant and Amaro, 2014).

Model Limitations

The CG simulation approach is an approximation compared to all-atom simulations (Freddolino et al., 2006). However, it allows for substantially longer simulation times, approaching those accessible by experimental biophysical measurements and permitting assessment of the dynamic behavior of proteins and lipids. Using virion models to assess avidity and multivalent interactions with host cells could benefit from multiscale or hybrid simulations (Ayton and Voth, 2009b; Gonzalez et al., 2013; Wassenaar et al., 2013) that combine both all-atom and CG representations, with atomistic representation utilized in the regions of binding interactions. A possible refinement of the CG simulation methodology would be to scale the system reference pressure by the volume ratio between the simulation box and internal RPO shell, coupling the internal and external pressure independently, as recently described (Louhivuori et al., 2010). However, as discussed below, refinements of the RPO shell model for the virion contents might also be considered.

Another limitation of our virion models is in the representation of the proteins. Experimental structures of the cytoplasmic tail, transmembrane domain, and stem domain of NA are not available, and had to be modeled (Parton, 2011). The model for HA also lacks high-resolution experimental structural information for the TMD, cytoplasmic domain, and a linker between the ectodomain and the TMD (Parton et al., 2013). A portion of the cytoplasmic tail and the acylations are missing from the model of M2 (Parton, 2011), which may influence protein-specific lipid recruitment (Figure S17). Although an X-ray structure for the M1 matrix protein is available (Harris et al., 2006), its ultrastructure remains controversial (Fontana and Steven, 2013).

The RNP core of the virion is not included in our simulations, being replaced by a sphere of suitably restrained particles. The RPO shell does not interact directly with the lipid envelope in our model. In vivo, the inner leaflet of the envelope appears to be lined by the M1 matrix protein, which may influence lipid dynamics. Other approaches could include the use of mobile anions to model the RNA (Larsson et al., 2012). Low-resolution cryo-EM structures of RNPs are available (Zheng and Tao, 2013), and a refined representation of the core of the virion could be included in the future. The current influenza A lipidome is based on virions propagated in canine (MDCK) cells (Gerl et al., 2012), and may need some refinement for consistency with proper human virions (Kijimoto-Ochiai et al., 1981; Yamamoto et al., 2012). It may also be important to include glycans in the CG models of the spike proteins given that, for example, glycosylation of HA may modulate virulence (Medina et al., 2013).

There is some recent evidence that lateral diffusion in CG lipid bilayer simulations may require milliseconds to converge in highly crowded membranes (Javanainen et al., 2013). These authors report reasonable convergence (using a similar force field to ours) of diffusion measured as a Gaussian distribution with α generally >0 within 1 μs in a system with 10-fold greater protein/lipid ratio than in the virion model. Averaging MSD values of multiple subtrajectories has previously been used to improve convergence of diffusion in CG lipid bilayer simulations (Goose and Sansom, 2013), and we have employed a similar strategy with the virions.

Conclusions

We have performed a number of CG resolution simulations of complete influenza virion membranes under a variety of conditions. Room temperature simulations with Forssman glycolipid present result in reduced mobility of bilayer species. The presence of protein restraints (to mimic interaction of spike proteins with M1 matrix) also restricts lipid mobility. We anticipate that reduced mobility in the virion membrane is likely to confer physical robustness to changes in environmental conditions. Our simulations also suggest that the viral membrane proteins do not promiscuously "clump together" and are competent for IgG antibody binding. There is a substantial public health interest in understanding the biophysical properties of the influenza A virion. Worldwide, influenza causes more than 250,000 deaths annually (http://www.who.int/mediacentre/factsheets/2003/fs211/en/), and the virion has been projected to persist in distilled water for longer than 3 years (Stallknecht et al., 1990). Furthermore, the virus can survive for extended periods (>12 days) in seawater (Mihai et al., 2011). Thus, we have produced a computational platform for probing the structural and biophysical stability of the virion in water. There are several other pleomorphic enveloped viruses that affect human health, including rubella virus, HIV, hantavirus, Ebola virus, rabies virus, and hepatitis C. The simulation and modeling studies described here may provide transferable approaches to studies of these other viruses.

EXPERIMENTAL PROCEDURES

Simulation Details

All vesicle/virion simulations were performed using GROMACS 4.5.5 (Hess et al., 2008) (http://www.gromacs.org) and the MARTINI 2.1 force field (Marrink et al., 2007; Monticelli et al., 2008), with all simulation times reported without correction factors. Default ionization states were assumed for amino acids and lipid headgroups. The force field was modified to include a "restrained shell particle" (RPO) which was attractive to water, while super-repulsive to other particles. RPO particles were always position-restrained in each dimension with a force constant of 10^3 kJ/mol/nm^2. The following lipid models were also included: ether-linked dioleoylphosphatidylethanolamine (DOPX), in which we modeled the ether linkage at the C1 position by changing particle 4 in DOPE from type Na to N0; and hydroxylated sphingomyelin (PPCH), in which we modeled the hydroxyl group of sphingomyelin (PPCS) particle 5 by changing this from particle type C1 to Nda. The Forssman glycolipid was parameterized in two stages. First, the ceramide backbone was based on the matching particles provided for sphingomyelin in the MARTINI force field.

Second, the headgroup of the Forssman glycolipid comprised the monosaccharides glucose, galactose, and *N*-acetylgalactosamine, with the glycosidic linkages as follows: GalNac-α(1-3)–GalNac-β(1-3)–Gal-α(1-4)–Gal-β(1-4)–Glc-β(1-1)–Cer. The initial monosaccharide parameters were based on the sweet MARTINI force field (Lopez et al., 2009) and previous in-house parameterization of the glycolipid GM3 (D.S., personal communication), and were refined by comparison with all-atom simulations using the GLYCAM force field (Kirschner et al., 2008). Further details of the CG Forssman glycolipid are provided in the Supplemental Information.

The HA, NA, and M2 proteins were modeled as described previously (Parton, 2011), and as described in more detail in the Supplemental Information (Figure S18). In brief, the model of HA was based on the X-ray structure Protein Data Bank (PDB) code 1MQM. This X-ray structure does not include the TM domain or the cytoplasmic domains, or a short linker between the ectodomain and TM domain. This missing region was modeled as an α helix. Palmitoyl chains were added to residues Cys555, Cys562, and Cys565 of the TM domain. The cytoplasmic domain was treated as unstructured. The NA stalk domain was modeled as a polyalanine coiled coil (based on a tetrameric coiled coil motif from the GCN4 leucine zipper protein, PDB code 1GCL, with its length (~10 nm) matched to cryo-EM images of the protein. The CG model of the M2 proton channel was derived directly from an NMR structure (PDB code 2RLF) comprising residues 18–60 of the M2 protein and including 15 residues of the C-terminal cytoplasmic tail as well as the α-helical TM domain.

Simulations employed 10 fs time steps, and coordinates were saved every 10^4 steps (every 0.1 ns). Coulomb and van der Waals interactions were respectively shifted off between 0.0 and 1.2 nm and 0.9 and 1.2 nm. Acylated proteins, lipids, RPO particles, and solvent were separately temperature coupled using the Berendsen algorithm (Berendsen et al., 1984) with a 1.0 ps time constant. Isotropic pressure coupling was performed using the Berendsen algorithm with a 1.1 ps time constant, 1×10^{-6}/bar compressibility, and a 1.0 bar reference pressure. In the case where all 107 viral membrane proteins were restrained, each of the proteins was subject to center of mass pulling (in all three dimensions) using an umbrella potential in GROMACS. The pull force (harmonic force constant, $k = 10^5$ kJ/mol/nm^2) was exerted in the direction of an absolute reference point at the origin, with initial pull vector set to (0,0,0) and pull rate set to 0 nm/ps (immobilized reference) for each protein. Forces and center of mass values from the pull code were written every 10^4 steps (every 0.1 ns). All systems were neutralized with Na^+ ions (Figure S3).

SUPPLEMENTAL INFORMATION

Supplemental Information includes 19 figures, Supplemental Computational Procedures, and Supplemental Information Files S20–S24 and can be found with this article online at http://dx.doi.org/10.1016/j.str.2014.12.019.

AUTHOR CONTRIBUTIONS

T.R. benchmarked, set up, performed, and analyzed simulations. D.P. developed initial viral models and simulations. D.S. provided glycolipid parameters, and E.J. and P.W.F. provided additional methodologies. T.R. and M.C. produced visualizations. T.R., M.B., and M.S.P.S. designed the research project. T.R., P.W.F., and M.S.P.S. wrote the manuscript.

ACKNOWLEDGMENTS

We thank Drs. Phillip J. Stansfeld, Joseph Goose, Antreas Kalli, Heidi Koldsø, Maria Musgaard, Sarah Rouse, Greg Ross, and Jean Helie (University of Oxford) for helpful advice. The members of the Baaden laboratory at IBPC (Paris, France) and the Mathematical Institute (Oxford), as well as Dr. Joe Pitt-Francis (Computer Science, Oxford) provided helpful advice. Computational resources were primarily provided by a PRACE grant to T.R. and M.S.P.S. Part of this work was performed using HPC resources from GENCI-CINES/IDRIS (Grant 2012-071714). Additional computing resources were provided by an HPC-Europa2 grant to T.R. (Jade supercomputer, Montpellier, France), the Baaden lab, and ACEnet. T.R. acknowledges the Canadian Institutes of Health Research (CIHR) for postdoctoral funding and Somerville College (Oxford) for a Fulford Junior Research Fellowship. Research in M.S.P.S.'s group is supported by grants from the Wellcome Trust and the BBSRC.

Received: October 28, 2014
Revised: December 9, 2014
Accepted: December 17, 2014
Published: February 19, 2015

REFERENCES

Almeida, P.F.F., and Vaz, W.L.C. (1995). Lateral diffusion in membranes. In Structure and Dynamics of Membranes: From Cells to Vesicles, *Vol. 1*, R. Lipowsky and E. Sackmann, eds. Handbook of Biological Physics (North-Holland), pp. 305–357.

Ayton, G.S., and Voth, G.A. (2009a). Hybrid coarse-graining approach for lipid bilayers at large length and time scales. J. Phys. Chem. B *113*, 4413–4424.

Ayton, G.S., and Voth, G.A. (2009b). Systematic multiscale simulation of membranes protein systems. Curr. Opin. Struct. Biol. *19*, 138–144.

Ayton, G.S., and Voth, G.A. (2010). Multiscale computer simulation of the immature HIV-1 virion. Biophys. J. *99*, 2757–2765.

Banks, D.S., and Fradin, C. (2005). Anomalous diffusion of proteins due to molecular crowding. Biophys. J. *89*, 2960–2971.

Berendsen, H.J.C., Postma, J.P.M., van Gunsteren, W.F., DiNola, A., and Haak, J.R. (1984). Molecular dynamics with coupling to an external bath. J. Chem. Phys. *81*, 3684–3690.

Bittman, R., Majuk, Z., Honig, D.S., Compans, R.W., and Lenard, J. (1976). Permeability properties of membrane of vesicular stomatitis virions. Biochim. Biophys. Acta *433*, 63–74.

Calder, L.J., Wasilewski, S., Berriman, J.A., and Rosenthal, P.B. (2010). Structural organization of a filamentous influenza A virus. Proc. Natl. Acad. Sci. USA *107*, 10685–10690.

Castrucci, M.R., and Kawaoka, Y. (1993). Biologic importance of neuraminidase stalk length in influenza A virus. J. Virol. *67*, 759–764.

Corti, D., Voss, J., Gamblin, S.J., Codoni, G., Macagno, A., Jarrossay, D., Vachieri, S.G., Pinna, D., Minola, A., Vanzetta, F., et al. (2011). A neutralizing antibody selected from plasma cells that binds to group 1 and group 2 influenza A hemagglutinins. Science *333*, 850–856.

Dupuy, A.D., and Engelman, D.M. (2008). Protein area occupancy at the center of the red blood cell membrane. Proc. Natl. Acad. Sci. USA *105*, 2848–2852.

Durrant, J.D., and Amaro, R.E. (2014). Lipidwrapper: an algorithm for generating large-scale membrane models of arbitrary geometry. PLoS Comp. Biol. *10*, e1003720.

Feder, T.J., BrustMascher, I., Slattery, J.P., Baird, B., and Webb, W.W. (1996). Constrained diffusion or immobile fraction on cell surfaces: A new interpretation. Biophys. J. *70*, 2767–2773.

Fontana, J., and Steven, A.C. (2013). At low pH, influenza virus matrix protein m1 undergoes a conformational change prior to dissociating from the membrane. J. Virol. *87*, 5621–5628.

Freddolino, P.L., Arkhipov, A.S., Larson, S.B., McPherson, A., and Schulten, K. (2006). Molecular dynamics simulations of the complete satellite tobacco mosaic virus. Structure *14*, 437–449.

Gamblin, S.J., and Skehel, J.J. (2010). Influenza hemagglutinin and neuraminidase membrane glycoproteins. J. Biol. Chem. *285*, 28403–28409.

Gambin, Y., Lopez-Esparza, R., Reffay, M., Sierecki, E., Gov, N.S., Genest, M., Hodges, R.S., and Urbach, W. (2006). Lateral mobility of proteins in liquid membranes revisited. Proc. Natl. Acad. Sci. USA *103*, 2098–2102.

Gerl, M.J., Sampaio, J.L., Urban, S., Kalvodova, L., Verbavatz, J.M., Binnington, B., Lindemann, D., Lingwood, C.A., Shevchenko, A., Schroeder, C., and Simons, K. (2012). Quantitative analysis of the lipidomes of the influenza virus envelope and MDCK cell apical membrane. J. Cell Biol. *196*, 213–221.

Gonzalez, H.C., Darre, L., and Pantano, S. (2013). Transferable mixing of atomistic and coarse-grained water models. J. Phys. Chem. B *117*, 14438–14448.

Goose, J.E., and Sansom, M.S.P. (2013). Reduced lateral mobility of lipids and proteins in crowded membranes. PLoS Comp. Biol. *9*, e1003033.

Gu, R.-X., Liu, L.A., and Wei, D.-Q. (2013). Structural and energetic analysis of drug inhibition of the influenza A M2 proton channel. Trends Pharmacol. Sci. *34*, 571–580.

Ha, Y., Stevens, D.J., Skehel, J.J., and Wiley, D.C. (2003). X-ray structure of the hemagglutinin of a potential H3 avian progenitor of the 1968 Hong Kong pandemic influenza virus. Virology *309*, 209–218.

Harris, A., Cardone, G., Winkler, D.C., Heymann, J.B., Brecher, M., White, J.M., and Steven, A.C. (2006). Influenza virus pleiomorphy characterized by cryoelectron tomography. Proc. Natl. Acad. Sci. USA *103*, 19123–19127.

Hess, B., Kutzner, C., van der Spoel, D., and Lindahl, E. (2008). GROMACS 4: algorithms for highly efficient, load-balanced, and scalable molecular simulation. J. Chem. Theor. Comp. *4*, 435–447.

Hughes, T., Strongin, B., Gao, F.P., Vijayvergiya, V., Busath, D.D., and Davis, R.C. (2004). AFM visualization of mobile influenza A M2 molecules in planar bilayers. Biophys. J. *87*, 311–322.

Jacobson, K., Ishihara, A., and Inman, R. (1987). Lateral diffusion of proteins in membranes. Ann. Rev. Physiol. *49*, 163–175.

Javanainen, M., Hammaren, H., Monticelli, L., Jeon, J.-H., Miettinen, M.S., Martinez-Seara, H., Metzler, R., and Vattulainen, I. (2013). Anomalous and normal diffusion of proteins and lipids in crowded lipid membranes. Faraday Discuss. *161*, 397–417.

Kijimoto-Ochiai, S., Takahashi, W., and Makita, A. (1981). Anti-Forssman antibody in human-sera—properties and decreased level in cancer patients. Jpn. J. Exp. Med. *51*, 149–155.

Kirschner, K.N., Yongye, A.B., Tschampel, S.M., Gonzalez-Outeirino, J., Daniels, C.R., Foley, B.L., and Woods, R.J. (2008). GLYCAM06: a generalizable biomolecular force field. Carbohydrates. J. Comp. Chem. *29*, 622–655.

Kneller, G.R., Baczynski, K., and Pasenkiewicz-Gierula, M. (2011). Consistent picture of lateral subdiffusion in lipid bilayers: molecular dynamics simulation and exact results. J. Chem. Phys. *135*, 3651800.

Koldsø, H., Shorthouse, D., Hélie, J., and Sansom, M.S.P. (2014). Lipid clustering correlates with membrane curvature as revealed by molecular simulations of complex lipid bilayers. PLoS Comp. Biol. *10*, e1003911.

Larsson, D.S.D., Liljas, L., and van der Spoel, D. (2012). Virus capsid dissolution studied by microsecond molecular dynamics simulations. PLoS Comp. Biol. *8*, e1002502.

Lopez, C.A., Rzepiela, A.J., de Vries, A.H., Dijkhuizen, L., Huenenberger, P.H., and Marrink, S.J. (2009). Martini coarse-grained force field: extension to carbohydrates. J. Chem. Theor. Comput. *5*, 3195–3210.

Louhivuori, M., Risselada, H.J., van der Giessen, E., and Marrink, S.J. (2010). Release of content through mechano-sensitive gates in pressurized liposomes. Proc. Natl. Acad. Sci. USA *107*, 19856–19860.

Marrink, S.J., and Mark, A.E. (2003). Molecular dynamics simulation of the formation, structure, and dynamics of small phospholipid vesicles. J. Am. Chem. Soc. *125*, 15233–15242.

Marrink, S.J., and Tieleman, D.P. (2013). Perspective on the Martini model. Chem. Soc. Rev. *42*, 6801–6822.

Marrink, S.J., Risselada, J., Yefimov, S., Tieleman, D.P., and de Vries, A.H. (2007). The MARTINI forcefield: coarse grained model for biomolecular simulations. J. Phys. Chem. B *111*, 7812–7824.

Medina, R.A., Stertz, S., Manicassamy, B., Zimmermann, P., Sun, X.J., Albrecht, R.A., Uusi-Kerttula, H., Zagordi, O., Belshe, R.B., Frey, S.E., et al. (2013). Glycosylations in the globular head of the hemagglutinin protein modulate the virulence and antigenic properties of the H1N1 influenza viruses. Sci. Transl. Med. *5*, 187ra170.

Mihai, M.E., Tecu, C., Ivanciuc, A.E., Necula, G., Lupulescu, E., and Onu, A. (2011). Survival of H5N1 influenza virus in water and its inactivation by chemical methods. Roum. Arch. Microbiol. Immunol. *70*, 78–84.

Monticelli, L., Kandasamy, S.K., Periole, X., Larson, R.G., Tieleman, D.P., and Marrink, S.J. (2008). The MARTINI coarse grained force field: extension to proteins. J. Chem. Theor. Comput. *4*, 819–834.

Parton, D. (2011). Pushing the boundaries: molecular dynamics simulations of complex biological membranes, DPhil thesis. (Oxford: University of Oxford).

Parton, D.L., Tek, A., Baaden, M., and Sansom, M.S.P. (2013). Formation of raft-like assemblies within clusters of influenza hemagglutinin observed by MD simulations. PLoS Comp. Biol. *9*, e1003034.

Polozov, I.V., Bezrukov, L., Gawrisch, K., and Zimmerberg, J. (2008). Progressive ordering with decreasing temperature of the phospholipids of influenza virus. Nat. Chem. Biol. *4*, 248–255.

Ramadurai, S., Holt, A., Krasnikov, V., van den Bogaart, G., Killian, J.A., and Poolman, B. (2009). Lateral diffusion of membrane proteins. J. Am. Chem. Soc. *131*, 12650–12656.

Risselada, H.J., Marelli, G., Fuhrmans, M., Smirnova, Y.G., Grubmuller, H., Marrink, S.J., and Muller, M. (2012). Line-tension controlled mechanism for influenza fusion. PLoS One *7*, e38302.

Sauter, N.K., Bednarski, M.D., Wurzburg, B.A., Hanson, J.E., Whitesides, G.M., Skehel, J.J., and Wiley, D.C. (1989). Hemagglutinins from 2 influenza-virus variants bind to sialic-acid derivatives with millimolar dissociation-constants—a 500-MHz proton nuclear magnetic-resonance study. Biochemistry *28*, 8388–8396.

Schmidt, U., and Weiss, M. (2011). Anomalous diffusion of oligomerized transmembrane proteins. J. Chem. Phys. *134*, 165101.

Schmitt, A.P., and Lamb, R.A. (2005). Influenza virus assembly and budding at the viral budozone. In Virus Structure and Assembly, *Vol. 64*, P. Roy, ed.. Advances in Virus Research (Academic Press), pp. 383–416.

Schnell, J.R., and Chou, J.J. (2008). Structure and mechanism of the M2 proton channel of influenza A virus. Nature *451*, 591–595.

Shigematsu, S., Dublineau, A., Sawoo, O., Batejat, C., Matsuyama, T., Leclercq, I., and Manuguerra, J.C. (2014). Influenza A virus survival in water is influenced by the origin species of the host cell. Influenza Other Respir. Viruses *8*, 123–130.

Shvartsman, D.E., Kotler, M., Tall, R.D., Roth, M.G., and Henis, Y.I. (2003). Differently anchored influenza hemagglutinin mutants display distinct interaction dynamics with mutual rafts. J. Cell Biol. *163*, 879–888.

Stallknecht, D.E., Shane, S.M., Kearney, M.T., and Zwank, P.J. (1990). Persistence of avian influenza viruses in water. Avian Dis. *34*, 406–411.

Stanley, P., and Cummings, R.D. (2009). Structures common to different glycans. In Essentials of Glycobiology, A. Varki, R.D. Cummings, J.D. Esko, H.H. Freeze, P. Stanley, C.R. Bertozzi, G.W. Hart, and M.E. Etzler, eds. (Cold Spring Harbor Laboratory Press).

Stansfeld, P.J., and Sansom, M.S.P. (2011). Molecular simulation approaches to membrane proteins. Structure *19*, 1562–1572.

Takeda, M., Pekosz, A., Shuck, K., Pinto, L.H., and Lamb, R.A. (2002). Influenza A virus M-2 ion channel activity is essential for efficient replication in tissue culture. J. Virol. *76*, 1391–1399.

Varghese, J.N., and Colman, P.M. (1991). 3-Dimensional structure of the neuraminidase of influenza virus-A/Tokyo/3/67 at 2.2 Å resolution. J. Mol. Biol. *221*, 473–486.

Veit, M., and Thaa, B. (2011). Association of influenza virus proteins with membrane rafts. Adv. Virol. *2011*, 370606.

Wadell, H. (1935). Volume, shape, and roundness of quartz particles. J. Geol. *43*, 250–280.

Wang, X., Xu, F.T., Liu, J.S., Gao, B.Q., Liu, Y.X., Zhai, Y.J., Ma, J., Zhang, K., Baker, T.S., Schulten, K., et al. (2013). Atomic model of rabbit hemorrhagic disease virus by cryo-electron microscopy and crystallography. PLoS Pathog. *9*, e1003132.

Wasilewski, S., Calder, L.J., Grant, T., and Rosenthal, P.B. (2012). Distribution of surface glycoproteins on influenza A virus determined by electron cryotomography. Vaccine *30*, 7368–7373.

Wassenaar, T.A., Ingolfsson, H.I., Priess, M., Marrink, S.J., and Schafer, L.V. (2013). Mixing MARTINI: electrostatic coupling in hybrid atomistic-coarse-grained biomolecular simulations. J. Phys. Chem. B *117*, 3516–3530.

Wrigley, N.G., Brown, E.B., Daniels, R.S., Douglas, A.R., Skehel, J.J., and Wiley, D.C. (1983). Electron-microscopy of influenza hemagglutinin-monoclonal antibody complexes. Virology *131*, 308–314.

Yamamoto, M., Cid, E., and Yamamoto, F. (2012). Molecular genetic basis of the human Forssman glycolipid antigen negativity. Sci. Rep. *2*, 975.

Zhao, G., Perilla, J.R., Yufenyuy, E.L., Meng, X., Chen, B., Ning, J., Ahn, J., Gronenborn, A.M., Schulten, K., Aiken, C., and Zhang, P. (2013). Mature HIV-1 capsid structure by cryo-electron microscopy and all-atom molecular dynamics. Nature *497*, 643–646.

Zheng, W., and Tao, Y.J. (2013). Structure and assembly of the influenza A virus ribonucleoprotein complex. FEBS Lett. *587*, 1206–1214.

Zink, M., and Grubmüller, H. (2009). Mechanical properties of the icosahedral shell of Southern Bean Mosaic Virus: a molecular dynamics study. Biophys. J. *96*, 1350–1363.

Zink, M., and Grubmüller, H. (2010). Primary changes of the mechanical properties of Southern Bean Mosaic Virus upon calcium removal. Biophys. J. *98*, 687–695.

Structure, Volume *23*

Supplemental Information

Nothing to Sneeze At: A Dynamic and Integrative Computational Model of an Influenza A Virion

Tyler Reddy, David Shorthouse, Daniel L. Parton, Elizabeth Jefferys, Philip W. Fowler, Matthieu Chavent, Marc Baaden, and Mark S.P. Sansom

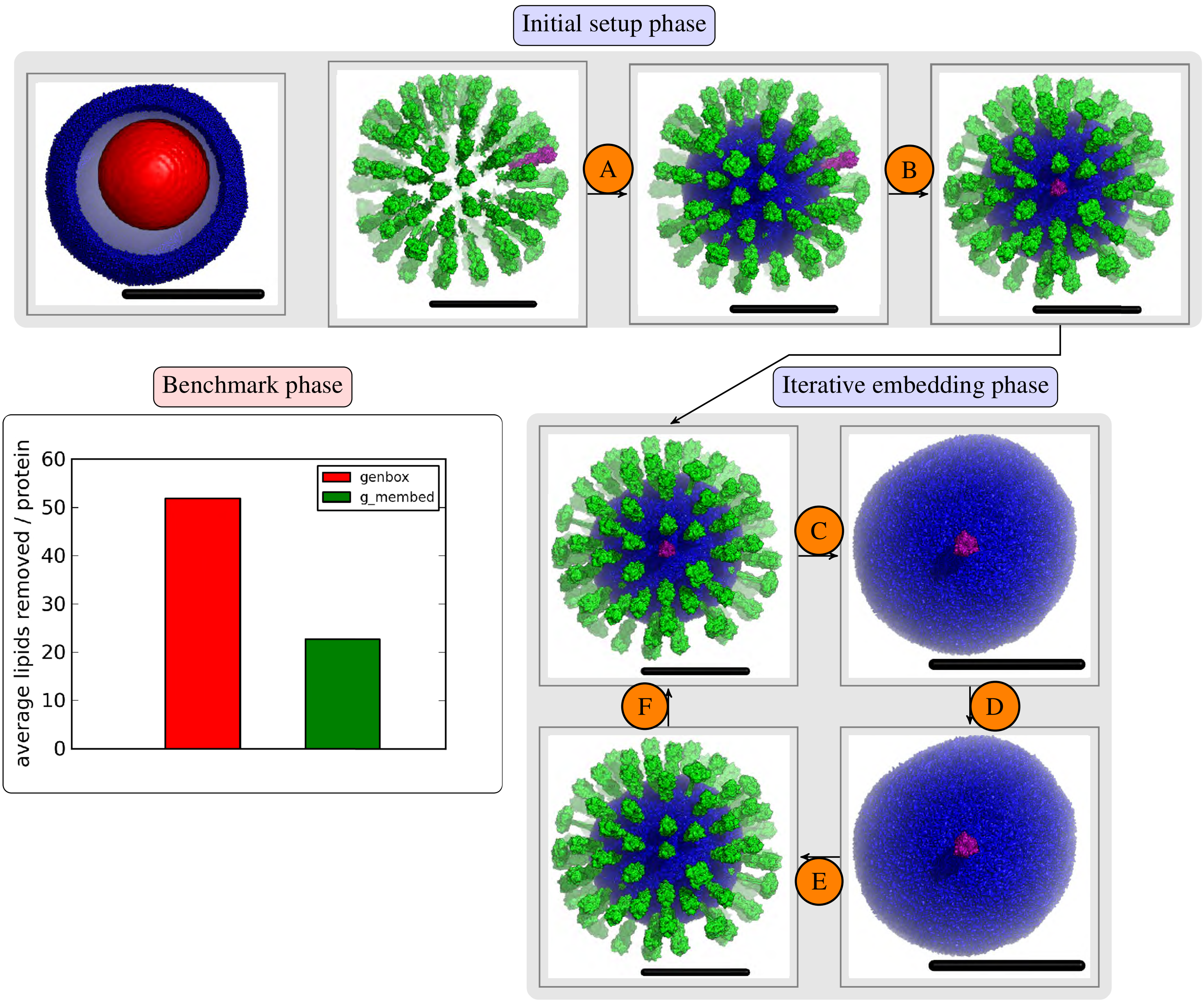

Figure S1: (Related to Figure 1) Procedure for embedding viral proteins into equilibrated vesicle. Lipids are shown in blue and RPO core in red, with a 40 nm scale bar shown in black. The protein membrane-embedding candidate (an HA trimer) is shown in purple and the other proteins in green. In part (A), the equilibrated vesicle and RPO coordinates are combined with the protein coordinates directly by superposition. In part (B), the system is translated to place the centroid of the RPO core at the origin and aligned such that the principal axis of the protein-embedding candidate is along the Z-axis. In part (C), only the protein embedding candidate and already-embedded proteins are retained prior to g_membed, with all other superposed proteins stripped from the system. In part (D), the g_membed code is used to embed the candidate protein by shrinking to 0.1 fractional xy size and expanding to full size in 1000 steps (0.8 nm probe radius, NVT). In part (E), stripped protein coordinates are reintegrated to their superposed positions. Finally, in part (F), the system is rotated to place the next protein membrane-embedding candidate along the Z-axis, unless the loop has completed and all proteins have been embedded. Benchmarking results compare the number of lipids removed per protein using this approach and a crude solvation-based approach reported previously (Parton, 2011).

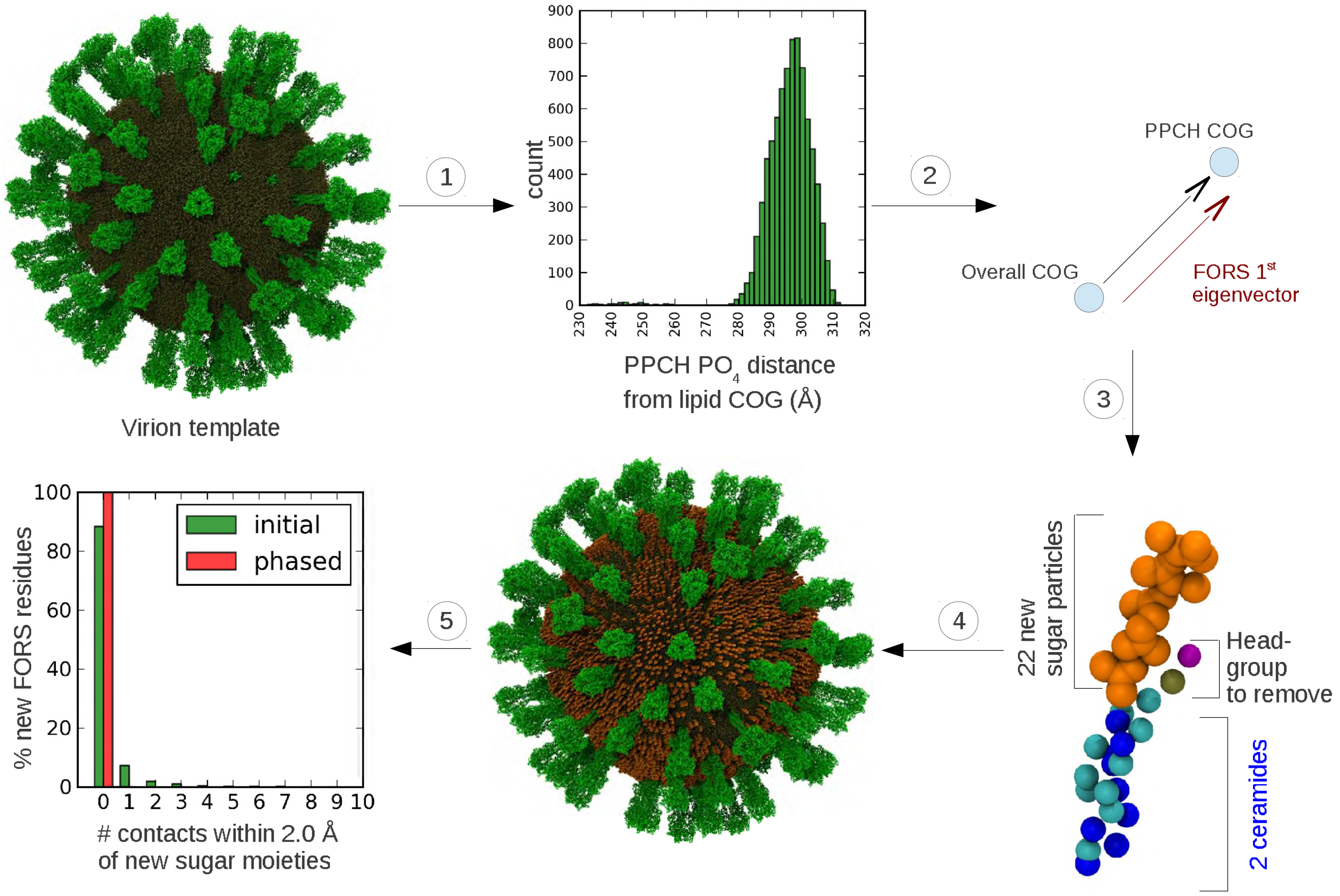

Figure S2: (Related to Figure 1) Producing an influenza virion with Forssman glycolipid by computational mutagenesis. The initial configuration consists of a virion template with 7931 sphingolipids (hydroxylated sphingomyelin residues, PPCH). An assessment of PPCH headgroup distance from the lipid centroid of the system allows identification of the large majority of PPCH residues residing in the outer leaflet (*step 1*). A random subset (68 % to match lipidome (Gerl et al., 2012)) of outer leaflet PPCH residues (> 280 Å threshold) was used to generate vectors connecting the overall lipid centroid of the system to their lipid residue centroid. The first eigenvector of a new FORS glycolipid molecule was aligned to each of the latter vectors to ensure that substitution-candidate FORS glycolipids preserved the bilayer orientation of the original PPCH residues (*step 2*). The new FORS molecules were then translated such that the centroid of their last 10 particles matched the centroid of the last 10 particles of the original PPCH (*step 3*). With an approximate overlap of ceramide tails for the original and substitution-candidate sphingolipids, the original ceramide was preserved (to avert introduction of steric conflicts) while the PPCH headgroup was replaced with the FORS glycan (*step 4*). The 5337 new sugar moieties (*orange*) initially exhibit steric conflicts within a 2.0 Å threshold and were therefore progressively incorporated to the system by adjustment of the GROMACS soft core potential, which resolved steric conflicts for 99.96 % of the FORS sugar moieties (*step 5*).

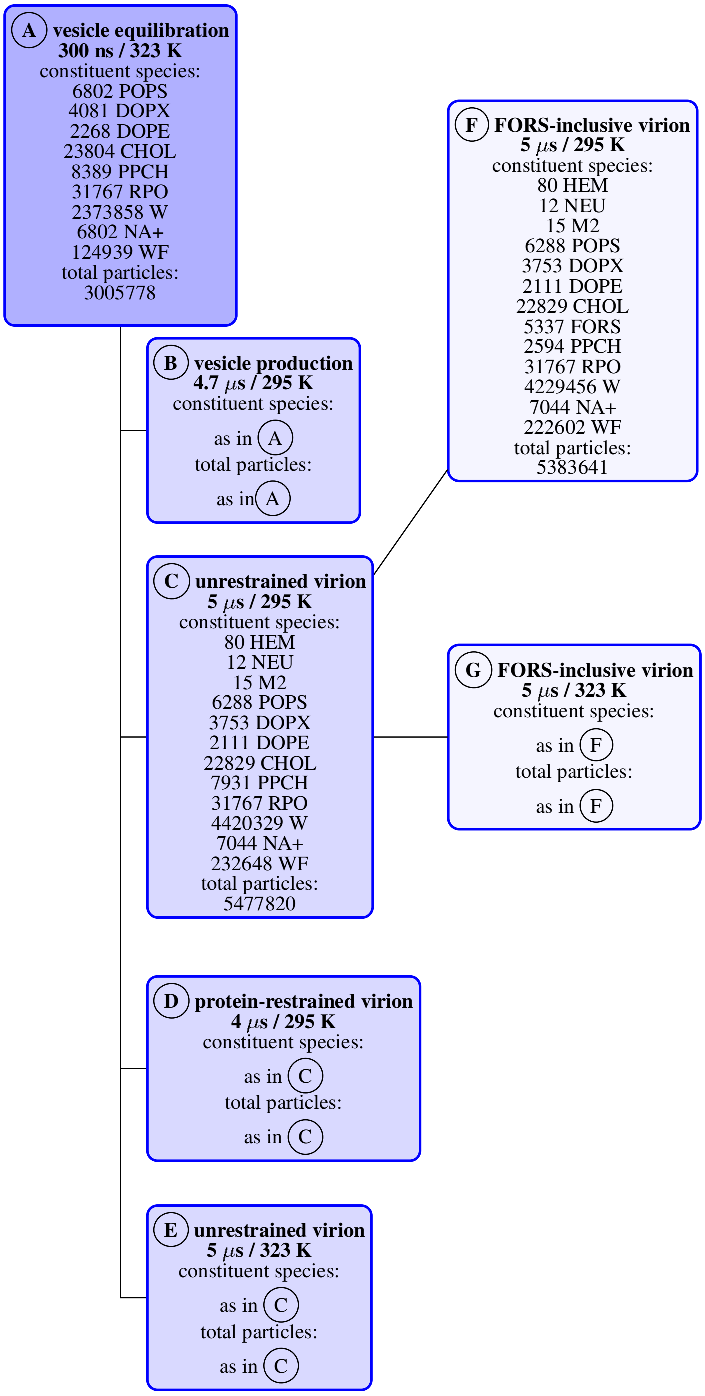

Figure S3: (Related to Figures 1,3) Flow chart summary of the construction and interdependence of vesicle and virion simulation constructs of influenza A. Substantially more detailed than version in the manuscript proper.

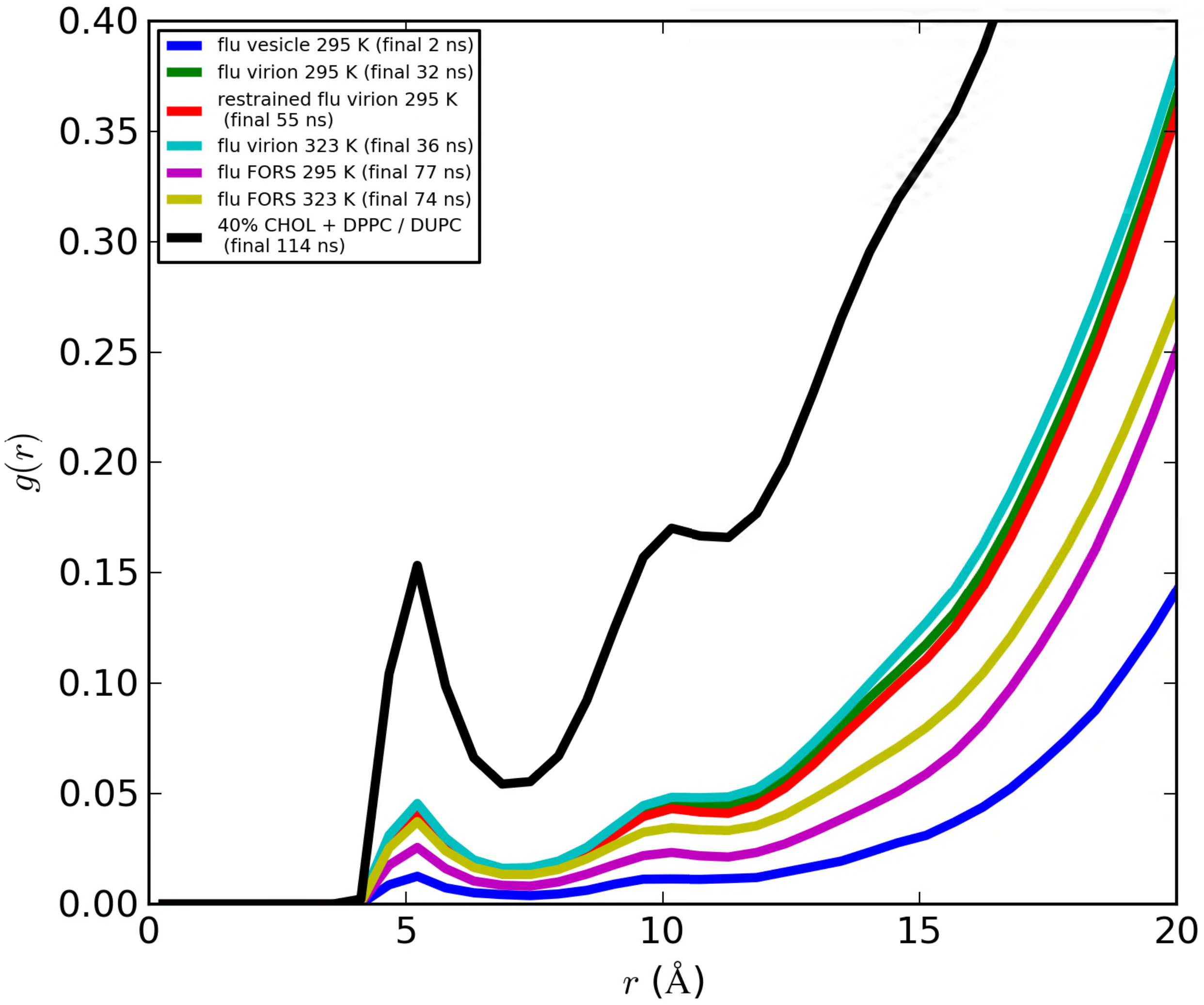

Figure S4: (Related to Figure 2) RDFs between influenza lipid tail particles and solvent particles.

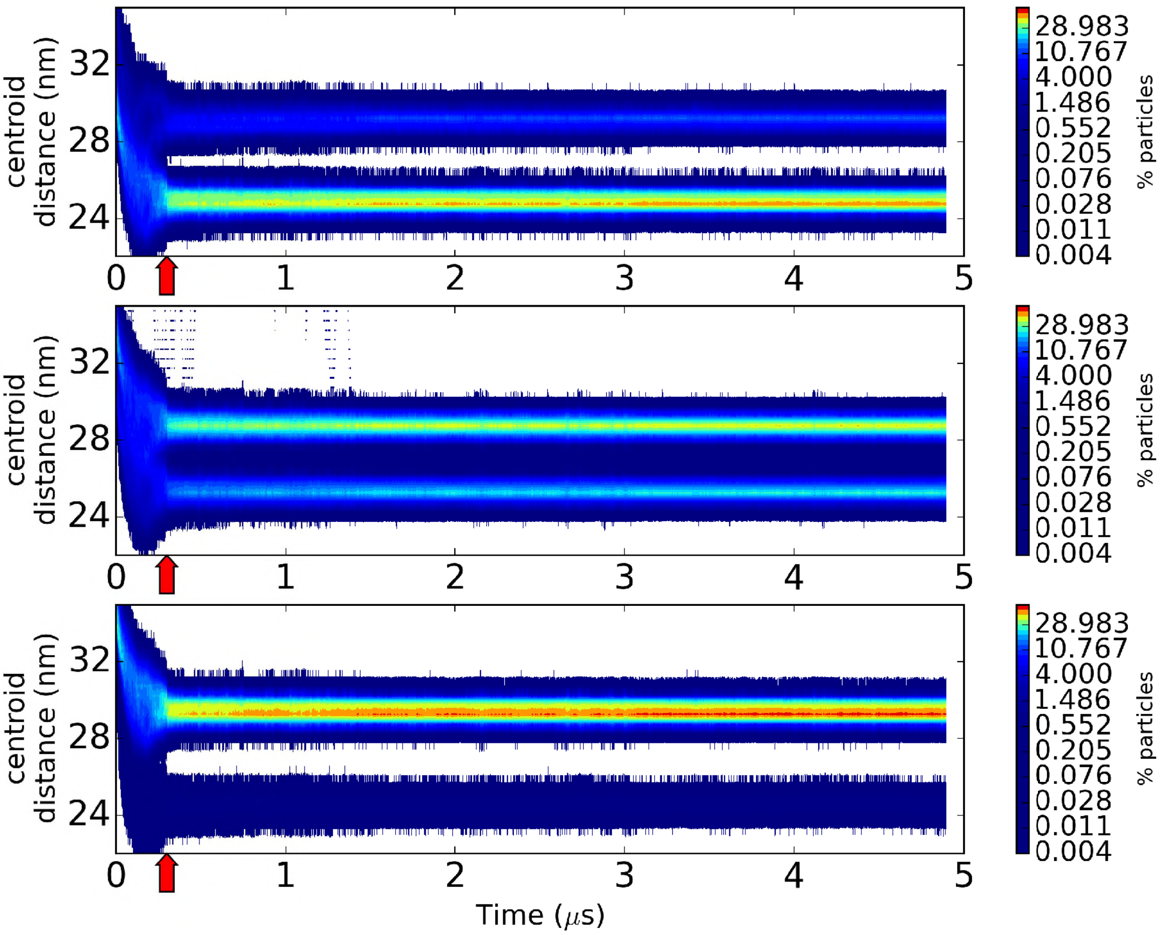

Figure S5: (Related to Figure 2) Tracking the leaflet distribution of viral lipids in a vesicle. Contour plots of the distance between lipid species and the vesicle centroid are shown for representative inner leaflet species (POPS, *top*), central species (CHOL, *middle*), and outer leaflet species (PPCH, *bottom*). Phosphate particles were used for POPS and PPCH, while the ROH group was used for calculations with CHOL. The red arrows indicate the time (0.3 μs) where the temperature was transitioned from 323 K to 295 K.

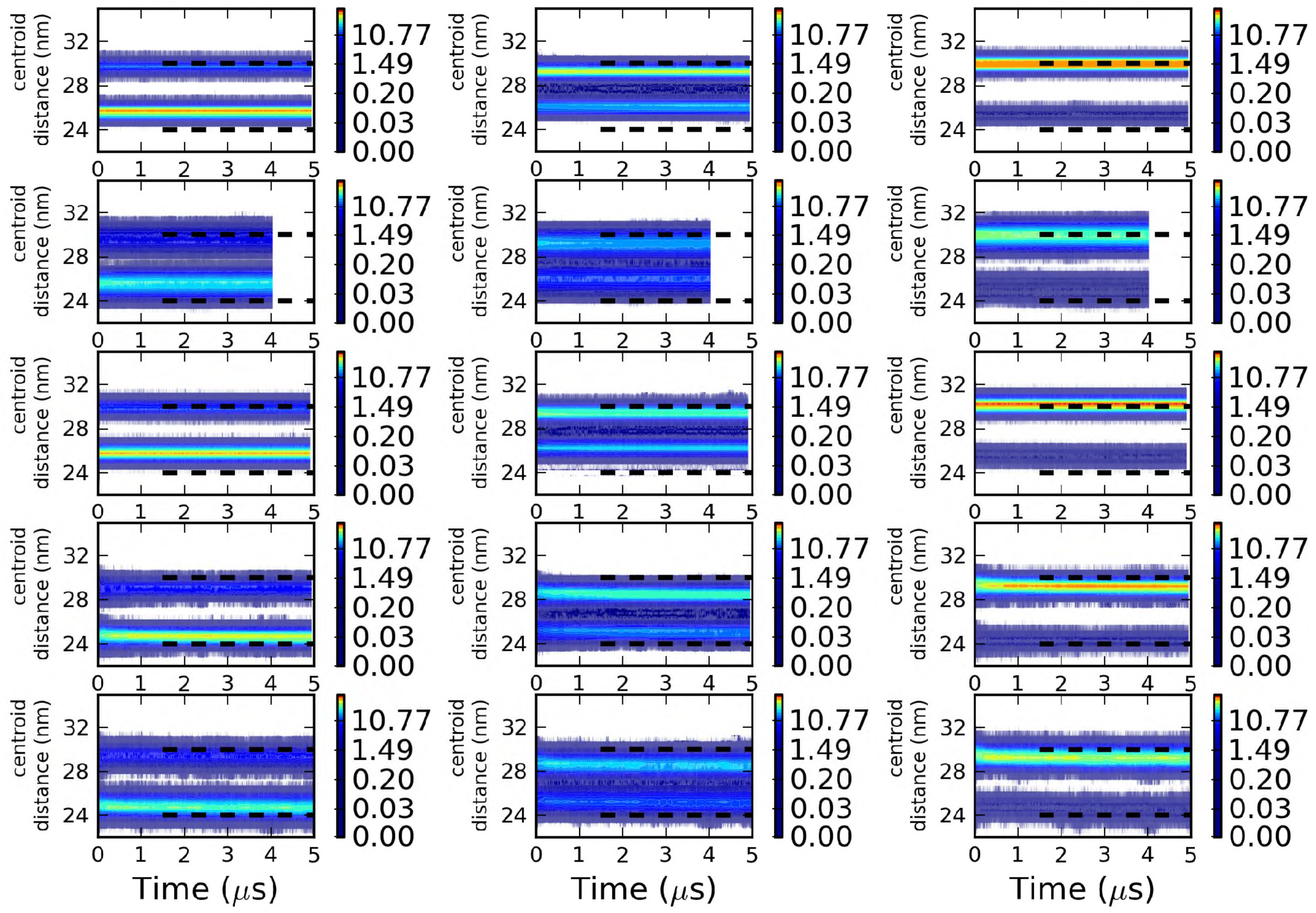

Figure S6: (Related to Figure 5) Comparing the leaflet distribution of lipids in a set of virions. Contour plots of the distance between the lipid species and the virion lipid centroid are shown for representative inner leaflet species (POPS, *left*), central species (CHOL, *middle*), and outer leaflet species (PPCH, *right*), with values categorized according to the % particles at a particular distance. The different virion conditions include unrestrained proteins at 295 K (*first row*), restrained proteins at 295 K (*second row*), unrestrained proteins at 323 K (*third row*), and unrestrained proteins with FORS glycolipid at 295 K (*fourth row*) or 323 K (*fifth row*).

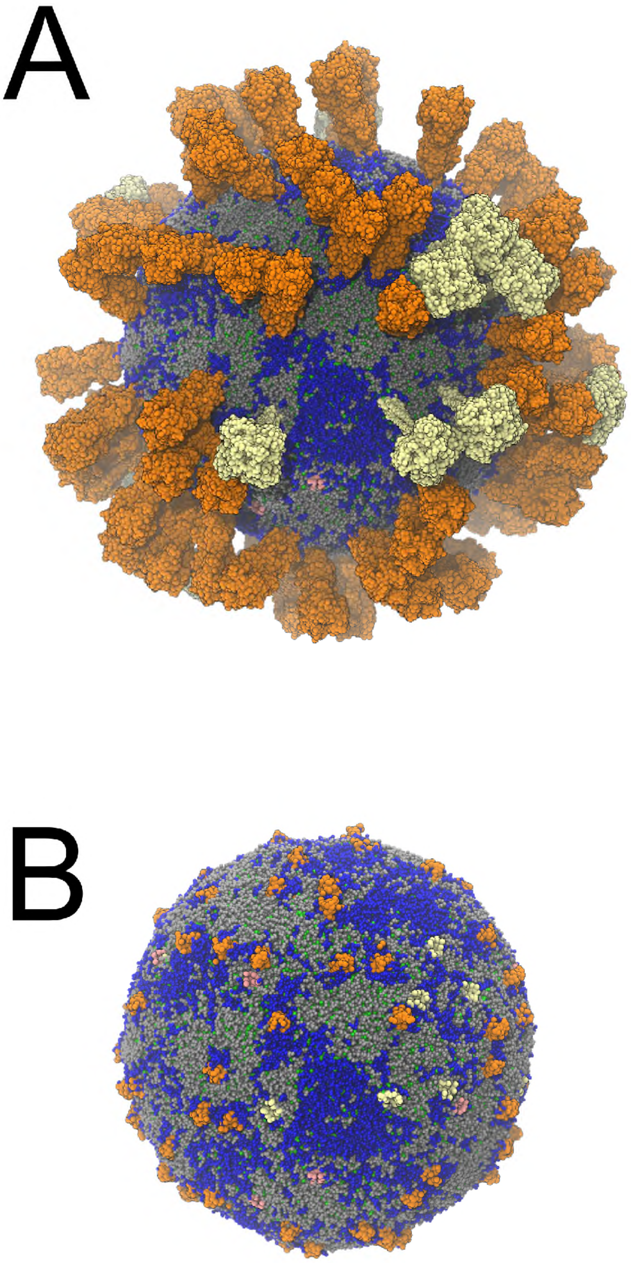

Figure S7: Final snapshots of the 1.8 μs virion production simulation (lipid envelope composition: 40% CHOL, 36% DPPC, 24% DUPC) with (A) or without (B) viral protein ectodomains at 310 K. Species are coloured by the following scheme: HA (*orange*), NA (*white*), M2 (*pink*), DPPC (*grey*), DUPC (*dark blue*), CHOL (*green*). Solvent particles have been excluded for clarity.

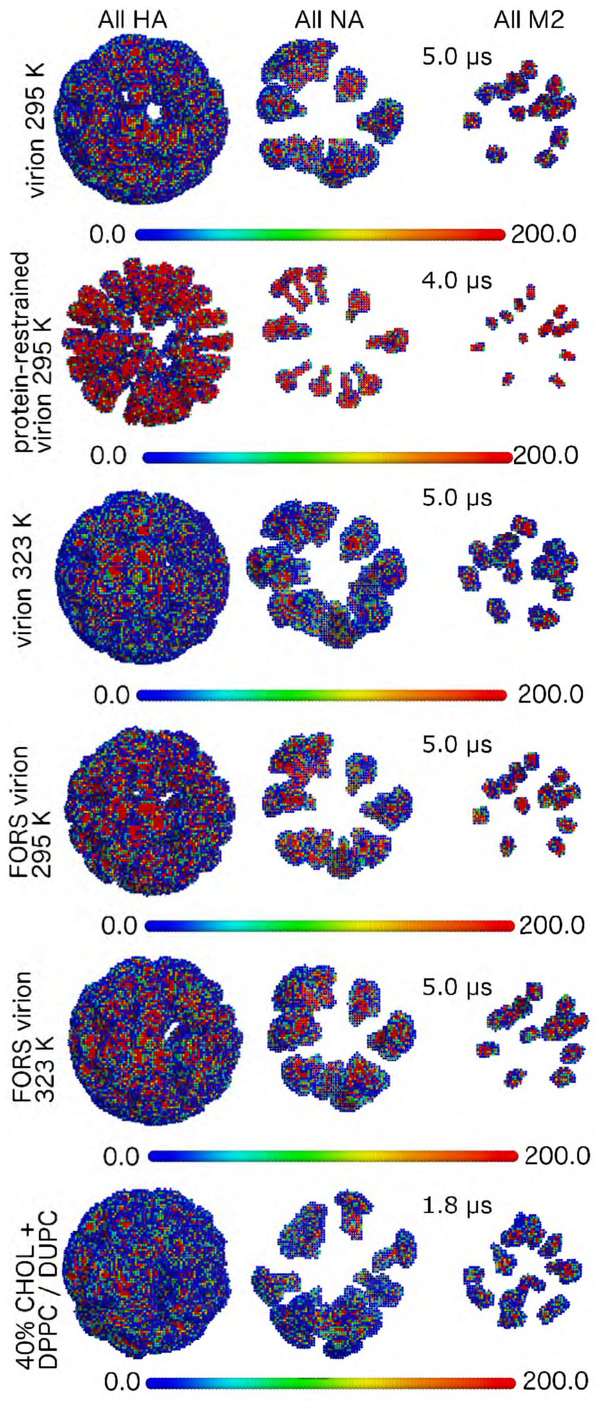

Figure S8: (Related to Figure 6) Assessment of virion protein mobility using contour maps on a linear scale of 0 to 200 counts in 12.5 Å side cubic bins. The counts are for the presence of protein particles accumulated over the total set of frames in a given replicate, with 10000 frames per μs.

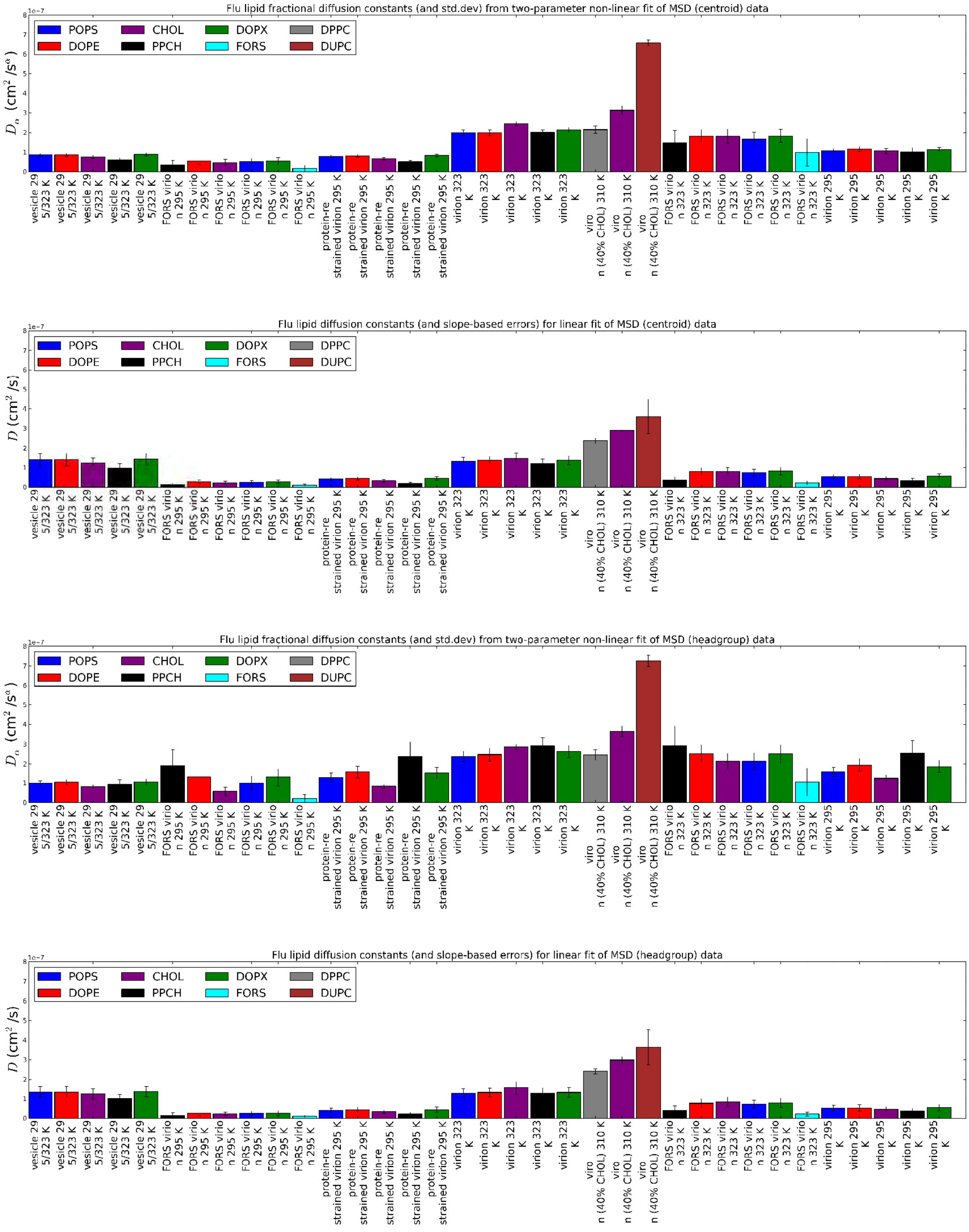

Figure S9: (Related to Figure 7) Comparison of diffusion constants for all lipid species in all influenza A simulation conditions. The constants were calculated based on the centroid of lipid residues (*top*) or on their headgroup particles only (*bottom*), using either two-parameter non-linear fits to the MSD vs time data or using linear fits. Standard deviations are shown for the former fitting procedure while the difference in the slopes of the two halves of the data are used to estimate errors in the latter. 1.8 μs of data were available for the 40% CHOL condition, and approx. 4 μs were available in the presence of protein mobility restraints, otherwise 5 μs trajectories were used.

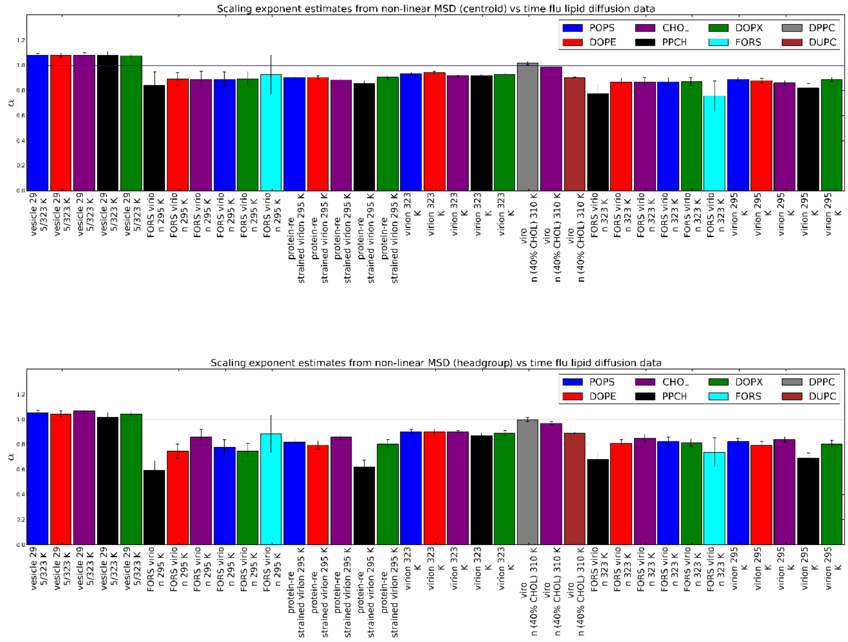

Figure S10: (Related to Figure 8) Comparison of diffusion scaling exponents (α) for all lipid species in all influenza A simulation conditions. α values were calculated based on the centroid of the lipid residues (*top*) or on their headgroup particles only (*bottom*). The non-linear two-parameter fit analysis produces α directly as a fitting parameter. It is possible to estimate the uncertainty as the square root of the diagonal of the covariance matrix from the non-linear fit, producing the standard deviations shown here. A guide line (*blue*) is shown for true random walk diffusion ($\alpha = 1$).

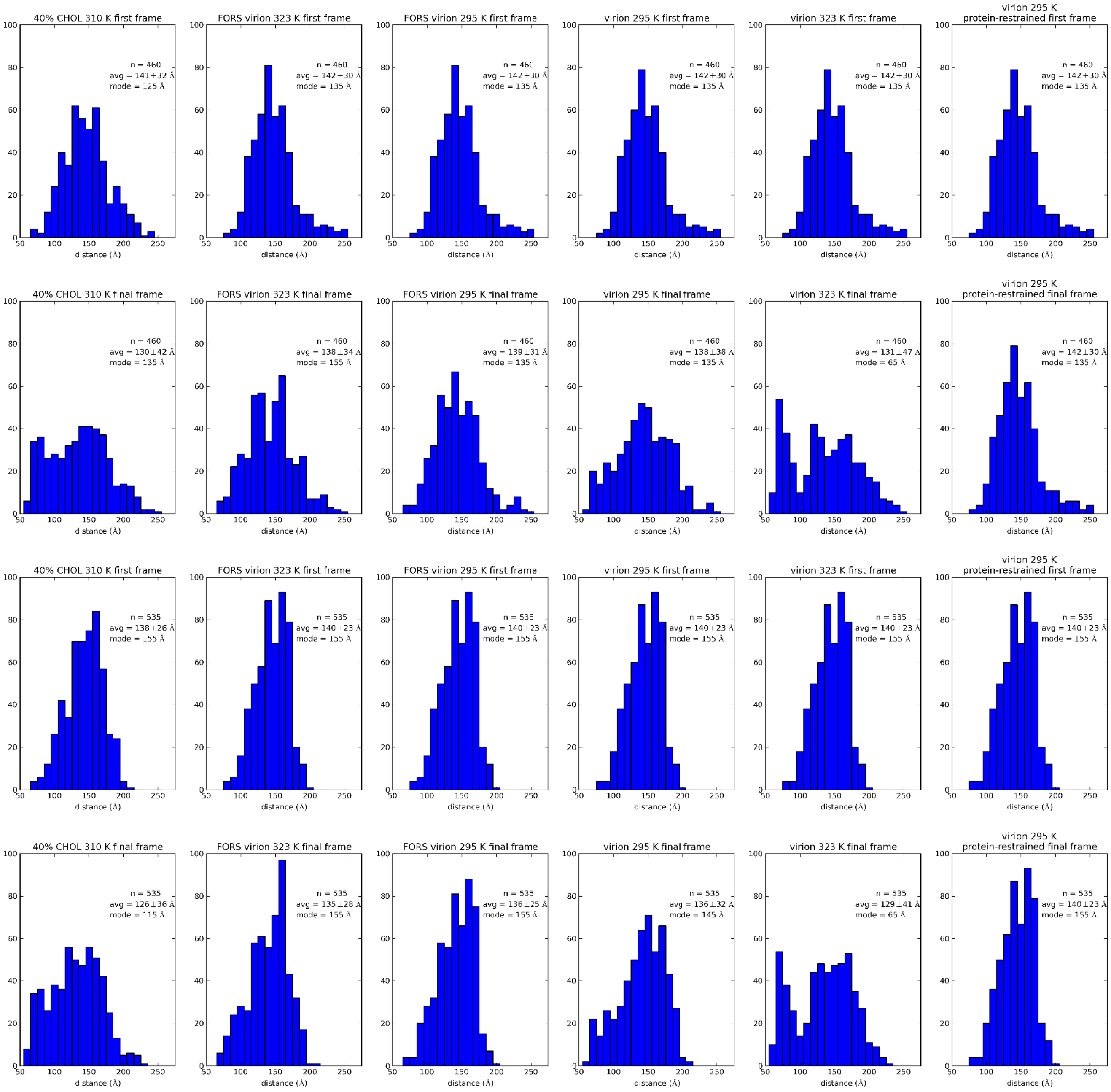

Figure S11: (Related to Figure 9) Comparison of interprotein distance histograms in first and final snapshots of simulations when excluding (*top 2 rows*) or including (*bottom 2 rows*) the M2 proton channel. The centroids of each protein were employed and the top five contacts per protein were included for 460 distances (no M2) or 535 distances (with M2).

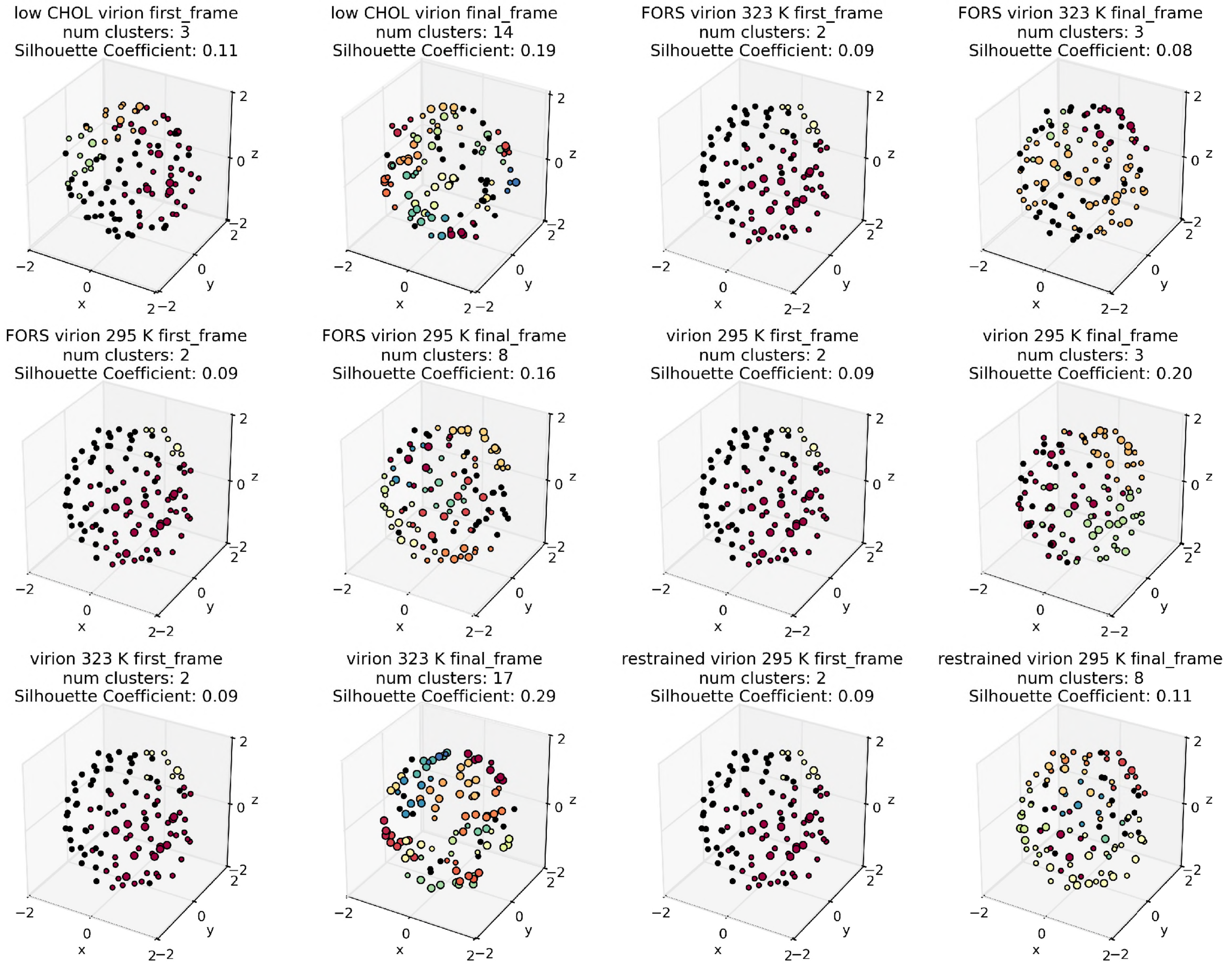

Figure S12: (Related to Figure 9) Clustering of proteins at start and end of influenza A virion simulations using the DBSCAN algorithm. Normalized protein centroid coordinates are coloured based on cluster assignment, with large points representing core samples, small points for edge values, and black dots for coordinates assigned as noise. Silhouette coefficient values closer to 1.0 provide higher confidence in the quality of the cluster assignments.

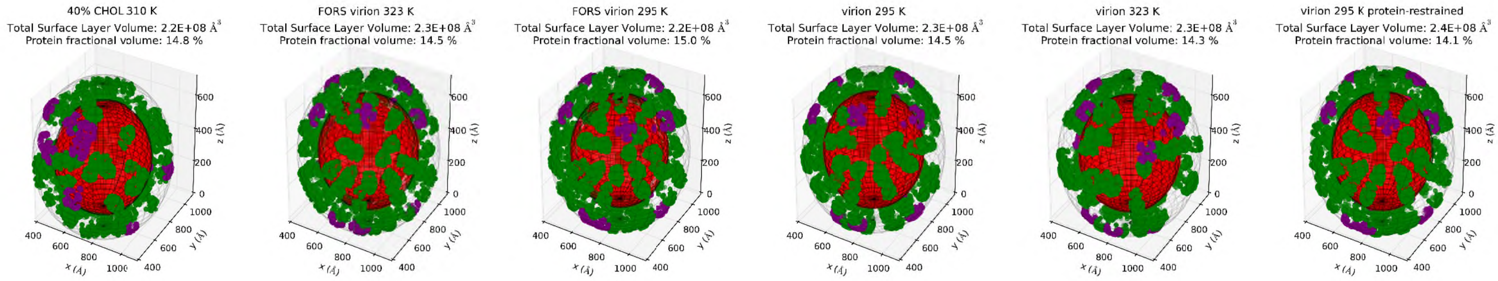

Figure S13: (Related to Figure 9) Assessment of influenza A spike glycoprotein fractional surface volume in final snapshots of production simulations. The phosphate particles in each virion (*red*) were used to define the inner boundary of the outer surface layer (contained within a diffuse meshgrid), while the outer boundary was assigned 13 nm farther from the virion centroid (contained within outermost diffuse meshgrid). The coordinates of particles representing the convex hulls of the spike glycoproteins are shown for HA (*green*) and NA (*purple*). The % of the outer surface layer volume occupied by the spike glycoproteins is indicated above each condition.

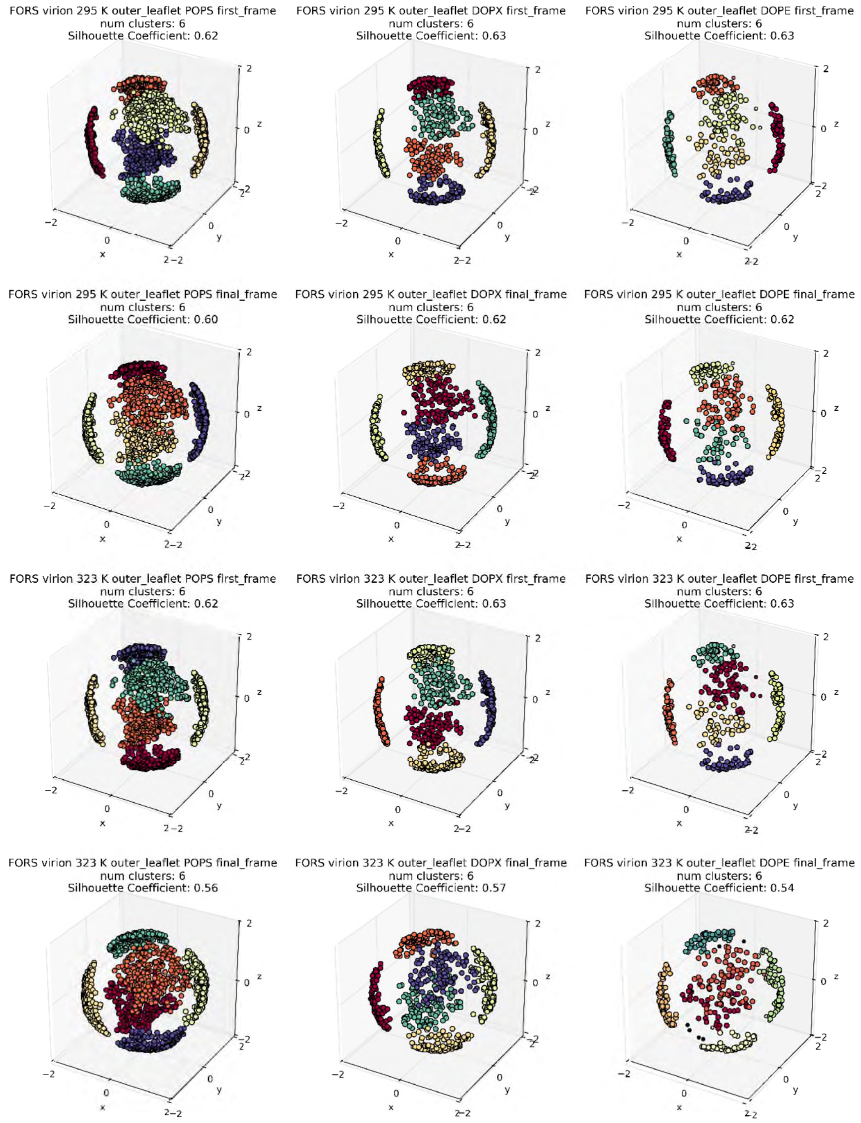

Figure S14: (Related to Figure 9) Clustering of minor outer leaflet lipid species in first and final snapshots of Forssman glycolipid-inclusive virion simulations using DBSCAN algorithm at two temperatures. Plot details as described in Figure S12.

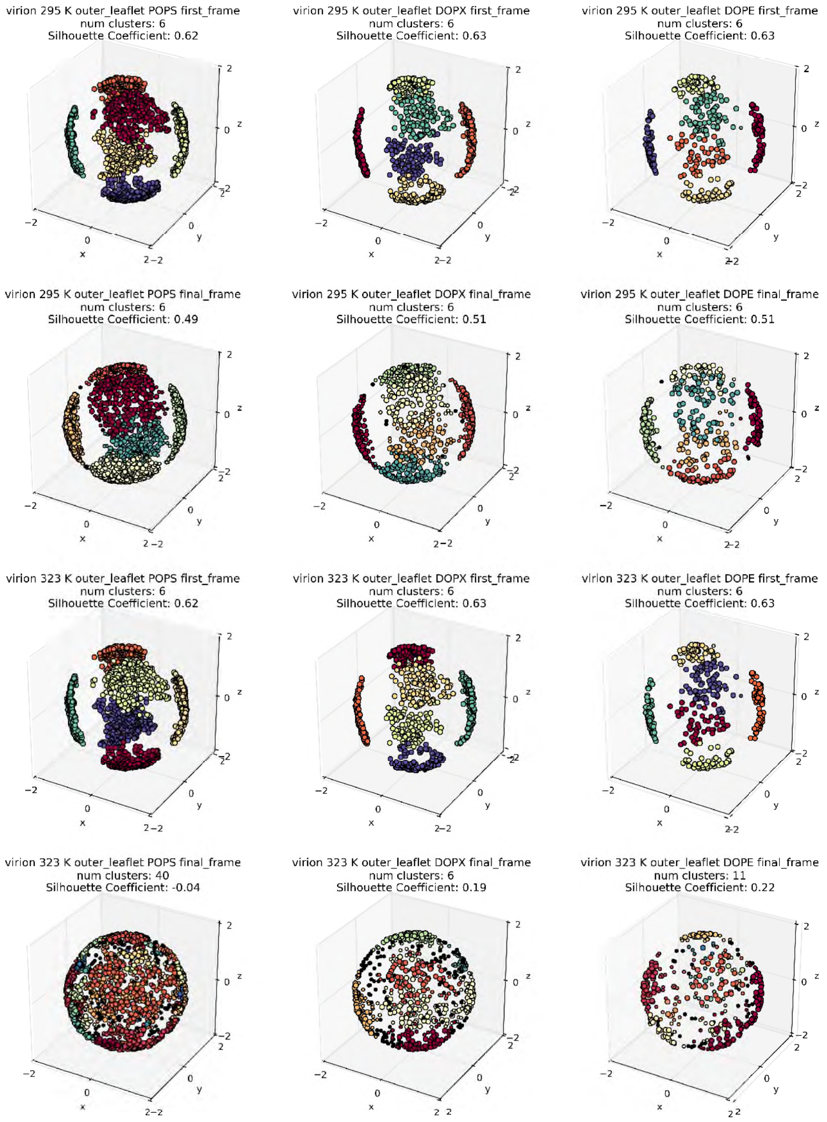

Figure S15: (Related to Figure 9) Clustering of minor outer leaflet lipid species in first and final snapshots of virion simulations lacking the Forssman glycolipid using DBSCAN algorithm at two temperatures. Plot details as described in Figure S12.

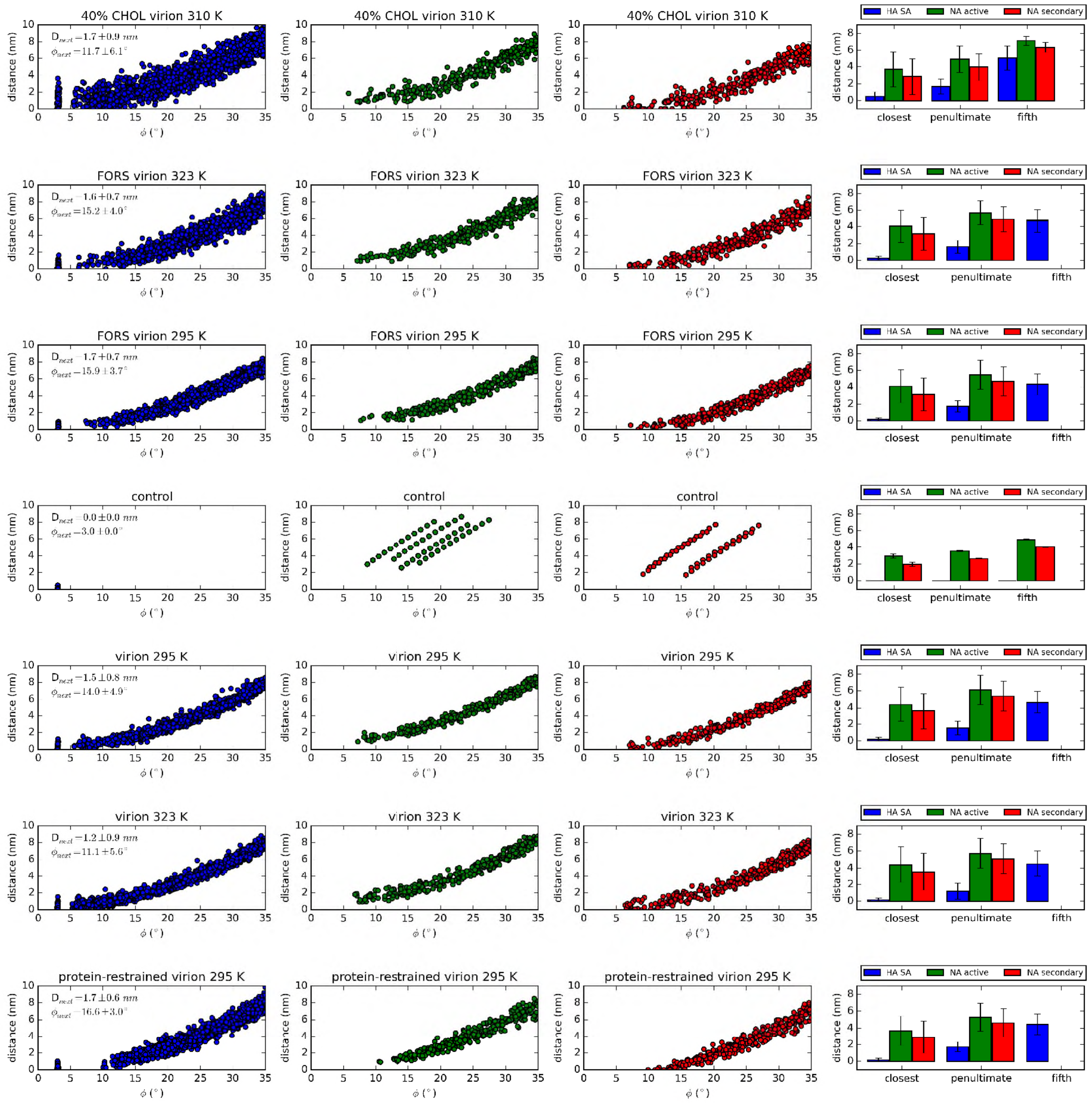

Figure S16: (Related to Figure 10) Geometric constraints on host cell sialic acid binding to HA trimers and NA tetramers on the curved influenza A virion surface at the end of the simulations. The definitions of the ϕ and distance parameters are as demonstrated in Figure 10. For all simulation conditions, the relationship between surface distance and ϕ is plotted for the HA SA binding sites (*blue*), the NA active sites (*green*) and the NA secondary sites (*red*). The data is accumulated over all eighty possible virion-host cell attack orientations where a single HA trimer is aligned along the +Z axis. The bar charts (*fourth column*) summarize the overall average surface distance for the SA binding sites on the closest, penultimate and fifth closest proteins, where available, with their standard deviations. The inset distance and ϕ values for the HA SA site data (*first column*) correspond to the penultimate (second closest) HA trimer, for comparison with experimental values for the closest neighbour HA (Wasilewski et al., 2012). A control condition (*fourth row*) with linear-spaced NA tetramers and superposed HA trimers was used to verify the algorithm.

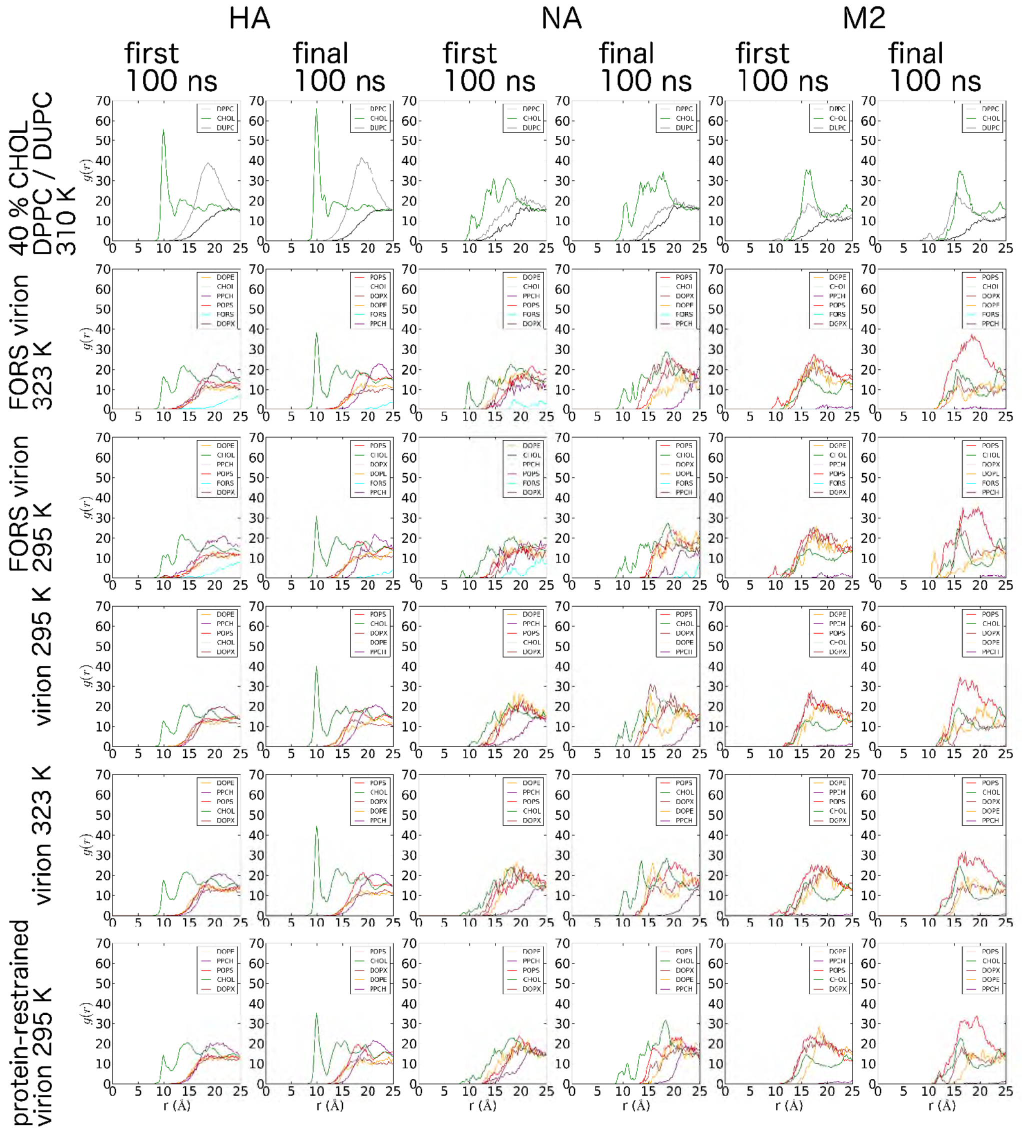

Figure S17: (Related to Figure 5) Radial distribution functions between influenza A protein types (HA, NA, M2) and lipid species compared between first 100 ns and final 100 ns simulation windows for all simulation conditions. The calculations were performed between the TMD region centroids of the biological assemblies (HA trimer, NA tetramer, M2 tetramer) and the centroids of each type of lipid molecule.

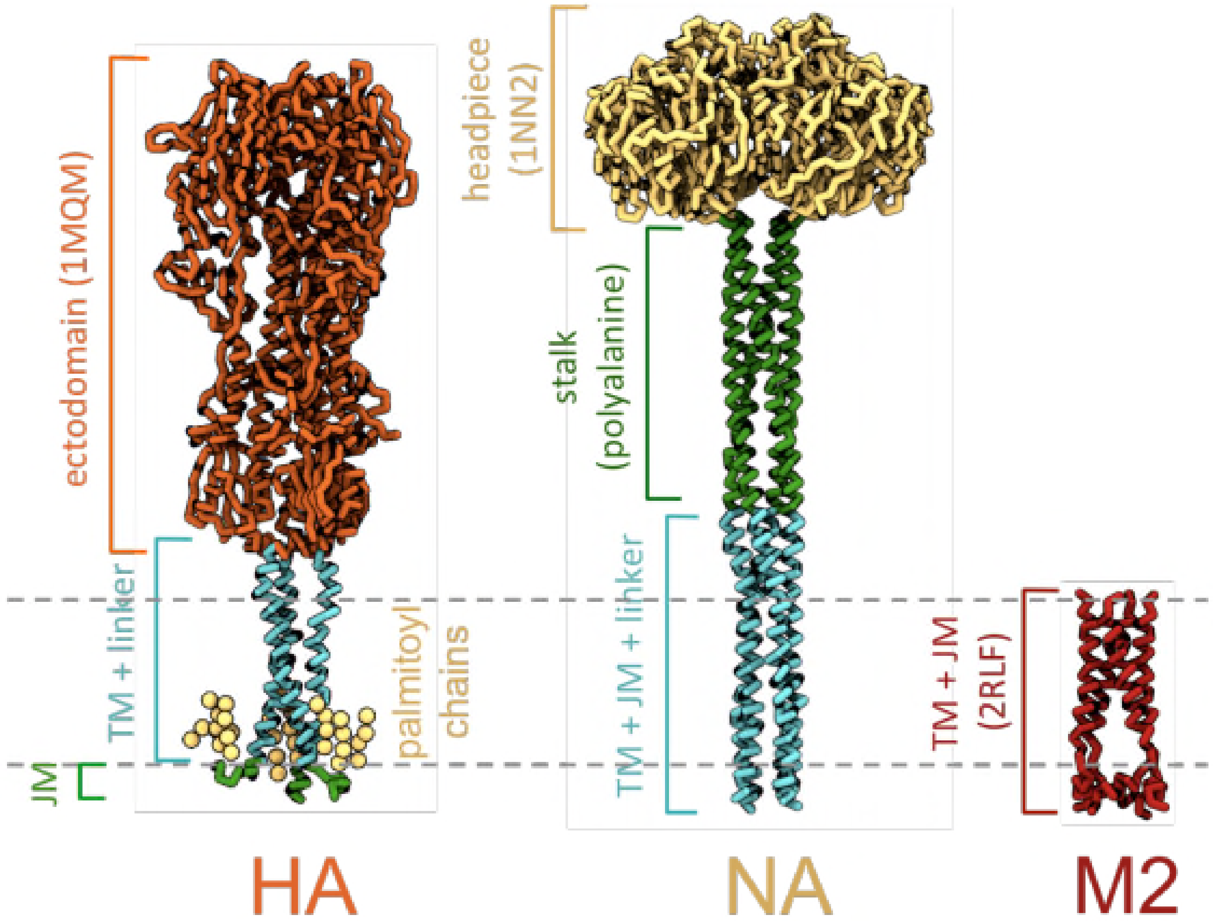

Figure S18: (Related to Figure 1) CG representations of the three species of influenza A envelope protein. The gray broken lines indicate the approximate location of the bilayer headgroups. **HA**: the ectodomain (orange) was derived from the X-ray structure (PDB code: 1MQM) (Ha et al., 2003); the linker and TM domain (cyan) were modeled as α-helix; the C-terminal tail (green) was modeled as an unstructured region with attached palmitoyl tails (yellow). **NA**: the headpiece (yellow) was derived from the X-ray structure (PDB code: 1NN2) (Varghese and Colman, 1991); the stalk (green) was modeled as a polyalanine coiled coil; the linker, TM domain, and N-terminal tail (cyan) were modeled as α-helix. **M2**: the model was derived from the NMR structure (PDB code: 2RLF) (Schnell and Chou, 2008).

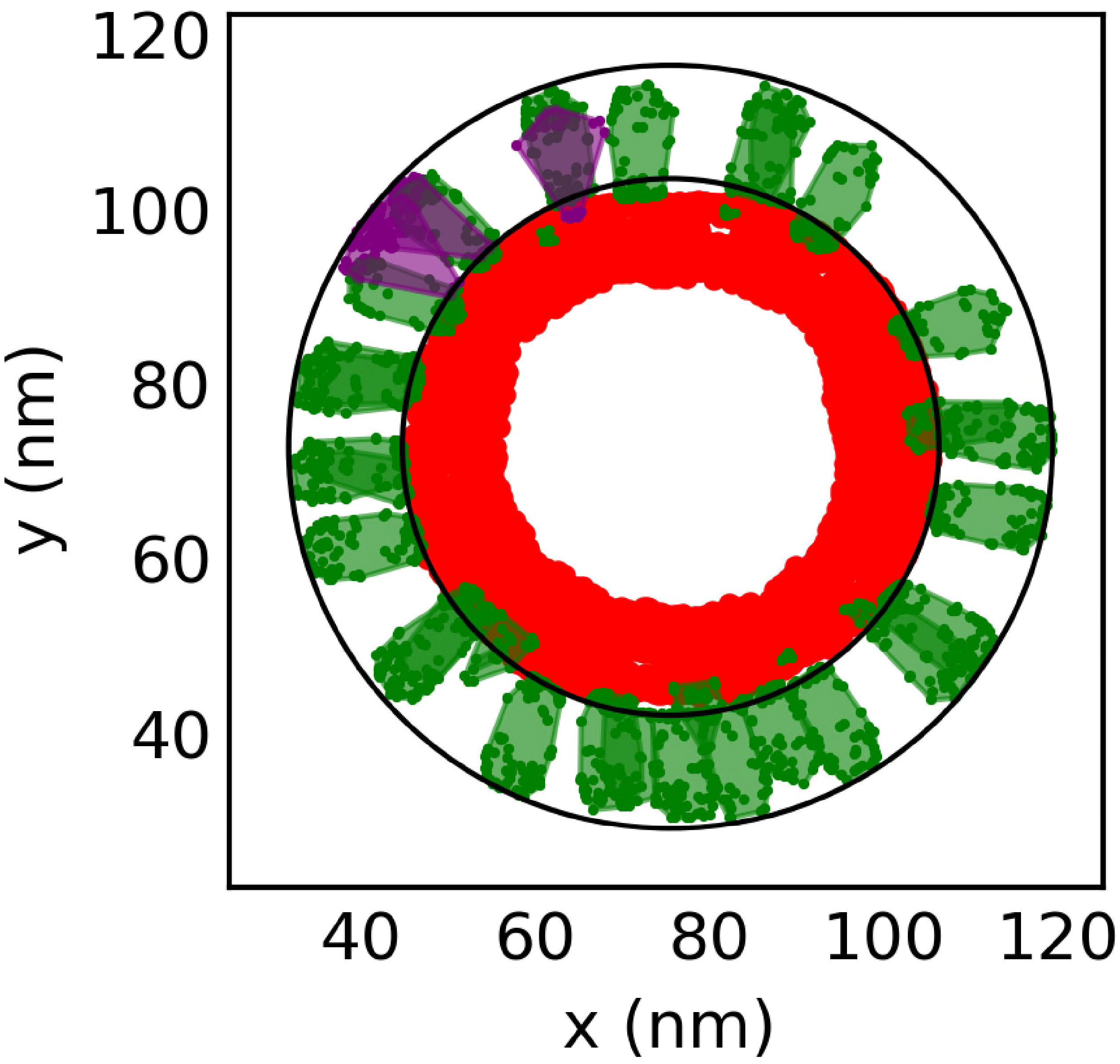

Figure S19: (Related to Figure 9) 20 nm cross-sectional slice through the Z-axis of an influenza A computational model demonstrating the three volumes required to calculate the fraction of the outer surface layer volume (V_f) occupied by the spike glycoproteins. $V_f = (V_{HA} + V_{NA})/V_{ring}$

Supplemental Computational Procedures

Simulation details. The refined procedure for virion model construction starts by alternating Packmol (Martinez et al., 2009) packing steps and GROMACS energy minimization steps to produce a lipid vesicle of the appropriate size and composition. The target starting diameter is actually much larger than the lower bound of the experimental estimate of the outer diameter, in order to avoid severe steric clashes. All vesicle / virion simulations were performed using GROMACS 4.5.5 (Hess et al., 2008) and the MARTINI 2.1 forcefield (Marrink et al., 2007; Monticelli et al., 2008). The forcefield was modified to produce the following custom particles / molecules: the restrained shell particle (RPO: attractive to water, super repulsive to other particles) (Parton, 2011), ether-linked dioleoyl phosphatidylethanolamine (DOPX: mimic ether-linkage at C1 position by changing particle 4 in DOPE from type Na to N0), hydroxylated sphingomyelin (PPCS) (PPCH: mimic hydroxyl group at PPCS particle 5 by adjusting from particle type C1 to Nda). RPO particles were always position-restrained in each dimension with a force constant of 10^3 kJ mol^{-1} nm^{-2}. The Forssman glycolipid was parametrized in two sections. First, the ceramide backbone was based on the matching particles provided for sphingomyelin in the MARTINI forcefield. Second, the headgroup of the Forssman glycolipid was comprised of the monosaccharides glucose, galactose, and N-acetylgalactosamine, with the glycosidic linkages as follows: GalNac-α (1-3) – GalNac-β (1-3) – Gal α (1-4) – Gal β (1-4) – Glc β (1-1) - Cer. The initial monosaccharide parameters were based on the sweet MARTINI forcefield (López et al., 2009) and previous in-house parametrization of the glycolipid GM3 (D.S. and H.K., personal communication). All sugars were initially represented as triangles, consisting of polar (P) particles. The side chains of N-acetylgalactosamine are more complex, and were represented as Nda particles given their similarity to an amino acid backbone. The five headgroup carbohydrates were connected using ‘rotational nodes,’ while the connection between the carbohydrates and ceramide backbone was parametrized by comparison to all-atom simulations using the GLYCAM forcefield (Kirschner et al., 2008). CG particles were mapped onto the atomistic results to compare bonds, angles,

and dihedral angles. Force matching between the two resolution scales was performed to tune the CG force constants for the Forssman glycolipid. Most properties of the atomistic system were successfully reproduced in the CG representation using an iterative refinement process. Similar parametrizations of PIP2 (Stansfeld et al., 2009) and cardiolipin (Dahlberg and Maliniak, 2010) have accurately reproduced lipid binding sites in crystal structures. The HA, NA and M2 proteins were modelled as described previously (Parton, 2011). Production simulations employed 5×10^8 steps with 10 fs time steps, and the composition of each system is summarized in Figure S3. Trajectory coordinates were written every 10^4 steps (every 0.1 ns). Coulomb and VDW interactions were respectively shifted off between 0.0 and 1.2 nm and 0.9 and 1.2 nm. Acylated proteins, lipids, RPO particles, and solvent were separately temperature coupled using the Berendsen algorithm (Berendsen et al., 1984) with a 1.0 ps time constant. Isotropic pressure coupling was performed using the Berendsen algorithm with a 1.1 ps time constant, 1×10^{-6} bar^{-1} compressibility, and a 1.0 bar reference pressure. In the case where all 107 viral membrane proteins were restrained, each of the proteins was subject to center of mass pulling (in all 3 dimensions) using an umbrella potential in GROMACS. The pull force (harmonic force constant, $k = 10^5$ kJ mol^{-1} nm^{-2}) was exerted in the direction of an absolute reference point at the origin, with initial pull vector set to (0,0,0) and pull rate set to 0 nm / ps (immobilized reference) for each protein. Forces and center of mass values from the pull code were written every 10^4 steps (every 0.1 ns).

Protein Models.

HA model.

The model of HA (Figure S18) was that described previously (Parton et al., 2013). Briefly, the model was based on the X-ray structure (PDB code: 1MQM) of the protein from the A/duck/Ukraine/1/63 (H3N8) influenza strain (Ha et al., 2003), which was converted to the CG representation. The X-ray structure does not include

the TM domain or the cytoplasmic domains, or a short linker between the ectodomain and TM domain. This missing region was modeled as α-helix. Palmitoyl chains were added to residues Cys555, Cys562 and Cys565 of the TM domain. The resultant model of the intact HA trimer was then simulated in a bilayer patch, allowing for relaxation of the structure. To maintain the protein tertiary structure, elastic network restraints were applied with the ElNeDyn tool (Periole et al., 2009), using a cutoff of 1.4 nm and force constants of 1000 kJ mol^{-1} nm^{-2}. The cytoplasmic domain was treated as unstructured and excluded from the restraint network.

NA model.

The NA stalk domain was modelled as a polyalanine coiled coil (based on a tetrameric coiled coil motif from the GCN4 leucine zipper protein (PDB code: 1GCL (Harbury et al., 1993))), with its length (approx. 10 nm) matched to cryo-EM images of the protein (Harris et al., 2006). In reality, the stalk domain of NA is thought to be mostly unstructured, with inter-subunit disulfide bonds stabilizing the tetrameric arrangement (Blok and Air, 1982). However, the exact sequence is largely unconserved and mutations of long stretches can be accepted without compromising the viability of the protein or virus (Luo et al., 1993). In contrast, the length of the stalk is thought to be an important factor; long deletions attenuate the growth rate and alter the host specificity of the virus (Castrucci and Kawaoka, 1993; Luo et al., 1993; Matsuoka et al., 2009).

M2 model.

The M2 proton channel is a 97-residue tetrameric TM protein. The CG model was derived directly from a NMR structure of the protein in a micellar environment (PDB code: 2RLF) (Schnell and Chou, 2008). This structure was solved for a construct comprising residues 18-60, which includes 15 residues of the C-terminal cytoplasmic

tail as well as the α-helical TM domain. The protein is normally palmitoylated at C50 (Sugrue et al., 1990), but this residue was mutated to a serine in the construct used to derive the NMR structure. Palmitoyl chains were thus not included in the CG model. Palmitoylation has been found to have no effect on viral replication in vitro, although it may provide some contribution to virulence in vivo (Grantham et al., 2009).

Analysis of trajectories. Particle coordinates in trajectory files were exposed using the open source Python MDAnalysis library (Michaud-Agrawal et al., 2011) and analyzed using in-house Python code. We also used IPython (Pérez and Granger, 2007), numpy (Oliphant, 2007), SciPy (Jones et al., 2001–), scikit-learn (Pedregosa et al., 2011) and matplotlib (Hunter, 2007) for scientific computing in Python. VMD (Humphrey et al., 1996) and PyMOL (Schrödinger, LLC, 2010) were used for visualization.

Incorporation of viral proteins into equilibrated vesicle. Models of the HA, NA and M2 proteins were obtained as previously described (Parton et al., 2013; Parton, 2011). Two coordinate files were used to start the procedure. The first contained the equilibrated vesicle and its RPO core with solvent removed from the system. The second contained only proteins: 80 HA trimers, 12 NA tetramers, and 15 M2 tetramers, with an approximately equidistant distribution about the surface of the equilibrated vesicle. The distribution and alignment of proteins was performed using a point-charge separation-based approach (Parton, 2011; Tatham, 2013). The two coordinate files were merged to produce a single set of coordinates with the appropriately-placed proteins *superposed* on the equilibrated vesicle. The merged coordinates were adjusted such that the principal axis of the first protein membrane-embedding candidate (1 of the 107 proteins) was aligned along the positive Z-axis along with the center of the RPO core placed at the origin. At this stage, all proteins except the embedding candidate are stripped from the coordinate file and their coordinates are stored. However, in subsequent embedding rounds, all proteins which are already embedded are permitted to remain in the sytem for the embedding process, otherwise the lipids may collpase back into the previously-occupied spaces. In

short, only 1 *superposed* protein is permitted in the system for embedding–the protein to be embedded. The g_membed program (Wolf et al., 2010) was then used to embed a given protein in the vesicle membrane using a fractional xy starting size of 0.1 and expanding to full horizontal size in 1000 steps, using a probe radius of 0.8 nm. NVT conditions (rather than NPT) were employed for embedding to preserve the box dimensions and simplify the reintegration of the remaining *superposed* coordinates back to the newly-embedded system, until all proteins were embedded.

Forssman glycolipid incorporation with Alchembed. An unpublished in-house procedure (named Alchembed) was used to exploit the free energy machinery (Beutler et al., 1994) of GROMACS for progressive activation of the non-bonded interactions of FORS glycolipid residues. 1000 molecular dynamics steps using a 1 fs integration timestep were performed with an increase in scaling factor ($\Delta\lambda$) of 10^{-3} per step. With λ starting at zero, the particles of the FORS glycolipid were therefore gradually incorporated into the sytem until both VDW and Coulomb intermolecular interactions were fully activated ($\lambda = 1$). Nonbonded interactions between and within FORS particles were also switched off initially and gradually activated during the simulation. Position restraints were applied only to the ceramide backbone of FORS with a force constant of 5 kJ mol^{-1} Å^{-2} and to RPO with a force constant of 10 kJ mol^{-1} Å^{-2}. Protein, lipid, RPO and FORS particles were separately temperature-coupled to an external bath using the Berendsen thermostat at 323 K with a time constant of 1.0 ps. The Berendsen barostat was used for isotropic pressure coupling at a reference pressure of 1.0 bar, a compressibility of 10^{-6} bar^{-1} and a coupling constant of 1.1 ps. The soft-core alpha parameter was 0.5 and the power for λ in the soft-core function was 1.0.

Sphericity tracking analysis. Sphericity (Ψ) was formally defined as a measure of the roundness of a particle in the context of geology (Wadell, 1935) (Equation (1)).

$$\Psi = \frac{\pi^{\frac{1}{3}}(6V_p)^{\frac{2}{3}}}{A_p} \tag{1}$$

Here, V_p is the volume of the particle and A_p its surface area. For every tenth frame (every 1 ns) of each vesicle or virion trajectory, the full set of lipid particle coordinates was selected. The convex hull of the lipid coordinates, conceptually similar to the outer surface coordinates, was calculated using an algorithm implemented in SciPy (Jones et al., 2001–), which exposes the vertices of the triangular facets of the convex polytope. The coordinates of these vertices were translated such that the Cartesian centroid of all facet vertices was at the origin. The surface area (A_p) of the convex hull was calculated by summing the areas from all triangular facets in a given frame. Each triangular facet was then treated as the base of a tetrahedron with an apex at the origin. As the system has been translated to place the origin within the convex hull, the sum of the absolute values of the volumes of each component tetrahedron can be used to calculate the volume (V_p) of the convex hull (*i.e.,* estimate volume bounded by lipids). Each individual tetrahedral volume is calculated based on a linear determinant of the triangular facet coordinates, which is simpler with a vertex at the origin (Equation (2)). The sphericity value in a given frame was then calculated using Equation (1).

$$V_p = \frac{1}{6}\begin{vmatrix} x_1 & y_1 & z_1 \\ x_2 & y_2 & z_2 \\ x_3 & y_3 & z_3 \end{vmatrix} \tag{2}$$

Outer diameter tracking. At 100 frame (10 ns) intervals, the full set of hydroxylated sphingomyelin (PPCH) molecule PO_4 particles was selected. Another selection made for each parsed frame was for all particles excluding the RPO core. The centroid of the latter set of particles was taken to represent the centroid of the vesicle (centroid of all lipids) or virion (centroid of all lipids + proteins) in a given frame, with RPO particles

having been excluded because the RPO core is often not centrally located due to drift of the virion relative to this restrained body of particles. A distance matrix was calculated between the full set of phosphate particles and the centroid. Double the average value of all phosphate radial distances was taken as an estimate of the outer diameter of the vesicle or virion.

Stratification tracking analysis. For every frame (every 0.1 ns) of each parsed replicate trajectory, the centroid of all lipid species in the system was determined. The PO_4 particles of a given lipid species or the ROH particles of CHOL were also selected. A distance matrix was calculated between the full set of PO_4 or ROH particles and the lipid centroid of the system. These radial distances were histogrammed in 0.5 nm bins between 0 and 80 nm, and converted to values as % of the given lipid population within a particular radial distance bin. The % values were countour plotted using logarithmic levels of base 2 varying between powers of -8.0 and 6.0.

Protein mobility tracking. The mobility of each of the 107 membrane proteins in flu virions at 295 K, and with the presence or absence of restraints on protein motion, was analysed in every 100 frames (every 10 ns) of the relevant trajectories. 107 protein centroids were determined in each parsed frame to simplify the tracking of any given protein, and there was no correction for any minor rotational or translational motion of the overall virion. The analysis was substantially more efficient as a result of manual indexing of the coordinate arrays rather than using built-in MDAnalysis selection and centroid functions.

Protein positional probability 3D mapping. For every frame (every 0.1 ns) in every parsed virion trajectory, the protein coordinates were obtained. The coordinate arrays for each protein type were individually histogrammed over 80 bins in each dimension of the full systems–100 nm side-length cubes (each bin is a cube with 12.5 Å side-length). After the first frame, coordinate histograms were still produced, but were combined with the histrogram from the previous frame. Compounding histograms on a per-frame basis vastly reduces the memory consumption of the code.

Diffusion analysis. Lipid and protein mean square displacement (MSD) values were calculated over a range of window sizes: 1, 3, 5, 10, 25, 50, 100, 200, 300, 400, and 500 ns. Diffusion constants were estimated from a least squares first degree (linear) polynomial fit of the MSD vs time data, either using the full complement of window sizes or excluding potentially ballistic motion at smaller window sizes ($\leq$ 50 ns). 50 ns is well outside the previously-described cutoff (30 ns) for anomalous diffusion in CG lipid simulations (Goose and Sansom, 2013). The uncertainty in the calculated diffusion constants was estimated as the difference of the linear slopes from the first and second halves of the data, which is the approach used by the GROMACS tools function g_msd. Lipid diffusion constants were calculated using both the centroid of all particles in each lipid or using only a single CG headgroup particle. For protein diffusion constant calculations, only the centroids of the biological assemblies were used. The scaling exponent (α) was estimated as the slope of the log MSD vs log time data. A second set of diffusion calculations were performed assuming anomalous diffusion ($\alpha \neq 1$) by non-linear least squares fitting to the two parameter equation (3) described previously (Kneller et al., 2011):

$$\text{MSD} = 4D_\alpha t^\alpha, 0 < \alpha < 2 \tag{3}$$

Where D_α is measured in units of length^2/time$^\alpha$. The standard deviations of both parameters were taken from the square root of the diagonal of the covariance matrix from the non-linear least squares fit.

Radial Distribution Functions. Radial distribution functions (RDFs) were calculated between lipid tail particles and bulk solvent molecules in each of the influenza CG-MD simulation replicates for between the final 2 ns and final 114 ns. The variety of time windows is the result of compromise for the extremely large system size and distance matrices which make the calculations extremely slow. The lipid residues and their assigned tail particles for the purposes of the RDFs: DUPC (C4B), DPPC (C4B), POPS (C5B), DOPX (C5B), DOPE (C5B), FORS (C4B), and PPCH (C4B). CHOL was not included in these analyses. Solvent selections were

limited to W, WF or ION particles within 100.0 Å of the tail particles in each frame (to manage the size of the distance matrix). Lipid-solvent distances were categorized into one of 200 bins between 0 and 110.0 Å, with calculations following multicore and normalization strategies described previously (Levine et al., 2011; Michaud-Agrawal et al., 2011), and with verification of results against GROMACS tools function g_rdf for a single frame. A similar algorithm was employed for calculation of RDFs between the TMD region centroids of the three types of influenza protein (HA, NA, M2) and the centroids of the lipid molecules of each type in each condition, for both the first and last 100 ns of a given simulation.

Sialic acid binding site surface distance and angle calculations. Three sialic acid (SA) binding sites per HA trimer were assigned based on the proximity of residues to (or their direct inclusion in) the canonical 220 loop (Q226, P227, G228), 130 loop (S136, S137, A138) or 190 helix (Y98, W153, H183) (Ha et al., 2003). The overall centroid of the pertinent CG residue centroids in a given monomer was used to define a single coordinate representing the SA binding site. Although SA surface distance and angle calculations have been described for filamentous virions (Wasilewski et al., 2012), our algorithm for spherical virions was slightly different. All coordinates were translated such that the centroid of all virion lipid particles was placed at the origin. Then an iterative procedure was applied to each of the 80 HA trimers on the surface of a given virion in a snapshot taken near the end of the simulation. First, the centroid of the three SA sites in the HA trimer is calculated. The angle and axis of rotation are calculated for alignment with the +Z axis for the vector passing through the origin and the reference SA site centroid. After applying the same coordinate transformation to the other SA site coordinates a planar surface is placed at the maximum of all SA site Z coordinates. For all 240 SA binding sites in the current reference frame, the restriction on rotation $\phi \leq 35^{\circ}$ relative to the reference HA axis was enforced to match the previous calculations on filamentous virions. The surface distance was calculated as the difference in Z coordinates between the planar surface and the SA site that falls within the ϕ restriction region. For each of the 80 attack angles of a given virion the six closest SA binding site distances and their

corresponding rotations (ϕ) were recorded and final average and standard deviation values are reported after splitting to the top three (closest HA) and next three (closest neighbour HA) SA binding site surface distances. Final reported distance and ϕ values represent an accumulation over all 80 HA reference frames (virion attack orientations). We also performed a similar calculation that included the NA active and secondary sites that can associate with SA (Colman et al., 1983; Sung et al., 2010).

Spike glycoprotein fractional surface volume calculation. We defined the outer surface of a given virion in the final frame of its trajectory as the space bounded between the outer leaflet and a perimeter 13 nm (length of 1 HA molecule) beyond, for consistency with previous experimental data (Wasilewski et al., 2012). The radius of the outer leaflet was defined as the average of the 20 largest CG PO_4 particle distances from the centroid of all lipids in the virion. The total volume of the outer surface layer was then calculated as the difference in the volumes of the two bounding spheres. We calculated the convex hulls of the CG particles representing the spike glycoproteins, excluding any CG particles that fall outside the region bounded by the two spheres. The volumes of the convex hulls of the spike glycoproteins (HA and NA) were individually calculated by summing the volumes of tetrahedra encompassing a facet of the convex hull with a vertex at the origin, similar to the process described for the sphericity calculations. The sum of all spike glycoprotein volumes was represented as a % of the total volume of the outer surface layer (Figure S19).

Protein separation distances on virion surfaces. Two separate virion surface interprotein distance calculations were performed–in the presence or absence of the M2 protein, primarily because the simulations allow for access to the positions of M2 proteins while the published experimental separation data does not (Wasilewski et al., 2012). In either case, the full distance matrix between all proteins, specifically the centroids of their constituent CG particles, was calculated in the first and final frames of a given simulation. The distances to the five closest neighbouring proteins were stored for each individual protein on the virion surface, and categorized

into one of 20 bins in the distance range bounded by 50 and 250 Å for the histograms reported in this work. The average, standard deviation, mode and total number of distances used for the full set of closest five neighbour calculations of a given virion are also reported.

Protein and lipid clustering analysis. The protein clustering analysis was performed on the full set of 107 influenza membrane protein centroids, and was not subdivided by protein type, for the first and final time points of each protein-inclusive simulation condition. The data was preprocessed by removing the mean and scaling to unit variance. The DBSCAN clustering algorithm implemented in scikit-learn (Pedregosa et al., 2011) was applied over the parameter value ranges of $0.2 \leq \epsilon < 1.2$ in increments of 0.1 and $3 \leq min\_samples < 12$ in increments of 1. The best result was selected on the basis of the maximal silhouette coefficient (s) calculated by the scikit-learn metrics library, where $-1 \leq s \leq 1$, and negative values indicate problematic cluster assignments, values near 0 indicate overlapping clusters, and values closer to 1 indicate a higher confidence in cluster assignments. The optimal cluster assignments were only analyzed and plotted if more than one cluster could be assigned for a given condition. A similar algorithm was employed for the clustering analysis of lipids, except that lipids were categorized based on both their species and their leaflet in the viral envelope. The leaflet assignments of lipid species were performed based on distance of headgroup particles from virion centroid, with inner leaflet within 27 nm and outer leaflet beyond that distance.

Supplemental References

Berendsen, H., Postma, J., Vangunsteren, W., Dinola, A., and Haak, J. (1984). Molecular-dynamics with coupling to an external bath. J. Chem. Phys. *81*, 3684–3690.

Beutler, T.C., Mark, A.E., van Schaik, R.C., Gerber, P.R., and van Gunsteren, W.F. (1994). Avoiding singularities and numerical instabilities in free energy calculations based on molecular simulations. Chemical Physics Letters *222*, 529 – 539.

Blok, J., and Air, G.M. (1982). Variation in the membrane-insertion and stalk sequences in eight subtypes of influenza type A virus neuraminidase. Biochemistry *21*, 4001–4007.

Castrucci, M.R., and Kawaoka, Y. (1993). Biologic importance of neuraminidase stalk length in influenza A virus. J. Virol. *67*, 759–764.

Colman, P.M., Varghese, J.N., and Laver, W.G. (1983). Structure of the catalytic and antigenic sites in influenza virus neuraminidase. Nature *303*, 41–44.

Dahlberg, M., and Maliniak, A. (2010). Mechanical properties of coarse-grained bilayers formed by cardiolipin and zwitterionic lipids. J. Chem. Theory Comput. *6*, 1638–1649.

Gerl, M.J., Sampaio, J.L., Urban, S., Kalvodova, L., Verbavatz, J.M., Binnington, B., Lindemann, D., Lingwood, C.A., Shevchenko, A., Schroeder, C., *et al.* (2012). Quantitative analysis of the lipidomes of the influenza virus envelope and MDCK cell apical membrane. J. Cell Biol. *196*, 213–221.

Goose, J.E., and Sansom, M.S.P. (2013). Reduced lateral mobility of lipids and proteins in crowded membranes. PLoS Comput. Biol. *9*, e1003033.

Grantham, M.L., Wu, W.H., Lalime, E.N., Lorenzo, M.E., Klein, S.L., and Pekosz, A. (2009). Palmitoylation of

the influenza A virus M2 protein is not required for virus replication in vitro but contributes to virus virulence. J. Virol. *83*, 8655–8661.

Ha, Y., Stevens, D.J., Skehel, J.J., and Wiley, D.C. (2003). X-ray structure of the hemagglutinin of a potential H3 avian progenitor of the 1968 hong kong pandemic influenza virus. Virology *309*, 209 – 218.

Harbury, P., Zhang, T., Kim, P., and Alber, T. (1993). A switch between two-, three-, and four-stranded coiled coils in gcn4 leucine zipper mutants. Science *262*, 1401–1407.

Harris, A., Cardone, G., Winkler, D.C., Heymann, J.B., Brecher, M., White, J.M., and Steven, A.C. (2006). Influenza virus pleiomorphy characterized by cryoelectron tomography. Proc. Natl. Acad. Sci. U. S. A. *103*, 19123–19127.

Hess, B., Kutzner, C., van der Spoel, D., and Lindahl, E. (2008). Gromacs 4: Algorithms for highly efficient, load-balanced, and scalable molecular simulation. J. Chem. Theory Comput. *4*, 435–447.

Humphrey, W., Dalke, A., and Schulten, K. (1996). VMD – Visual Molecular Dynamics. J. Mol. Graphics *14*, 33–38.

Hunter, J.D. (2007). Matplotlib: A 2d graphics environment. Comput. Sci. Eng. *9*.

Jones, E., Oliphant, T., Peterson, P., *et al.* SciPy: Open source scientific tools for Python (2001–). URL `http://www.scipy.org/`.

Kirschner, K.N., Yongye, A.B., Tschampel, S.M., González-Outeirio, J., Daniels, C.R., Foley, B.L., and Woods, R.J. (2008). GLYCAM06: A generalizable biomolecular force field. Carbohydrates. J. Comput. Chem. *29*, 622–655.

Kneller, G.R., Baczynski, K., and Pasenkiewicz-Gierula, M. (2011). Communication: Consistent picture of

lateral subdiffusion in lipid bilayers: Molecular dynamics simulation and exact results. J. Chem. Phys. *135*, 141105.

Levine, B.G., Stone, J.E., and Kohlmeyer, A. (2011). Fast analysis of molecular dynamics trajectories with graphics processing units-radial distribution function histogramming. J. Comput. Phys. *230*, 3556 – 3569.

López, C.A., Rzepiela, A.J., de Vries, A.H., Dijkhuizen, L., Hnenberger, P.H., and Marrink, S.J. (2009). Martini coarse-grained force field: Extension to carbohydrates. J. Chem. Theory Comput. *5*, 3195–3210.

Luo, G., Chung, J., and Palese, P. (1993). Alterations of the stalk of the influenza virus neuraminidase: deletions and insertions. Virus Res. *29*, 141–153.

Marrink, S.J., Risselada, H.J., Yefimov, S., Tieleman, D.P., and de Vries, A.H. (2007). The MARTINI force field: coarse grained model for biomolecular simulations. J. Phys. Chem. B *111*, 7812–7824.

Martinez, L., Andrade, R., Birgin, E.G., and Martinez, J.M. (2009). PACKMOL: a package for building initial configurations for molecular dynamics simulations. J Comput Chem *30*, 2157–2164.

Matsuoka, Y., Swayne, D.E., Thomas, C., Rameix-Welti, M.A., Naffakh, N., Warnes, C., Altholtz, M., Donis, R., and Subbarao, K. (2009). Neuraminidase stalk length and additional glycosylation of the hemagglutinin influence the virulence of influenza H5N1 viruses for mice. J. Virol. *83*, 4704–4708.

Michaud-Agrawal, N., Denning, E.J., Woolf, T.B., and Beckstein, O. (2011). MDAnalysis: A toolkit for the analysis of molecular dynamics simulations. J. Comput. Chem. *32*, 2319–2327.

Monticelli, L., Kandasamy, S.K., Periole, X., Larson, R.G., Tieleman, D.P., and Marrink, S.J. (2008). The MARTINI Coarse-Grained Force Field: Extension to Proteins. J. Chem. Theory Comput. *4*, 819–834.

Oliphant, T.E. (2007). Python for scientific computing. Comput. Sci. Eng. *9*.

Parton, D.L., Tek, A., Baaden, M., and Sansom, M.S.P. (2013). Formation of raft-like assemblies within clusters of influenza hemagglutinin observed by MD simulations. PLoS Comput. Biol. *9*, e1003034.

Parton, D. (2011). Pushing the boundaries: molecular dynamics simulations of complex biological membranes. PhD thesis (Oxford, Oxon : University of Oxford).

Pedregosa, F., Varoquaux, G., Gramfort, A., Michel, V., Thirion, B., Grisel, O., Blondel, M., Prettenhofer, P., Weiss, R., Dubourg, V., *et al.* (2011). Scikit-learn: Machine learning in Python. Journal of Machine Learning Research *12*, 2825–2830.

Pérez, F., and Granger, B.E. (2007). IPython: a system for interactive scientific computing. Comput. Sci. Eng. *9*, 21–29.

Periole, X., Cavalli, M., Marrink, S.J., and Ceruso, M.A. (2009). Combining an elastic network with a coarse-grained molecular force field: Structure, dynamics, and intermolecular recognition. J. Chem. Theory Comput. *5*, 2531–2543.

Schnell, J.R., and Chou, J.J. (2008). Structure and mechanism of the M2 proton channel of influenza A virus. Nature *451*, 591–595.

Schrödinger, LLC. The PyMOL molecular graphics system, version 1.3r1 (2010.).

Stansfeld, P.J., Hopkinson, R., Ashcroft, F.M., and Sansom, M.S.P. (2009). PIP2-Binding Site in Kir Channels: Definition by Multiscale Biomolecular Simulations. Biochemistry *48*, 10926–10933.

Sugrue, R.J., Belshe, R.B., and Hay, A.J. (1990). Palmitoylation of the influenza A virus M2 protein. Virology *179*, 51–56.

Sung, J.C., Wynsberghe, A.W.V., Amaro, R.E., Li, W.W., and McCammon, J.A. (2010). Role of secondary sialic acid binding sites in influenza N1 neuraminidase. J. Am. Chem. Soc. *132*, 2883–2885.

Tatham, S. Simon Tatham's Home Page (2013). URL `http://www.chiark.greenend.org.uk/~sgtatham/`. [Online; accessed 24-June-2013].

Varghese, J., and Colman, P. (1991). Three-dimensional structure of the neuraminidase of influenza virus A/Tokyo/3/67 at 2.2 Å resolution. Journal of Molecular Biology *221*, 473–486.

Wadell, H. (1935). Volume, shape, and roundness of quartz particles. J. Geol. (Chicago, IL, U. S.) *43*, 250–280.

Wasilewski, S., Calder, L.J., Grant, T., and Rosenthal, P.B. (2012). Distribution of surface glycoproteins on influenza A virus determined by electron cryotomography. Vaccine *30*, 7368–7373.

Wolf, M.G., Hoefling, M., Aponte-Santamaría, C., Grubmüller, H., and Groenhof, G. (2010). g_membed: Efficient insertion of a membrane protein into an equilibrated lipid bilayer with minimal perturbation. J. Comput. Chem. *31*, 2169–2174.
